# Pentaphosphorylation via the Anhydride of Dihydrogen Pentametaphosphate: Access to Nucleoside Hexa- and Heptaphosphates and Study of Their Interaction with Ribonuclease A

**DOI:** 10.1021/acscentsci.4c00835

**Published:** 2024-07-15

**Authors:** Gyeongjin Park, Evans C. Wralstad, Noelia Faginas-Lago, Kevin Qian, Ronald T. Raines, Giovanni Bistoni, Christopher C. Cummins

**Affiliations:** † Department of Chemistry, Massachusetts Institute of Technology, Cambridge, Massachusetts 02139, United States; ‡ Department of Chemistry, Biology,and Biotechnology, University of Perugia, 06123, Perugia, Italy

## Abstract

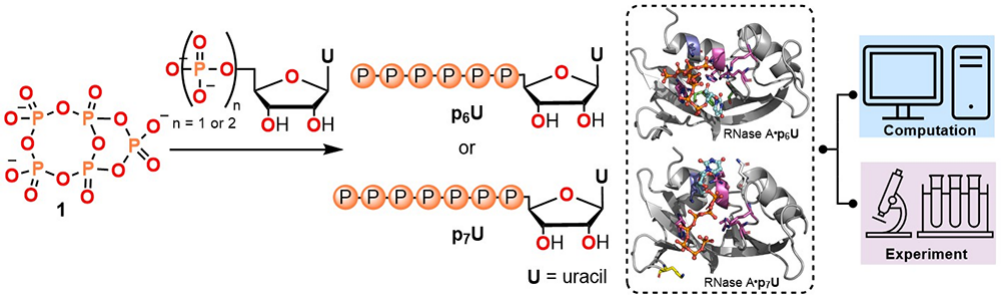

Pentametaphosphate is the little studied cyclic pentamer of the metaphosphate ion, [PO_3_]_5_^5–^. We show that the doubly protonated form of this pentamer can be selectively dehydrated to provide the anhydride [P_5_O_14_]^3–^ (**1**). This trianion is the well-defined condensed phosphate component of a novel reagent for attachment of a pentaphosphate chain to biomolecules all in one go. Here, we demonstrate by extending adenosine monophosphate (AMP) and uridine monophosphate (UMP) to their corresponding nucleoside hexaphosphates, while adenosine diphosphate (ADP) and uridine diphosphate (UDP) are phosphate chain-extended to the corresponding nucleoside heptaphosphates. Such constructs are of interest for their potential biological function with respect to RNA-processing enzymes. Thus, we go on to investigate in detail the interaction of the polyanionic constructs with ribonuclease A, a model protein containing a polycationic active site and for which X-ray crystal structures are relatively straightforward to obtain. This work presents a combined experimental and quantum chemical approach to understanding the interactions of RNase A with the new nucleoside hexa- and heptaphosphate constructs.

## Abstract 2

Pentaphosphorylation methodology enables synthesis of novel polyanionic nucleoside hexa- or heptaphosphates, the interaction of which with ribonuclease A is elucidated via theory and experiment.

## Introduction

Condensed phosphates consist of multiple phosphate residues linked by energy-rich phosphoanhydride bonds and function in diverse biological roles, including energy storage^1−3^ and cell signaling pathways.^4^ Biomolecules, such as nucleosides, peptides, or proteins, may be covalently modified via conjugation to an oligophosphate, sparking interest in such constructs as biochemical tools or possible natural products.^5−14^ Thus, the following synthetic chemistry question arises naturally: how might one efficiently attach a specific-length oligophosphate chain to a biomolecule of interest? Although methods exist for extending nucleoside or peptide monophosphates to pyro- or triphosphates,^15−24^ the challenge intensifies for longer oligophosphates due to the fact that extant processes are iterative, requiring sequential addition of one phosphate group at a time.^25,26^ Therefore, we are targeting reagents and methods enabling the installation of oligophosphate chains, of a given desired chain length, in a minimal number of synthetic steps. We have previously developed phosphorus(V)-based reagents and methods for tri- and tetraphosphorylation, enabling attachment of a tri- or tetraphosphate chain in a single operation.^24,27−29^

In this article, we detail the synthesis and utility of [PPN]_2_[TBA]**1** as a reagent in the first direct chemical syntheses of nucleoside hexa- and heptaphosphates by attaching a pentaphosphate chain to the anhydrous nucleoside mono- and diphosphates in noniterative procedures. It is noteworthy that the pentaphosphorylation reactions using **1** proceed optimally in conjunction with the novel use of an organometallic promoter, Mo(NCMe)_3_(CO)_3_. With the new chain-extended nucleoside oligophosphates in hand, we go on to investigate in detail their interaction with ribonuclease A, a model protein containing a polycationic active site and for which X-ray crystal structures are relatively straightforward to obtain. Previously, we showed that uridine pentaphosphate is a potent noncovalent inhibitor of RNase A.^27^

**Figure 1:**
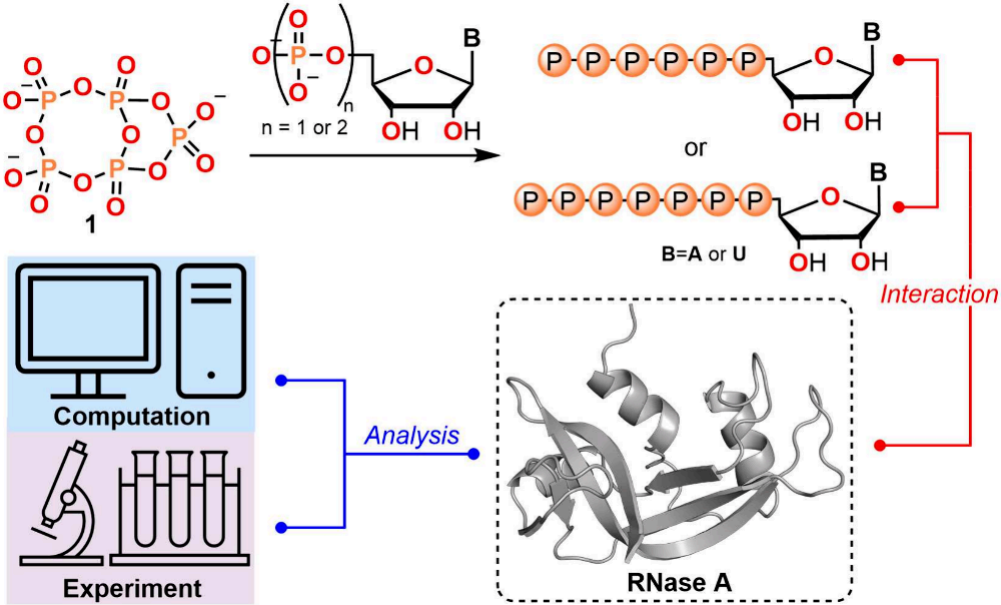
This work: syntheses of nucleoside hexa- and heptaphosphates and graphical representation of the experimental and computational studies of their interactions with RNase A.

To better comprehend RNase A and nucleoside oligophosphate interactions, we examine the correlation between the experimental and calculated protein–ligand interactions (Figure 1) by combining efforts in crystallographic and computational investigations in the present work. For the computational study, we developed a workflow relying on quantum chemical simulations that considers all protein–ligand adducts without any simplification. All parts of each system were treated using electronic structure methods within the ONIOM framework.^30^ The ligand and the enzymic active site are described using a local variant of the “gold standard” coupled cluster approach with singles, doubles, and perturbatively included triples excitations, CCSD(T). Unlike the traditional approach to studying protein–ligand interactions with molecular dynamics and molecular docking simulations, our workflow relies on crystallographic data and electronic structure methods to develop an understanding of the key interactions in the crystal and is not biased by the use of an empirically parametrized force field. The combination of the CCSD(T) approach with the appropriate energy decomposition scheme enables deciphering the influence of the oligophosphate chain length and of individual polypeptide and nucleoside residues on ligand binding.

## Results and Discussion

**Figure 2:**
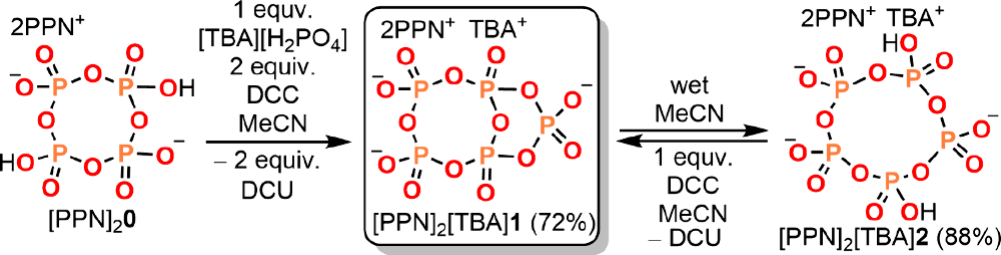
Syntheses of the anhydride of dihydrogen pentametaphosphate (**1**) and dihydrogen pentametaphosphate (**2**). (PPN = bis(triphenylphosphine)iminium, TBA = tetra-*n*-butylammonium, DCC = *N*,*N*′-dicyclohexylcarbodiimide and DCU = *N*,*N*′-dicyclohexylurea).

Now, we introduce the anhydride of dihydrogen pentametaphosphate, **1**, obtained by treating dihydrogen tetrametaphosphate, **0**,^31^ with dihydrogen orthophosphate and DCC as shown in Figure 2. Further treatment of [PPN]_2_[TBA]**1** with wet acetonitrile (H_2_O content ≤0.1 w/w%) rehydrates the anhydride to generate dihydrogen pentametaphosphate, **2**, the doubly protonated cyclic pentamer. The overall process **0** → **2** constitutes the net insertion of a single metaphosphate (PO_3_^−^) monomeric unit into the cyclic tetramer, expanding it to the cyclic pentamer.

The acid form of pentametaphosphate (**2**, [P_5_O_15_H_2_]^3–^) appears not to have been reported previously, and even the reported syntheses of TBA^+^ and NH_4_^+^ salts of pentametaphosphate, [PO_3_]_5_^5−^, suffer from tedious iterative purification procedures.^32,33^ In contrast, the synthesis herein reported (Figure 2) is selective and presents no purification or separation challenges. Dihydrogen pentametaphosphate **2** is facilely dehydrated back to **1** upon treatment with DCC.

### Synthesis of the Pentametaphosphate Anhydride Reagent, [PPN]_**2**_[TBA]**1**

**Figure 3:**
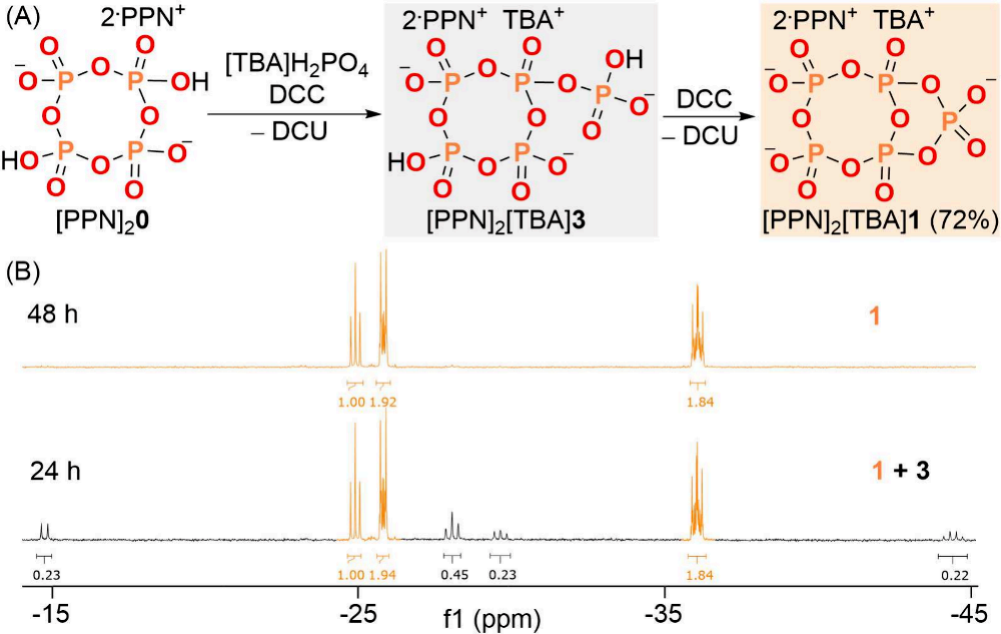
(A) Synthesis of [PPN]_2_[TBA]**1**. (B) ^31^P{^1^H} NMR spectra of this reaction for 24 h and 48 h. Gray box: the intermediate, **3**. Orange box: the product, **1**.

The reaction of [PPN]_2_**0** with [TBA]H_2_PO_4_ in the presence of the dehydrating agent DCC afforded in 24 h a mixture of [PPN]_2_[TBA]**1** and an intermediate, [PPN]_2_[TBA]**3**^28^ (Figure 3). After 48 h, the mixture was fully converted into [PPN]_2_[TBA]**1** as evidenced by the disappearance of the ^31^P{^1^H} NMR resonances previously observed (the terminal phosphate at −15 ppm, three cyclic phosphates at −26 ppm and −27 ppm, and −43 ppm at the branched phosphate).^28^ Although the ^31^P{^1^H} NMR resonances of **1** are quite diagnostic, we attempted to obtain single crystals to elucidate its structure. However, **1** readily transitioned into an oil phase when attempting to get single crystals; consequently, we were unable to determine the X-ray structure of **1**. We are engaged in ongoing efforts to obtain the X-ray structure of **1** through the exploration of various countercation substitutions.

### Syntheses of Nucleoside 5′-Hexa- and Heptaphosphates

Initially we attempted direct reactions with anhydrous NMPs, DCC, dihydrogen tetrametaphosphate, **0**, and dihydrogen pentametaphosphate, **2**, but they did not yield the desired products; instead, [P_4_O_11_]^2–^ or [P_5_O_14_]^3–^(**1**) formed as major species. Therefore, we decided to utilize the ring-strained internal anhydride bond of [PPN]_2_[TBA]**1** to effect NMP conjugation. In contrast with the more reactive tetrametaphosphate anhydride reagent, [PPN]_2_[P_4_O_11_],^27^ the internal α, γ-anhydride bond of [PPN]_2_[TBA]**1** is unreactive toward anhydrous nucleoside monophosphates (NMPs) or nucleoside diphosphates (NDPs) in the absence of a suitable promoter. Typical promoters, such as ZnCl_2_ or MgCl_2_,^15,19,23^ failed to cleanly initiate the ring-opening reaction of **1** with anhydrous NMPs or NDPs. Our previous results^34^ regarding the coordination of tri- and tetrametaphosphate ions to molybdenum complexes prompted us to investigate the ability of molybdenum to coordinate to **1** and subsequently turn on its ability to effect pentaphosphorylation. In the presence of Mo(NCMe)_3_(CO)_3_,^35^ [PPN]_2_[TBA]**1** readily reacted with anhydrous NMPs and NDPs, yielding the corresponding hexa- and heptaphosphates after subsequent hydrolysis.

**Figure 4:**
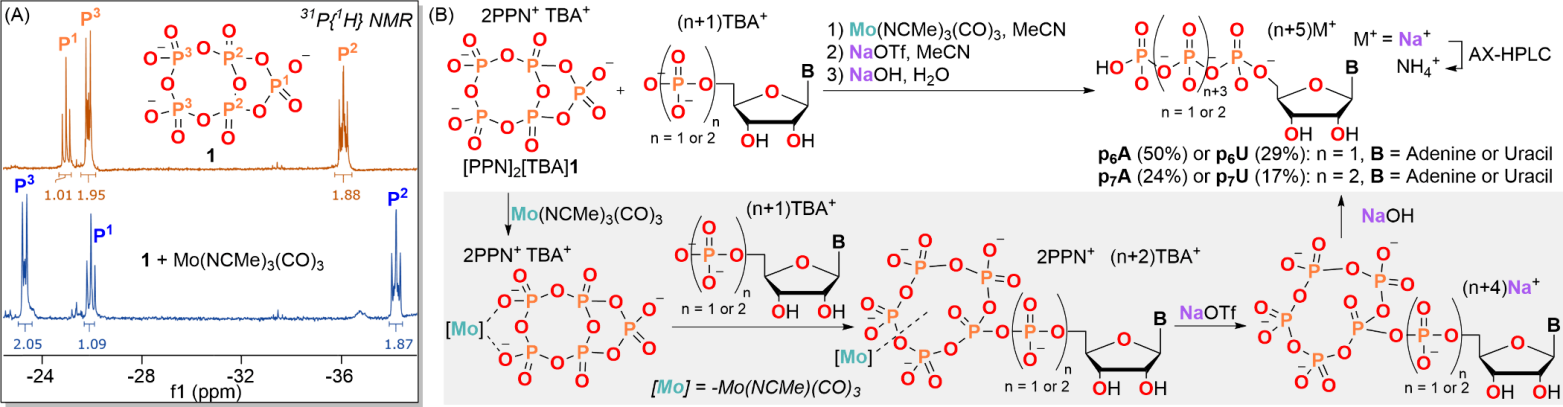
(A) ^31^P{^1^H} NMR spectra of **1** and **1** + Mo(NCMe)_3_(CO)_3_. (B) Synthesis of **p**_**6**_**A**, **p**_**6**_**U**, **p**_**7**_**A**, and **p**_**7**_**U**. Gray box: stepwise reactions from [PPN]_2_[TBA]**1** coordinated with [Mo] to **p**_**6**_**A**, **p**_**6**_**U**, **p**_**7**_**A**, and **p**_**7**_**U**. **A** refers to adenosine, and **U** refers to uridine.

The disparity of the reaction in the presence/absence of the molybdenum complex led us to investigate the coordination of **1** to molybdenum by means of ^31^P{^1^H} NMR spectroscopy. Based on the previously reported changes of ^31^P{^1^H} NMR chemical shifts for tri- and tetrametaphosphates upon binding to Mo complexes,^34^ we propose that only two phosphate groups of **1** coordinate to the molybdenum according to the ^31^P{^1^H} NMR spectrum in Figure 4(A) and SI. We now tested three molybdenum complexes (Mo(NCMe)(CO)_5_,^36^ Mo(NCMe)_3_(CO)_3_, and Mo(NBD)(CO)_4_^37^) with varying numbers of labile ligands on the molybdenum center. Mo(NCMe)(CO)_5_ was found to be unable to promote phosphorylation (see SI), while the molybdenum complexes having equal to or greater than two labile coordination sites promote the desired reaction with anhydrous NMP or NDP nucleophiles.

Subsequently, we optimized the basic hydrolytic ring-opening reactions of the obtained pentametaphosphate nucleoside mono- and diphosphate conjugates (Figure 4(B), gray box center and right). This optimization included a cation exchange to the sodium salts to increase the water solubility, followed by treatment with aqueous sodium hydroxide to open the pentametaphosphate ring (see SI). Anion-exchange HPLC purification of the resulting compounds provided the ammonium salts of adenosine/uridine hexaphosphates (50% yield for **p**_**6**_**A** and 29% for **p**_**6**_**U**) and adenosine/uridine heptaphosphates (24% yield for **p**_**7**_**A** and 17% for **p**_**7**_**U**). This result represents the first direct chemical syntheses of nucleoside-5′-hexa- and -heptaphosphates. Although the dimerizations of ADP to bis(adenosine)-5′-pentaphosphate (**Ap**_**5**_**A**) and ATP to bis(adenosine)-5′-heptaphosphate (**Ap**_**7**_**A**) have been reported,^38^ that strategy only accessed odd numbers of phosphate groups. The hexa- and heptaphosphate nucleosides were obtained in lower yields as compared with the tetra- and pentaphosphate nucleosides that we obtained previously using [PPN]_2_[P_4_O_11_] as a tetraphosphorylation reagent.^27^ The relatively low yields obtained in the present case is attributable, at least in part, to the competing formation of the complex [Mo(P_3_O_9_)(CO)_3_]^3–^,^34^ and some product loss during the cation exchange process. Additionally, we found that the hydrolytic ring-opening process exhibits less selectivity when leading to the nucleoside heptaphosphates as compared with that leading to the nucleoside hexaphosphates, with observed formation of pentametaphosphate and NDPs. Aqueous solutions of the purified nucleoside hexa- and heptaphosphates were observed to be stable for at least a week, as confirmed by ^31^P{^1^H} NMR spectroscopy.

### Experimental Studies of RNase A Interactions with 5′-Nucleoside Oligophosphates

With the new nucleoside hexa- and heptaphosphates in hand, we next measured their inhibition capacities toward RNase A. We previously showed that 5′-uridine nucleotides exhibit increasing inhibition capacity as the phosphate chain length increases from **pU** to **p**_**5**_**U**(27) as shown in Table 1.

**Table 1:** Inhibition Constants (*K*_*i*_) of 5′-Nucleoside Oligophosphates for RNase A

entry	compound	*K*_*i*_ (μM)	ref
1	**pU**	4000	(43)
2	**p**_**2**_**U**	650	(44)
3	**p**_**3**_**U**	8.3 ± 0.3	(27)
4	**p**_**4**_**U**	1.8 ± 0.1	(27)
5	**p**_**5**_**U**	0.068 ± 0.007	(27)
6	**p**_**6**_**U**	1.1 ± 0.2	this work
7	**p**_**7**_**U**	1.0 ± 0.2	this work
8	**pA**	170 ± 6	(27)
9	**p**_**2**_**A**	1.2	(45)
10	**p**_**3**_**A**	0.86	(46)
11	**p**_**4**_**A**	2.1 ± 0.2	(27)
12	**p**_**5**_**A**	1.4 ± 0.06	(27)
13	**p**_**6**_**A**	3.6 ± 0.6	this work
14	**p**_**7**_**A**	2.6 ± 0.4	this work

In a preliminary experiment, we used ^31^P{^1^H} NMR spectroscopy to determine RNase A-oligophosphate affinity, inspired by the research of the Bowman-James group.^39−41^ However, for the purpose of simplification, we assumed a 1:1 binding mode between RNase A and our ligand in this paper. We applied a modified NMR titration method from supramolecular chemistry to measure binding constants for oligophosphates.^42^ The **cP**_**4i**_ (P_4_O_12_^4−^, tetrametaphosphate) affinity of RNase A was found to be 4.25 × 10^4^ M^–1^through direct titration. To measure the RNase A affinity of **p**_**3**_**A** (also known as ATP), a competition titration with RNase A·**cP**_**4i**_ was used due to the sensitivity limitations of direct ^31^P{^1^H} NMR spectroscopy. The binding constants of **p**_**5**_**U**, **p**_**6**_**U**, and **p**_**7**_**U** were determined using a competition titration with RNase A·**p**_**3**_**A**. The binding constants for **p**_**5**_**U**, **p**_**6**_**U**, and **p**_**7**_**U** were found to be 1.86 × 10^7^ M^–1^, 4.74 × 10^7^ M^–1^ and 8.94 × 10^6^ M^–1^, respectively, indicating that **p**_**6**_**U** demonstrates the maximum affinity enhancement.

**Figure 5:**
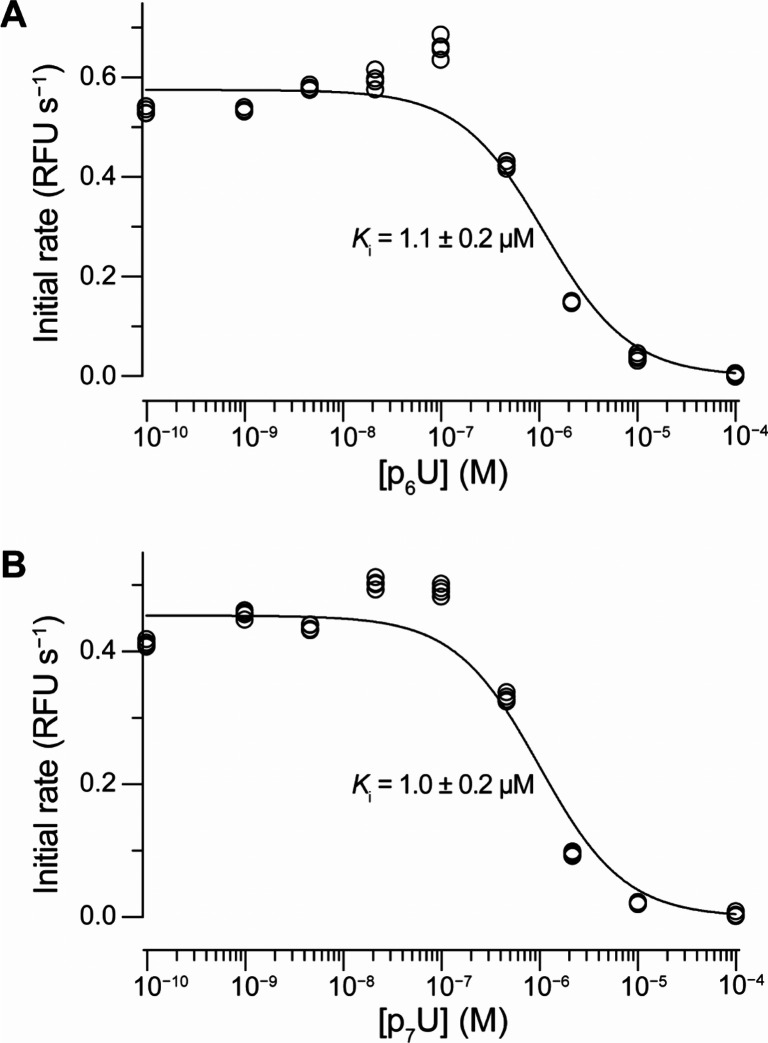
Plots showing the inhibition of the ribonucleolytic activity of RNase A by (A) **p**_**6**_**U**, and (B) **p**_**7**_**U**. RNase A (40 pM) and an oligophosphate were coincubated at room temperature for 15 min; ribonucleolytic activity was then assessed using the fluorogenic substrate 6-FAM-dArU(dA)_2_-6-TAMRA (80 nM) in 50 mM of oligo(vinylsulfonic acid)-free 50 mM MES-NaOH buffer (pH 6.0) containing NaCl (100 mM). Initial rates were determined from data accumulated over 25 min. These measurements were assayed in *n* = 4 separate reaction wells.

For comparison with the results obtained using the ^31^P{^1^H} NMR titration methodology, we have also conducted inhibition assays on RNase A using a FRET-tagged, chimeric oligonucleotide substrate.^27,47^ Here, **p**_**6**_**U** with a *K*_*i*_ value of 1.1 μM was not the strongest inhibitor, and the *K*_*i*_ value of **p**_**7**_**U** (1.0 μM) was within error of that of **p**_**6**_**U** (Figure 5 and Table 1). This assay suggests that **p**_**5**_**U**, with a *K*_*i*_ of 0.068 μM, is the strongest inhibitor among 5′-uridine nucleotides, demonstrating that longer oligophosphate chains do not automatically translate into increased RNase A inhibition activity. We suspect that the observed disparity between the results from ^31^P{^1^H} NMR titration and from the RNase A inhibition assay exists because the binding constants as measured by NMR spectroscopy are calculated directly by measuring the changes in chemical shift of one of the phosphorus signals of our ligands without determining whether these binding events specifically happen in the enzymic active site. Since RNase A is a highly cationic protein, the binding constants obtained by simply monitoring the change in phosphorus signals of our anionic ligands by ^31^P{^1^H} NMR spectroscopy may indicate a holistic binding affinity between the protein and ligand, not a protein–ligand binding affinity specific to the active site. Although our NMR titration method is possibly useful as an indication of general protein–ligand binding, the results do not directly translate as indicators of catalysis inhibition.

**Figure 6:**
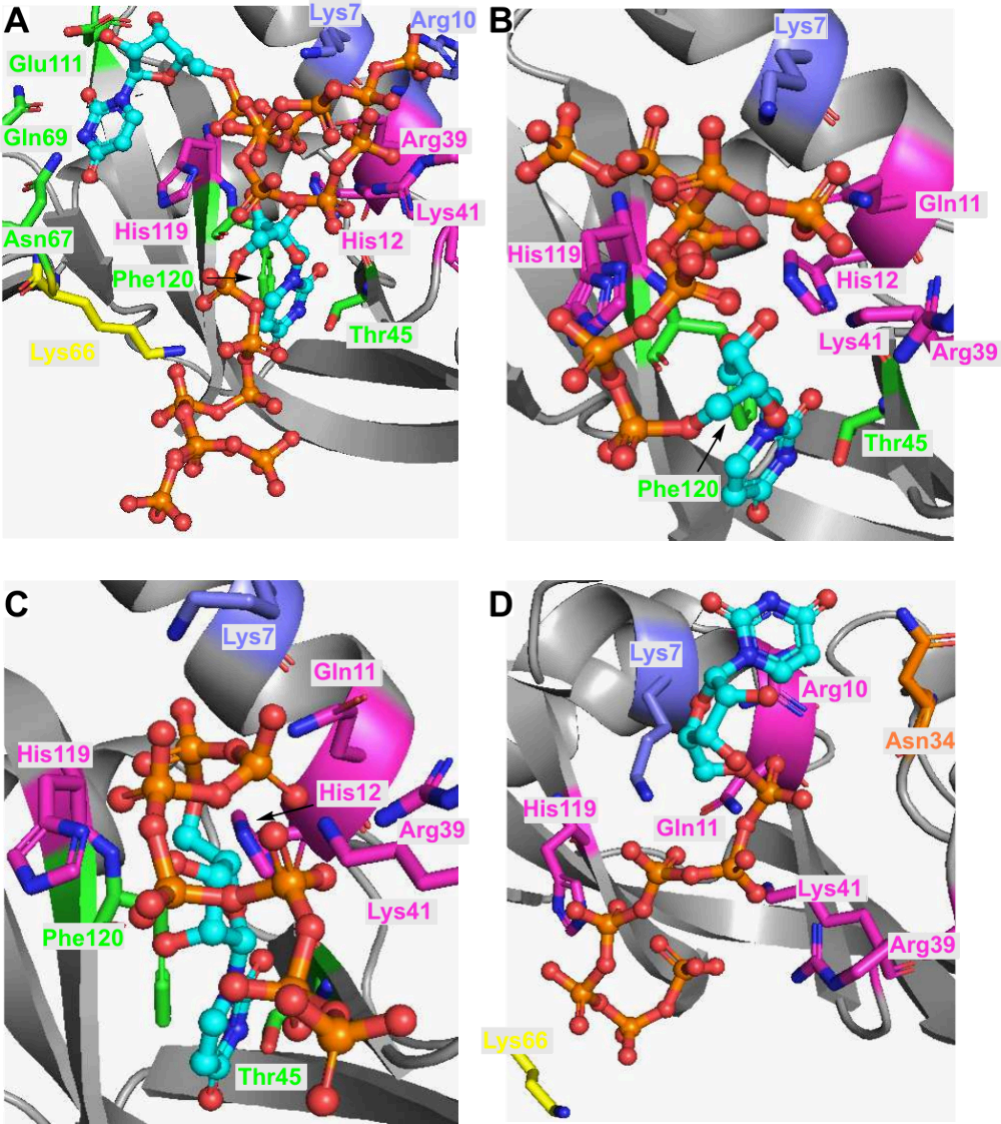
Top: Interactions between **p**_**6**_**U** and the active site of RNase A in a cocrystal structure of RNase A-**p**_**6**_**U** in chain A (A) and chain B (B). Bottom: Interactions between **p**_**7**_**U** and the active site of RNase A in a cocrystal structure of RNase A-**p**_**7**_**U** in chain A (C) and chain B (D). Residues in subsites are colored purple (P_2_ subsite), pink (P_1_ subsite), yellow (P_0_ subsite), green (B_1_ and B_2_ subsite), orange (undefined subsite).

To further understand the effect of increasing the phosphate chain length on 5′-uridine nucleotides, we set out to investigate X-ray cocrystal structures of our oligophosphate ligands coordinated to RNase A. Using a similar soaking method to that previously described,^27^ we acquired high-quality crystal structures of **p**_**6**_**U**·RNase A and **p**_**7**_**U**·RNase A (Figure 6). Like the previous structures of **p**_**2**_**U** and **p**_**5**_**U**, chain A of the structure of **p**_**6**_**U** bound to RNase A showed occupancy of both the P_0_/B_1_ and P_1_/B_2_ subsites by two ligands (Figure 6A).^27,43^ In chain B, only a single **p**_**6**_**U** binds to the P_1_/B_1_ subsite, which corresponds to the binding mode of **p**_**5**_**U** in chain B (Figure 6B). The oligophosphates of **p**_**6**_**U** in both chains reached well to the P_1_ and P_2_ subsites, affording efficient interactions with cationic residues in the active site in the solid state.

However, the oligophosphate tails in **p**_**6**_**U** are more loop-shaped in chain A and B than was found for **p**_**5**_**U**. This structural feature is expected to result in a thermodynamic penalty working in opposition to the enhanced Coulombic attraction between **p**_**6**_**U** and RNase A, leading to weaker RNase A inhibition (*K*_*i*_ (**p**_**6**_**U**) = 1.1 μM and *K*_*i*_ (**p**_**5**_**U**) = 0.068 μM). On the other hand, **p**_**7**_**U** has different positions in both chains from **p**_**2**_**U**, **p**_**5**_**U**, and **p**_**6**_**U**. A single **p**_**7**_**U** is in the active site, binding to the P_1_/B_1_ and P_2_ subsites in chain A. Interestingly, a single **p**_**7**_**U** binds only to the P_1_ and P_2_ subsites, not the B_1_ or B_2_ subsites (Figure 6C and Figure 6D). To our knowledge, this is the first example of a structure for which the binding of a pyrimidine nucleobase is not conserved within the RNase A active site. Evidently, the longer oligophosphate chain in **p**_**7**_**U** leads to this unusual nucleotide geometry, possibly translating into the weaker interaction of **p**_**7**_**U** with RNase A as compared with **p**_**5**_**U**.

Although we confirmed that 5′-adenosine nucleotides reached the maximum point of inhibition at **p**_**3**_**A** in our previous report,^27^ we also decided to study the interaction of newly prepared **p**_**6**_**A** and **p**_**7**_**A** with RNase A to determine if **p**_**3**_**A** is indeed the most powerful inhibitor among adenosine nucleotides using our employed experimental methods. RNase A inhibition constants for **p**_**6**_**A** and **p**_**7**_**A** were 3.6 μM and 2.6 μM, respectively (see Table 1). The weaker inhibition of **p**_**6**_**A** and **p**_**7**_**A** compared to that of **p**_**3**_**A** implies that the thermodynamic penalty conveyed by the increased phosphate chain length in the active site surpasses the greater Coulombic attraction between RNase A and the increasingly anionic hexa- and heptaphosphates. The cocrystal structures of RNase A and **p**_**6**_**A** or **p**_**7**_**A** confirmed that the binding sites of these nucleobases and oligophosphates are conserved (see SI), indicating that the decreased inhibition capability is due to the thermodynamic penalty.

### Computational Studies of 5′-Nucleoside Oligophosphate-RNase A Interactions

**Figure 7:**
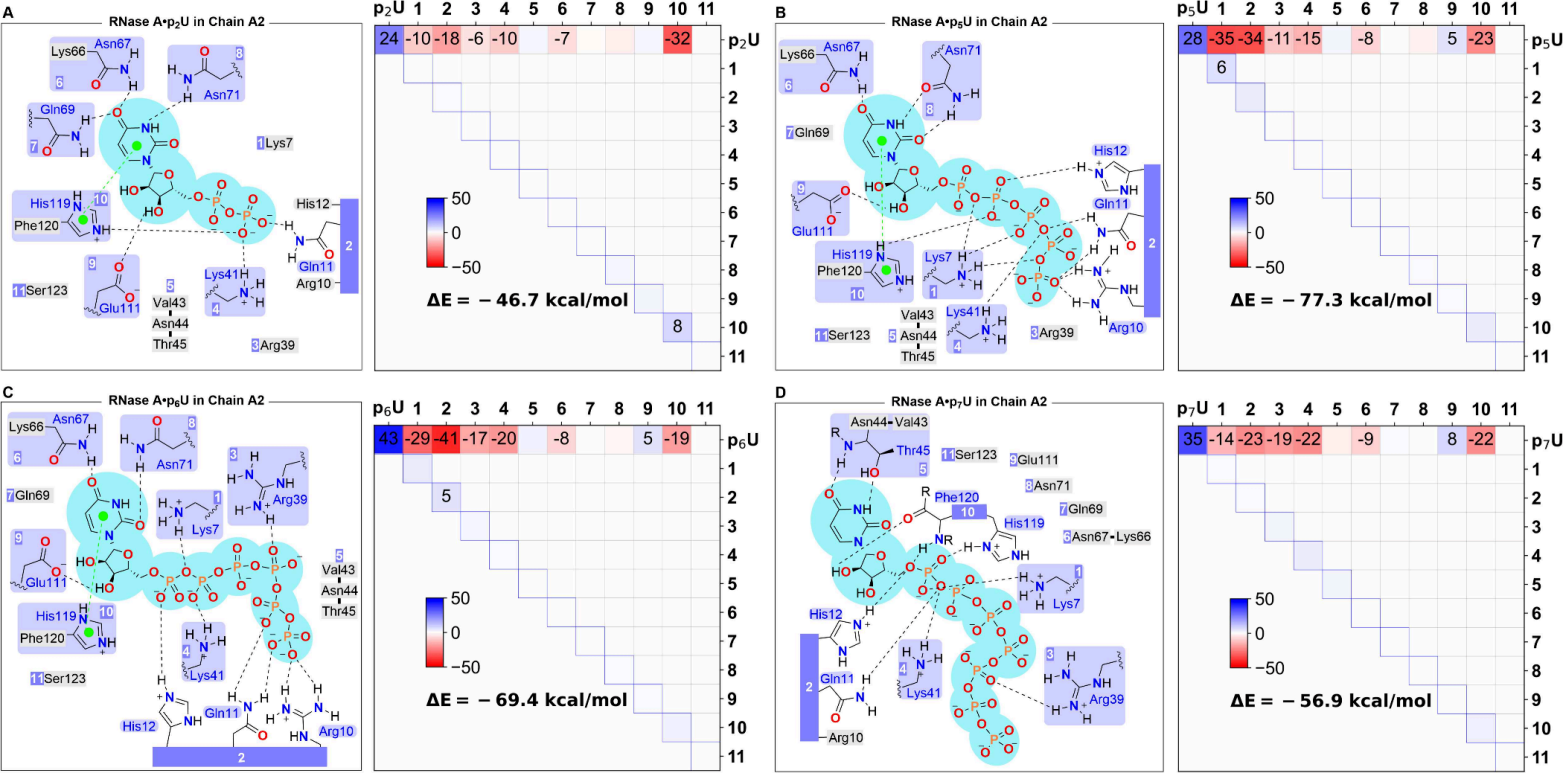
Two dimensional diagrams and LED interaction maps of the short contacts (below 3.5 Å) between RNase A and **p**_**2**_**U** (A), **p**_**5**_**U** (B), **p**_**6**_**U** (C), and **p**_**7**_**U** (D). The number in the purple-colored box corresponds to that in the LED maps (**1**=Lys 7, **2**=Glu11+His12+Arg10, **3**=Arg39, **4**=Lys41, **5**=Val43+Asn44+Thr45, **6**=Asn67+Lys66, **7**=Gln69, **8**=Asn71, **9**=Glu111, **10**=His119+Phe120, **11**=Ser123). Residues depicted in pale purple boxes exhibit close P-L contacts. Residues shown in the gray boxes exhibit no close contact. In the LED maps, repulsive (positive) interactions are highlighted in blue and attractive (negative) interactions in red. The diagonal elements represent the change in energy of the ligand and of the residues upon protein-ligand binding, respectively, originating primarily from polarization effects. The nondiagonal elements in the ligand row represent the interaction of the ligand with the residues in the active site. The remaining terms represent the change in residue–residue interactions upon ligand binding.

To gain insights into the key noncovalent interactions operating in these systems, we carried out an in-depth computational analysis. In particular, the Local Energy Decomposition (LED) scheme^48,49^ at the domain-based pair natural orbital coupled cluster DLPNO–CCSD(T) level^50^ was used to quantify and analyze the complex pattern of noncovalent interactions associated with ligand binding. All the calculations described in the following were carried out using a development version of the ORCA code based on version 5.0.^51^ As mentioned above, uridine-based ligands can bind to different enzymic sites. To identify the site showing the strongest interaction between the protein (P) and the ligand (L), preliminary P-L binding energy calculations were performed at the HF-3c^52^ level of theory for all the sites experimentally detected. These calculations were carried out directly on the experimental crystal structures (containing about 2000 atoms) using a supramolecular approach. The hydrogen positions were optimized using the GFN1-xTB method.^53^ Solvation effects were included at the C-PCM(water) level.^54,55^ These calculations revealed that all ligands preferentially bind to RNase A in chain A. Specifically, **p**_**2**_**U** and **p**_**5**_**U** preferentially bind to RNase A in chain A2, while **p**_**6**_**U** in chain A1. A graphical representation of the resulting binding modes is shown in Figure 7.

For each binding mode, the binding energy between the protein and the ligand was quantified and analyzed using the DLPNO–CCSD(T)/LED scheme. Due to the system size, accurate DLPNO–CCSD(T)/LED calculations on the entire protein–ligand adduct are computationally not feasible. Hence, to model these complex systems, we relied on a two-layer ONIOM method.^30^ In this approach, the entire protein–ligand system is divided into two subsystems: the model subsystem, containing the ligand and the key residues in the binding site, and the environment. The model subsystem was treated at the DLPNO–CCSD(T)/def2-TZVP level of theory, while the environment was modeled at the HF-3c level. The model subsystem contains the same residues for all ligands, namely Lys7, Arg10, Glu11, His12, Arg39, Lys41, Val43, Asn44, Thr45, Lys66, Asn67, Gln69, Asn71, Glu111, His119, Phe120, and Ser123. Those are the residues displaying short contacts with at least one ligand. The energy of the full system was computed using a subtractive scheme, as detailed in the SI. Electronic embedding was used to model the electrostatic interaction between the model system and the environment, while solvation effects were incorporated implicitly at the C-PCM(water) level.

It is worth mentioning here that contributions to the affinity of a ligand toward a protein include thermal corrections, entropy, and deformation free energy effects. The latter originate from structural rearrangements in the ligand and protein upon the binding process. Clearly, the precise modeling of all of these contributions falls beyond the scope of the present work. In the present case, our primary objective is to gain insights into the noncovalent interactions responsible for ligand binding by decomposing Δ*E* into a series of additive contributions corresponding to individual residue–residue and ligand-residue interactions. This can be achieved using the LED analysis in its extension for multiple fragments:^56,57^
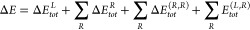
1Here, Δ*E*_*tot*_^*L*^ and Δ*E*_*tot*_^*R*^ represent the change in energy of the ligand and of the residues upon protein-ligand binding, respectively. Δ*E*_*tot*_^(*R*,*R*)^ represents the change in residue–residue interactions upon ligand binding, i.e., it provides a measure of cooperative effects between ligand-residue and residue–residue interactions. Finally, *E*_*tot*_^(*L*,*R*)^ denotes the interaction of the ligand with the residues in the active site. Additional details on how the LED scheme was extended to the ONIOM approach are given in the SI.

A graphical representation of the LED terms in eq 1 is provided by LED interaction maps,^56^ which are shown in Figure 7 for the 5′-uridine nucleotides **p**_**2**_**U**, **p**_**5**_**U**, **p**_**6**_**U**, and **p**_**7**_**U**. The LED maps reveal that, upon the binding of the ligand to RNase A, a new network of intermolecular interactions is created between the ligand and the residues in the active site. The strength of these interactions is quantified by the corresponding *E*_*tot*_^(*L*,*R*)^ terms, which are displayed in the first row of each map (the ligand row). The ligand-residue interactions perturb the electronic structure of the ligand and of the residues, increasing their electronic energy by Δ*E*_*tot*_^*L*^ and Δ*E*_*tot*_^*R*^, respectively. These repulsive contributions to the binding energy, accounting for so-called Pauli repulsion effects, are displayed in the diagonal elements of the LED maps. To simplify the discussion, the energy associated with the geometric strain of the ligand resulting from binding was also included in Δ*E*_*tot*_^*L*^. Hence, this term incorporates the energy penalty associated with both electronic and geometric preparation effects.^48,49^ Finally, the remaining nondiagonal elements of the map (corresponding to the *E*_*tot*_^(*R*,*R*)^ terms) show the related changes in the interactions between the residues. These interactions are essentially negligible, indicating that ligand binding has a weak effect on the strength of residue–residue interactions.

Our analysis reveals that increasing the phosphate chain length beyond a certain point results in diminishing returns in P-L binding energies, consistent with the experimental trends of inhibition constants discussed above. The maximum binding energy was obtained with **p**_**5**_**U**. Interestingly, all ligands show a strong electrostatic interaction with His119, while the interaction with the other charged residues differs significantly for different ligands. In particular, both **p**_**5**_**U** and **p**_**6**_**U** show strong and stabilizing electrostatic interactions with Lys7 and with many of the other charged residues. Overall, these electrostatic interactions are slightly larger for **p**_**6**_**U** than for **p**_**5**_**U**. However, this effect is fully counteracted by a significant increase in the ligand strain energy for **p**_**6**_**U**, which is likely associated with the peculiar “loop-like” conformation of the phosphate tail mentioned above. Interestingly, increasing the phosphate chain length also leads to a small but consistent increase in the Coulombic repulsion with Glu111, which also contributes, to a smaller extent, to reducing the binding energy for longer ligand backbones.

As for the 5′-adenosine hexa- and heptaphosphates, the computational results support our observation of the cocrystal structures with RNase A, showing that all 5′-adenosine nucleotides display similar electronic interactions with the residues in the active site of RNase A (see SI). While an increasing electrostatic interaction is indeed observed for the longer phosphate chains, this increase is counterbalanced to a large extent by a corresponding increase in the ligand strain energy. These results suggest that other thermodynamic effects, such as conformational entropy changes upon binding, play a crucial role in determining the binding constant trend experimentally observed for this class of ligands. The development of computational protocols aimed at accurately describing these and other dynamic effects is beyond the scope of the present work. Overall, our results confirm that adenosine nucleotides with longer phosphate chains than **p**_**3**_**A** adopt a convoluted conformation and are associated with a weaker binding ability toward RNase A in the active site.

## Summary and Concluding Remarks

With this work, we took a journey beginning with fundamental inorganic synthesis. Conditions were found that permitted expansion of the cyclic metaphosphate tetramer into its corresponding pentamer, the first selective synthesis of pentametaphosphate. The latter could be converted into its anhydride, which we envisioned as a potential reagent for attachment of a pentaphosphate chain to biomolecules in an era when the realization is dawning that mono, di, and triphosphates are far from the limit. While the anhydride of dihydrogen pentametaphosphate on its own was insufficiently reactive for spontaneous attachment to nucleoside mono or diphosphates, activation was achieved with an organometallic promoter, rather than with the typical magnesium or zinc chlorides. While the yields of targeted nucleoside hexa and heptaphosphates were less than ideal, their isolation in pure form was accomplished for the first time. For comparison, previous reports described the syntheses of bis-adenosine heptaphosphate (**Ap**_**7**_**A**) and a terminal propargyl-P-amidate analogue of **p**_**6**_**A**.^17,38^

The reader may wonder whether the fundamental inorganic synthesis methodology reported herein can be extended to the development of a reagent for hexaphosphorylation; thus far our efforts in this regard have not met with success. It remains an enticing target for future work.

It being the case that the new conjugates are polyanionic biorelevant constructs, we undertook an investigation of their interactions with RNase A, a model protein with a polycationic active site. First we looked at supramolecular chemistry inspired titrations with a convenient ^31^P NMR spectroscopic handle, but data so obtained were likely indicative of general, nonspecific binding overlaid with interactions in the enzyme active site. Next, we used a turn-on fluorescence assay to obtain inhibition constants for the new constructs, none of which were found to be more potent than the previously investigated uridine pentaphosphate. In order to understand these results we turned to a combined X-ray crystallographic study and computational analysis, the latter providing insight into the energy of active site interactions on a residue by residue basis. An unexpected finding was the unconserved hydrogen bonding site for uridine in its heptaphosphate. The computational study revealed that a “loop-like” conformation for the hexaphosphate of uridine was likely responsible for its diminished binding energy as compared to the more potent pentaphosphate. Going forward, we plan on improving our synthetic access to nucleoside oligophosphates, and on expanding the range of biomolecules, including polyphosphate kinases (PPKs), to which oligophosphates can be conjugated using our reagents and methods.

## Supporting Information Available

Experimental details and characterization data (PDF)

## Accession Codes

Structure data for new compounds are available from the Protein Database under the following PDB codes: RNase A-**p**_**6**_**A**, 8GGG; RNase A-**p**_**6**_**U**, 8FHM; RNase A-**p**_**7**_**A**, 8S96; RNase A-**p**_**7**_**U**, 8GC9.
