# Supplementary material for: Pentaphosphorylation via the Anhydride of Dihydrogen Pentametaphosphate: Access to Nucleoside Hexa- and Heptaphosphates and Study of Their Interaction with Ribonuclease A
Source: ACS Cent Sci. 2024 Jul 15;10(7):1415–22. doi: 10.1021/acscentsci.4c00835 (PMC11273453; doi:10.1021/acscentsci.4c00835)
Supplement: Supplementary file 1 — oc4c00835_si_001.pdf [file oc4c00835_si_001.pdf]

Supporting Information:

Pentaphosphorylation via the Anhydride of Dihydrogen Pentametaphosphate: Access to Nucleoside Hexa- and Heptaphosphates and Study of Their Interaction with Ribonuclease A

Gyeongjin Park,† Evans C. Wralstad,† Noelia Faginas-Lago,‡ Kevin Qian,† Ronald T. Raines,*,† Giovanni Bistoni,*,‡ and Christopher C. Cummins*,†

†*Department of Chemistry, Massachusetts Institute of Technology, Cambridge, Massachusetts, 02139, United States*

‡*Department of Chemistry, Biology,and Biotechnology, University of Perugia, 06123, Perugia, Italy*

E-mail: rtraines@mit.edu; giovanni.bistoni@unipg.it; ccummins@mit.edu

Contents

S1 General considerations **S3**

S2 Synthesis and Characterization of Compounds **S4**

S2.1 Synthesis of $[PPN]_2[TBA]$**1** S4

S2.2 Synthesis of $[PPN]_2[TBA]$**2** S5

S2.3 Synthesis of $[NH_4]_6[HP_6A]$ S6

S2.4 Synthesis of $[NH_4]_6[HP_6U]$ S7

S2.5 Synthesis of $[NH_4]_7[HP_7A]$ S8

S2.6 Synthesis of $[NH_4]_7[HP_7U]$ S9

S3 RNase A Binding Experiments **S43**

S3.1 Direct titration method S43

S3.2 Indirect titration method S46

S4 RNase A Inhibition Kinetics **S55**

S5 X-ray Protein Crystallography **S60**

S6 Computational Details **S62**

S6.1 HF-3c Binding Energy Calculations S62

S6.2 ONIOM Binding Energy Calculations S62

S6.3 Geometry Optimizations and Ligand Strain Energies S65

S6.4 Local Energy Decomposition analysis and LED Maps S67

S6.5 Computational Studies on Adenosine Derivatives S68

S7 References **S72**

S1 General considerations

All manipulations were carried out in a Vacuum Atmospheres model MO-40M glovebox under an inert atmosphere of purified nitrogen unless otherwise noted. The anhydrous tetrabutylammonium (TBA) salts of 5'-UMP, 5'-AMP, and 5'-ADP were prepared according to the procedure of Taylor.[S1] All solvents used in the glovebox were obtained anhydrous and oxygen-free by bubble by the method of Grubbs,[S2] and stored over activated 4A sieves. HPLC grade water was purchased from Sigma Aldrich and used as received. $[PPN]_4[P_4O_{12}]\cdot 5H_2O$ and $[NH_4]_5[HP_5U]$ were prepared according to the literature procedure.[S3,S4] All other reagents were purchased and used as received. Deuterated solvents were purchased from Cambridge Isotope Labs and used as received. NMR spectra were obtained at ambient temperature (ca. 25 °C) on a Bruker Avance 400 or 500 instruments. ^1H and ^{13}C NMR spectra were referenced to residual solvent peaks: D_2O (^1H = 4.79 ppm), $CDCl_3$ (^1H = 7.26 ppm, ^{13}C = 77.16 ppm), CD_3CN (^1H = 1.94 ppm, ^{13}C = 1.32, 118.26 ppm). ^{31}P NMR spectra were referenced externally to 85% H_3PO_4 (0 ppm). Electrospray ionization mass spectra (ESI- MS(-)) were acquired on an Agilent 6545 QToF ESI spectrometer. HPLC purifications were performed using an Agilent 1260 Infinity II HPLC system comprising a 1260 Infinity II Preparative Binary Pump coupled to a 1260 Infinity II Diode Array Detector. Anion exchange HPLC utilized a PRP-X100 anion exchange column and was carried out with a gradient method from 100% A, 0% B to 0% A, 100% B (A = 100% water; B = aqueous 1M NH_4HCO_3) with a 6 mL/min flow rate. Ribonuclease A was purchased from Sigma-Aldrich (product 4875 for NMR titration and product R6513 for X-ray protein crystallization) and was used without further purification.

S2 Synthesis and Characterization of Compounds

S2.1 Synthesis of $[PPN]_2[TBA]$**1**

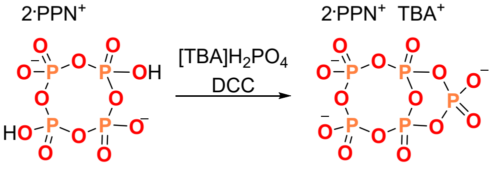

In a glovebox, *N*, *N*'- dicyclohexylcarbodiimide (DCC, 0.43 g, 2.08 mmol) was added to a solution of the mixture of $[PPN]_2[P_4O_{12}H_2]$[S3] (1.0 g, 0.72 mmol) and $[TBA]H_2PO_4$ (0.24 g, 0.72 mmol) in a 20 mL vial with 5 mL of acetonitrile at room temperature. The resulting solution was stirred for 48 hours at room temperature and then filtered in a short pad of Celite to remove insoluble dicyclohexylurea (DCU). The filtrate was dried in vacuo and purified by recrystallization from CH_3CN/Et_2O, affording a waxy white solid. The solid was rinsed three times with 15 mL of ether, then dried under vacuum, producing the product as a white solid (0.88 g, 0.52 mmol, 72% yield). ^1H NMR (400 MHz, CD_3CN) δ 7.69 – 7.64 (m, 12H), 7.62 – 7.53 (m, 24H), 7.50-7.46 (m, 24H), 3.15 – 3.08 (m, 8H), 1.61 (p, J = 7.4 Hz, 8H), 1.36 (h, J = 7.4 Hz, 8H), 0.97 (t, J = 7.4 Hz, 12H). $^{13}C\{^1H\}$ NMR (101 MHz, CD_3CN) δ 134.57 (s), 133.39 – 132.59 (m), 130.85 – 129.59 (m), 128.71 (s), 127.64 (s), 60.24 – 56.74 (m), 24.30 (s), 20.29 (s), 13.78 (s).$^{31}P\{^1H\}$ NMR (162 MHz, CD_3CN): δ 21.00 (s, 4P), -24.92 (t, J = 24.4 Hz, 1P), –25.66 – –26.02 (m, 2P), –35.87 – –36.38 (m, 2P). ESI-MS calc'd for $C_{32}H_{72}N_2O_{14}P_5{}^-[TBA]_2[P_5O_{14}]^-$ 863.3677, found 863.0684. While [PPN][H2PO4] also allows for the synthesis of $[PPN]_3$**1**, the preparation of $[PPN][H_2PO_4]$ is not that easy. In contrast, $[TBA][H_2PO_4]$ is commercially available. Furthermore, the choice of counter cations does not affect the reaction, which makes the

use of [TBA][H_2PO_4] more efficient in terms of both time and cost.

S2.2 Synthesis of $[PPN]_2[TBA]$2

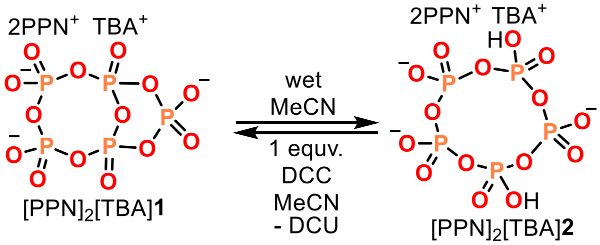

In a 20 mL vial, $[PPN]_2[TBA]P_5O_{14}$ (62 mg, 0.037 mmol) was dissolved in 1 mL of acetonitrile at room temperature, and took out of glovebox. H_2O (13.5 μL, 0.74 mmol) was added into the reaction vial. The resulting solution was stirred for 24 hours at room temperature, and was dried in vacuo, giving an oily sold. The solid material was rinsed three times with 15 mL of ether, then dried under vacumm, producing the product as a white solid (55 mg, 0.032 mmol, 88 % yield). We also confirmed that the addition of 1 equiv of DCC to the acetonitrile solution of **2** induces the formation of **1** by ^{31}P NMR spectroscopy. ^{1}H NMR (500 MHz, CD_3CN) δ 7.69 – 7.65 (m, 12H), 7.61 – 7.55 (m, 24H), 7.50-7.46 (m, 24H), 3.14 – 3.10 (m, 8H), 1.61 (p, $J = 7.4$ Hz, 8H), 1.37 (h, $J = 7.4$ Hz, 8H), 0.97 (t, $J = 7.4$ Hz, 12H). ^{13}C{^{1}H} NMR (126 MHz, CD_3CN) δ 134.56 (s), 133.27 – 132.12 (m), 130.41 – 130.23 (m), 128.60 (d, $J = 2.1$ Hz), 127.74 (d, $J = 2.2$ Hz), 59.24 – 59.19 (m), 23.29 (s), 20.27 (s), 13.79 (s).^{31}P{^{1}H} NMR (162 MHz, CH_3CN): δ 21.00 (s, 4P), -26.82 (s, 5P). ESI-MS calc'd for $C_{52}H_{68}N_2O_{15}P_7^-$ [TBA][PPN][$P_5O_{14}H_2$]$^-$ 1177.2788, found 1177.2802. ESI-MS calc'd for $C_{72}H_{62}N_2O_{15}P_9^-$ $[PPN]_2[P_5O_{14}H_2]^-$ 1473.1794, found 1473.1801.

S2.3 Synthesis of $[NH_4]_6[HP_6A]$

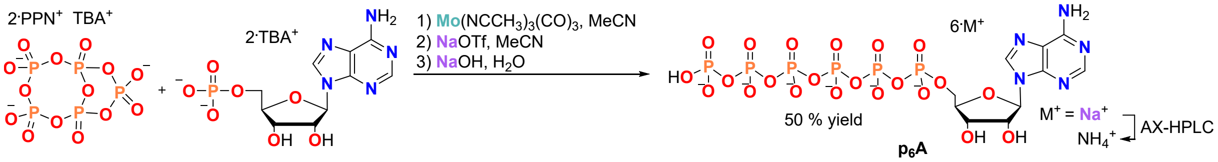

In a glovebox, $[PPN]_2[TBA]$**1** (90 mg, 0.053 mmol) and $Mo(NCCH_3)_3(CO)_3$ (32 mg, 0.11 mmol) were dissolved and stirred in acetonitrile (2 mL) for 10 min. A solution of $[TBA]_2AMP$ (43.8 mg, 0.053 mmol) in 1 mL of acetonitrile was added to the reaction mixture and stirred overnight. To the resulting solution was then added a solution of sodium triflate (0.10 g, 0.58 mmol) in 1 mL of acetonitrile. Once the precipitation was induced, all volatile materials in the reaction vessel were removed under reduced pressure, giving a tan-yellow solid. The solid material was rinsed three times with 5 mL of acetonitrile, taken out of the glove box, and then suspended in 10 mL of high-purity water. The resulting solution was filtered through a short pad of Celite and lyophilized overnight, affording a pale brown solid. The solid was redissolved in 1.5 mL HPLC grade water to give a brown solution. 1.1 mL of 1 M NaOH was added to the brown solution, stirring the mixture for 3 hours. The solution went from pale brown color to colorless. It was passed through an Acrodisc 0.2 μm wwPTFE syringe filter and purified by AX-HPLC according to the procedure in the General Considerations. The product fractions were collected and lyophilized, yielding a fluffy white solid (22 mg, 0.026 mmol, 50 % yield). ^{1}H NMR (500 MHz, D_2O) δ 8.62 (s, 1H), 8.38 (s, 1H), 6.17 (d, J = 5.7 Hz, 1H), 4.91 – 4.88 (m, 1H), 4.65 – 4.63 (m, 1H), 4.44 – 4.42 (m, 1H), 4.34 – 4.23 (m, 2H). $^{13}C\{^1H\}$ NMR (126 MHz, D_2O) δ 155.67 (s), 152.87 (s), 149.23 (s), 139.87 (s), 118.61 (s), 86.54 (s), 84.21 (s), 74.22 (s), 70.45 (s), 65.39 (d, J = 5.8 Hz). $^{31}P\{^1H\}$ NMR (203 MHz, D_2O): δ -10.78 (d, J = 17.8 Hz, 1P), -11.40 (d, J = 16.4 Hz, 1P), -22.46 – -23.17 (m, 4P).

ESI-MS calc'd for $C_{11}H_{20}N_5O_{21}P_6^-$ $[HP_6A]^-$ 745.8874, found 745.8871. ESI-MS calc'd for $C_{11}H_{19}N_5O_{21}P_6^{2-}$ $[H_5P_6A]^{2-}$ 372.4401, found 372.4401. *Note: instead of performing cation exchange from Mo to Na^+, we attempted to open the ring with amines. However, this method did not effectively open the ring. Additionally, we tried opening the ring after the cation exchange from Mo to Na^+ to potentially enhance the yield. Unfortunately, this approach resulted in a significant production of AMP and pentametaphosphate, indicating that it does not contribute to yield improvement.*

S2.4 Synthesis of $[NH_4]_6[HP_6U]$

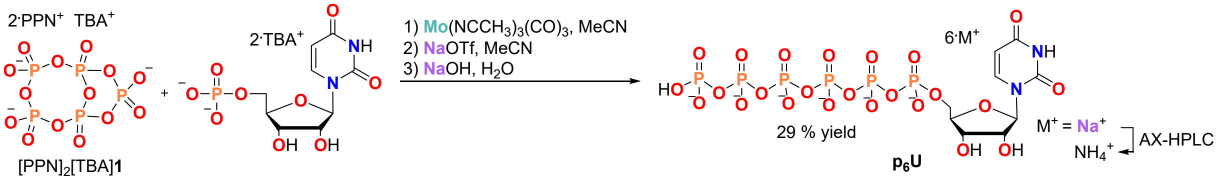

In a glovebox, $[PPN]_2[TBA]$**1** (0.18 g, 0.106 mmol) and $Mo(NCCH_3)3(CO)_3$ (64 mg, 0.22 mmol) were dissolved and stirred in acetonitrile (5 mL) for 10 min. A solution of $[TBA]_2$UMP (86 mg, 0.106 mmol) in 2 mL of acetonitrile was added to the reaction mixture and stirred overnight. To the resulting solution was then added a solution of sodium triflate (0.20 g, 1.16 mmol) in 2 mL of acetonitrile. Once the precipitation was induced, all volatile materials in the reaction vessel were removed under reduced pressure, giving a tan-yellow solid. The solid material was rinsed three times with 5 mL of acetonitrile, taken out of the glove box, and then suspended in 10 mL of high-purity water. The resulting solution was filtered through a short pad of Celite and lyophilized overnight, affording a pale brown solid. The solid was redissolved in 2 mL HPLC grade water to give a brown solution. 2.2 mL of 1 M NaOH was added to the brown solution, and the resulting mixture was stirred for 3 hours. The solution went from pale brown color to

colorless. It was passed through an Acrodisc 0.2 μm wwPTFE syringe filter and purified by AX-HPLC according to the procedure in the General Considerations. The product fractions were collected and lyophilized, yielding a fluffy white solid (25 mg, 0.030 mmol, 29 % yield). ^{1}H NMR (500 MHz, D_2O) δ 7.97 (d, J = 8.1 Hz, 1H), 6.02 (d, J = 5.5 Hz, 1H), 5.99 (d, J = 8.1 Hz, 1H), 4.46 – 4.40 (m, 2H), 4.32 – 4.30 (m, 1H), 4.27 – 4.25 (m, 2H). ^{13}C{^{1}H} NMR (126 MHz, D_2O) δ 166.22 (s), 151.90 (s), 141.66 (s), 102.73 (s), 87.93 (s), 83.60 (d, J = 9.0 Hz), 73.64 (s), 69.78 (s), 65.16 (d,J = 5.7 Hz). ^{31}P{^{1}H} NMR (203 MHz, D_2O): δ -6.25 (d, J = 19.5 Hz, 1P), -11.53 (d, J = 18.0 Hz, 1P), -21.53 – -21.97 (m, 1P), -22.44 – -22.59 (m, 1P), -22.86 – -23.26 (m, 2P). ESI-MS calc'd for $C_9H_{17}N_2O_{24}P_6^-$ $[H_6P_6U]^-$ 722.8602, found 722.8613. ESI-MS calc'd for $C_9H_{16}N_2O_{24}P_6^{2-}$ $[HP_6U]^{2-}$ 360.9265, found 360.9271.

S2.5 Synthesis of $[NH_4]_7[HP_7A]$

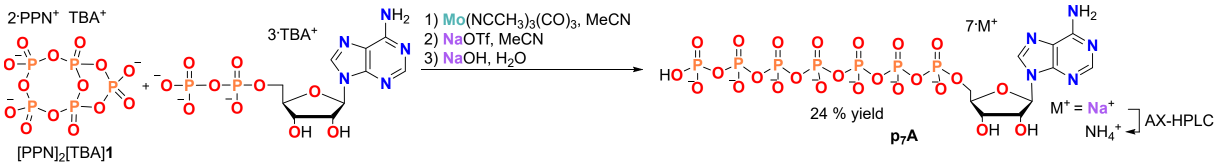

In a glovebox, $[PPN]_2[TBA]$**1** (90 mg, 0.053 mmol) and $Mo(NCCH_3)_3(CO)_3$ (64 mg, 0.22 mmol) were dissolved and stirred in acetonitrile (2 mL) for 10 min. A solution of $[TBA]_3$ADP (61 mg, 0.053 mmol) in 1 mL of acetonitrile was added to the reaction mixture and stirred for two days. To the resulting solution was then added a solution of sodium triflate (0.10 g, 0.58 mmol) in 2 mL of acetonitrile. Once the precipitation was induced, all volatile materials in the reaction vessel were removed under reduced pressure, giving a tan-yellow solid. The solid material was rinsed three times with 5 mL of acetonitrile, taken out of the glove box, and then suspended in 10 mL of high-purity water. The

resulting solution was filtered through a short pad of Celite and lyophilized overnight, affording a pale brown solid. The solid was redissolved in 3 mL HPLC grade water to give a brown solution. 1.1 mL of 1 M NaOH was added to the brown solution, stirring the mixture for 3 hours. The solution went from pale brown color to colorless. It was passed through an Acrodisc 0.2 μm wwPTFE syringe filter and purified by AX-HPLC according to the procedure in the General Considerations. The product fractions were collected and lyophilized, yielding a fluffy white solid (12 mg, 0.013 mmol, 24 % yield). ^{1}H NMR (500 MHz, D_2O) δ 8.58 (s, 1H), 8.33 (s, 1H), 6.16 (d, J = 6.0 Hz, 1H), 4.88 – 4.84 (m, 1H), 4.62 – 4.60 (m, 1H), 4.44 – 4.42 (m, 1H), 4.33 – 4.22 (m, 2H). ^{13}C{^{1}H} NMR (101 MHz, D_2O) δ 151.91 (s), 148.60 (s), 147.76 (s), 141.80 (s), 118.44 (s), 87.81 (s), 84.43 (d, J = 9.09 Hz), 74.32 (s), 70.44 (s), 65.27 (d, J = 5.8 Hz). ^{31}P{^{1}H} NMR (203 MHz, D_2O): δ -5.98 (br, 1P), -11.45 – -11.36 (d, J = 15.3 Hz, 1P), -21.34 – -21.71 (m, 1P), -22.10 – -22.42 (m, 1P), -22.64 – -23.10 (m, 2P). ESI-MS calc'd for $C_{10}H_{19}N_5O_{25}P_7^{-}$ $[H_7P_7A]^{-}$ 825.8537, found 825.8570. ESI-MS calc'd for $C_{10}H_{18}N_5O_{25}P_7^{2-}$ $[HP_7U]^{2-}$ 412.4232, found 412.4249.

S2.6 Synthesis of $[NH_4]_7[HP_7U]$

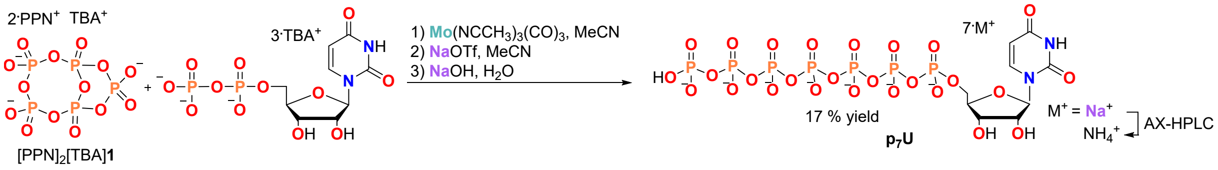

In a glovebox, $[PPN]_2[TBA]$**1** (90 mg, 0.053 mmol) and $Mo(NCCH_3)_3(CO)_3$ (64 mg, 0.22 mmol) were dissolved and stirred in acetonitrile (2 mL) for 10 min. A solution of $[TBA]_3$UDP (60 mg, 0.053 mmol) in 1 mL of acetonitrile was added to the reaction mixture and stirred for two days. To the resulting solution was then added a solution

of sodium triflate (0.10 g, 0.58 mmol) in 2 mL of acetonitrile. Once the precipitation was induced, all volatile materials in the reaction vessel were removed under reduced pressure, giving a tan-yellow solid. The solid material was rinsed three times with 5 mL of acetonitrile, taken out of the glove box, and then suspended in 10 mL of high-purity water. The resulting solution was filtered through a short pad of Celite and lyophilized overnight, affording a pale brown solid. The solid was redissolved in 3 mL HPLC grade water to give a brown solution. 1.1 mL of 1 M NaOH was added to the brown solution, stirring the mixture for 3 hours. The solution went from pale brown color to colorless. It was passed through an Acrodisc 0.2 μm wwPTFE syringe filter and purified by AX-HPLC according to the procedure in the General Considerations. The product fractions were collected and lyophilized, yielding a fluffy white solid (8.3 mg, 0.009 mmol, 17 % yield). ^{1}H NMR (400 MHz, D_2O) δ 7.97 (d, J = 8.2 Hz, 1H), 6.03 – 5.96 (m, 2H), 4.45 – 4.38 (m, 2H), 4.32 – 4.23 (m, 3H). ^{13}C{^{1}H} NMR (126 MHz, D_2O) δ 166.19 (s), 151.85 (s), 141.66 (s), 102.71 (s), 88.03 (s), 83.50 (s), 73.74 (s), 69.70 (s), 65.07 (d, J = 5.01 Hz). ^{31}P{^{1}H} NMR (203 MHz, D_2O): δ -6.37 (d, J = 18.7 Hz, 1P), -11.60 (br, 1P), -21.63 – -21.83 (m, 1P), -22.44 – -22.58 (m, 1P), -22.82 – -23.14 (m, 2P). ESI-MS calc'd for $C_9H_{18}N_2O_{27}P_7^-$ $[H_7P_7U]^-$ 802.8265, found 802.8271. ESI-MS calc'd for $C_9H_{17}N_2O_{27}P_7^{2-}$ $[HP_7U]^{2-}$ 400.9096, found 400.9102.

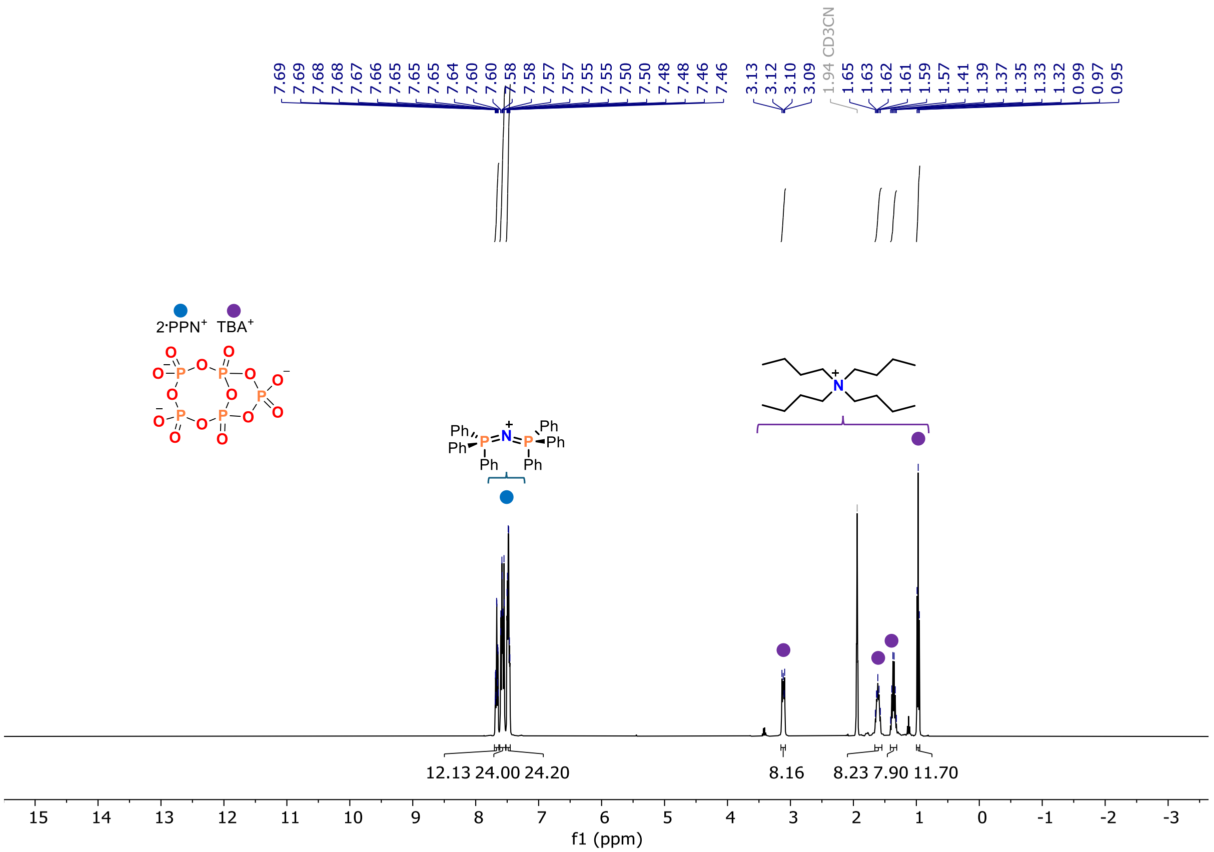

Figure S1: ^{1}H NMR spectrum of $[PPN]_2[TBA]\mathbf{1}$ in CD_3CN.

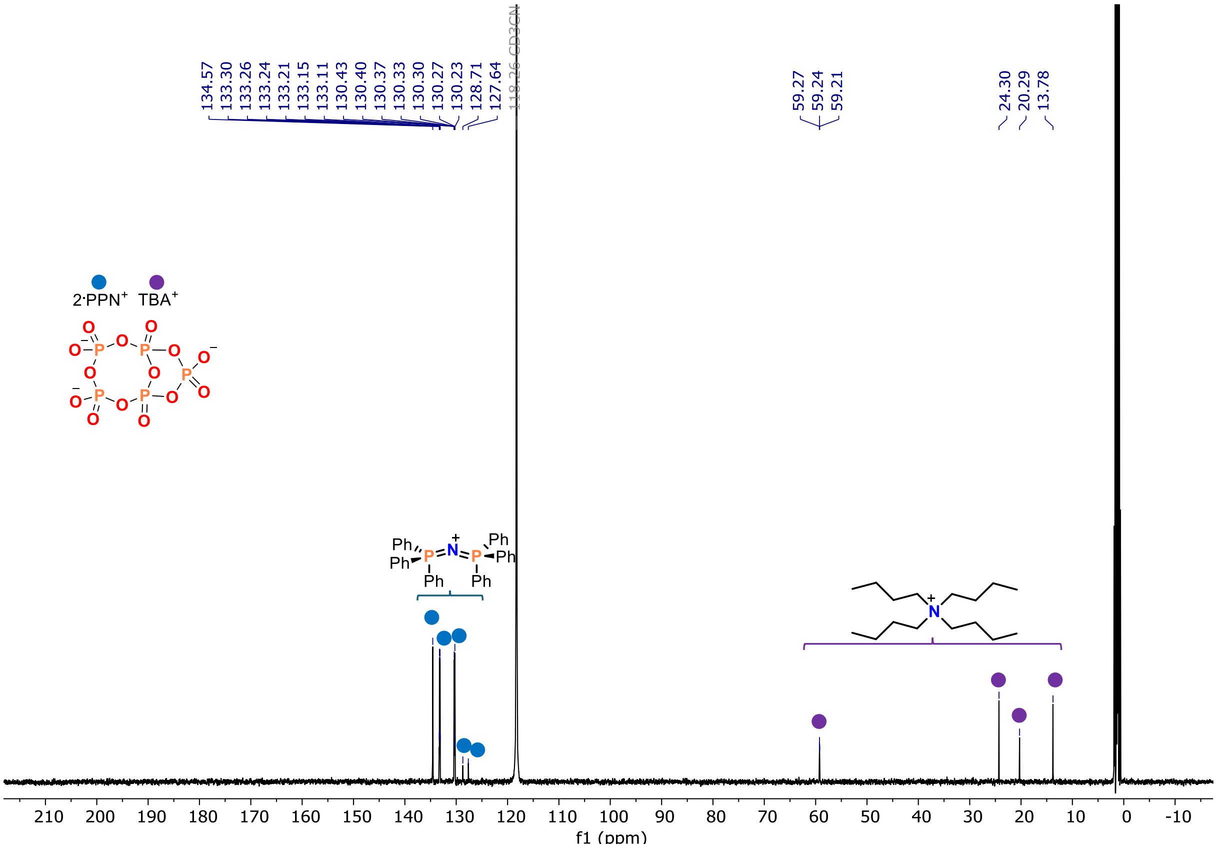

Figure S2: $^{13}C\{^{1}H\}$ NMR spectrum of $[PPN]_2[TBA]$**1** in CD_3CN

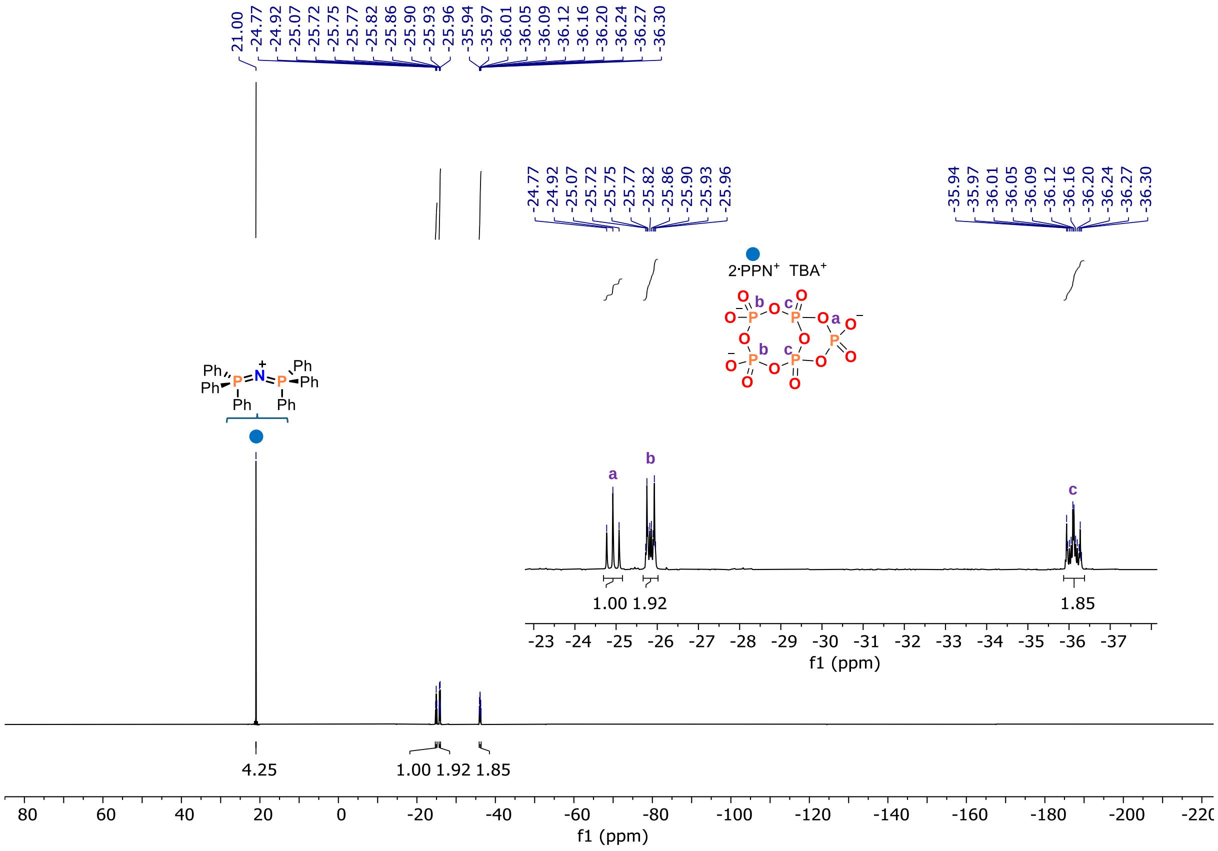

Figure S3: ^{31}P{^{1}H} NMR spectrum of $[PPN]_2[TBA]$**1** in CD_3CN.

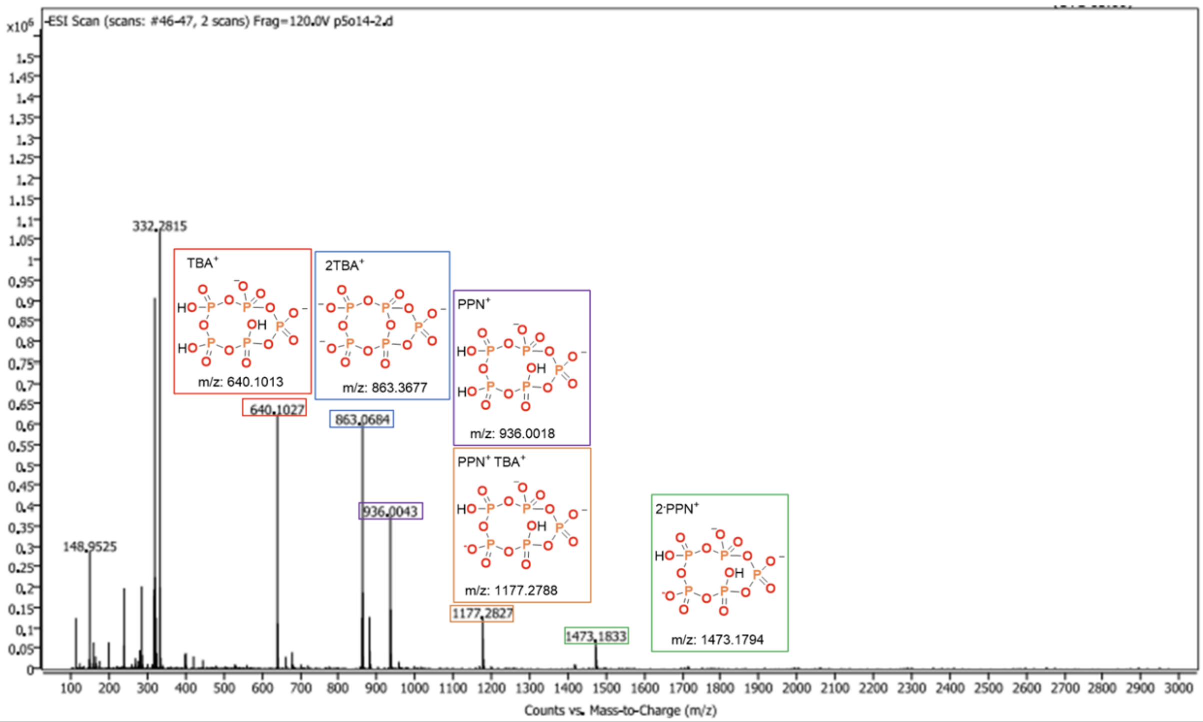

Figure S4: ESI-MS(–) of $[PPN]_2[TBA]$**1** (acetonitrile, 25 μg/mL) with assigned structures showing calculated m/z values.

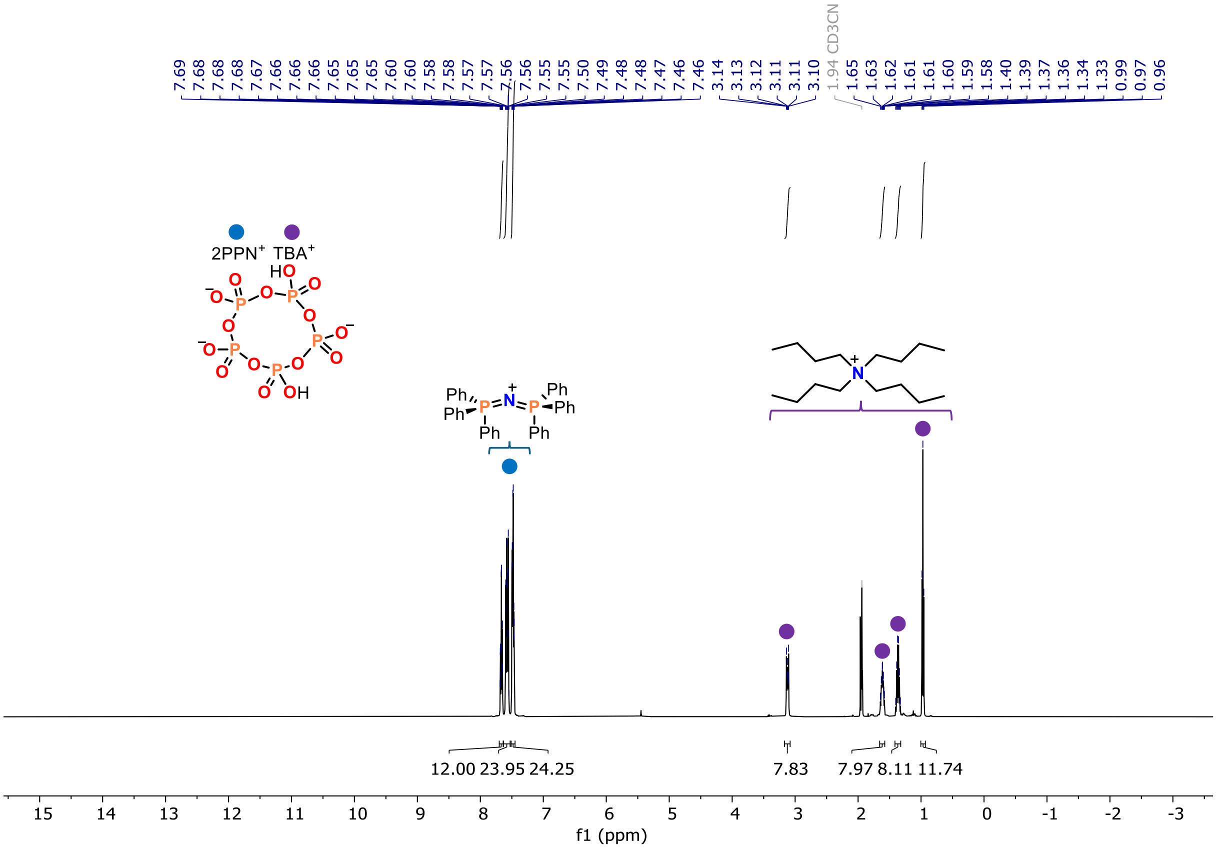

Figure S5: ^{1}H NMR spectrum of $[PPN]_2[TBA]$**2** in CD_3CN.

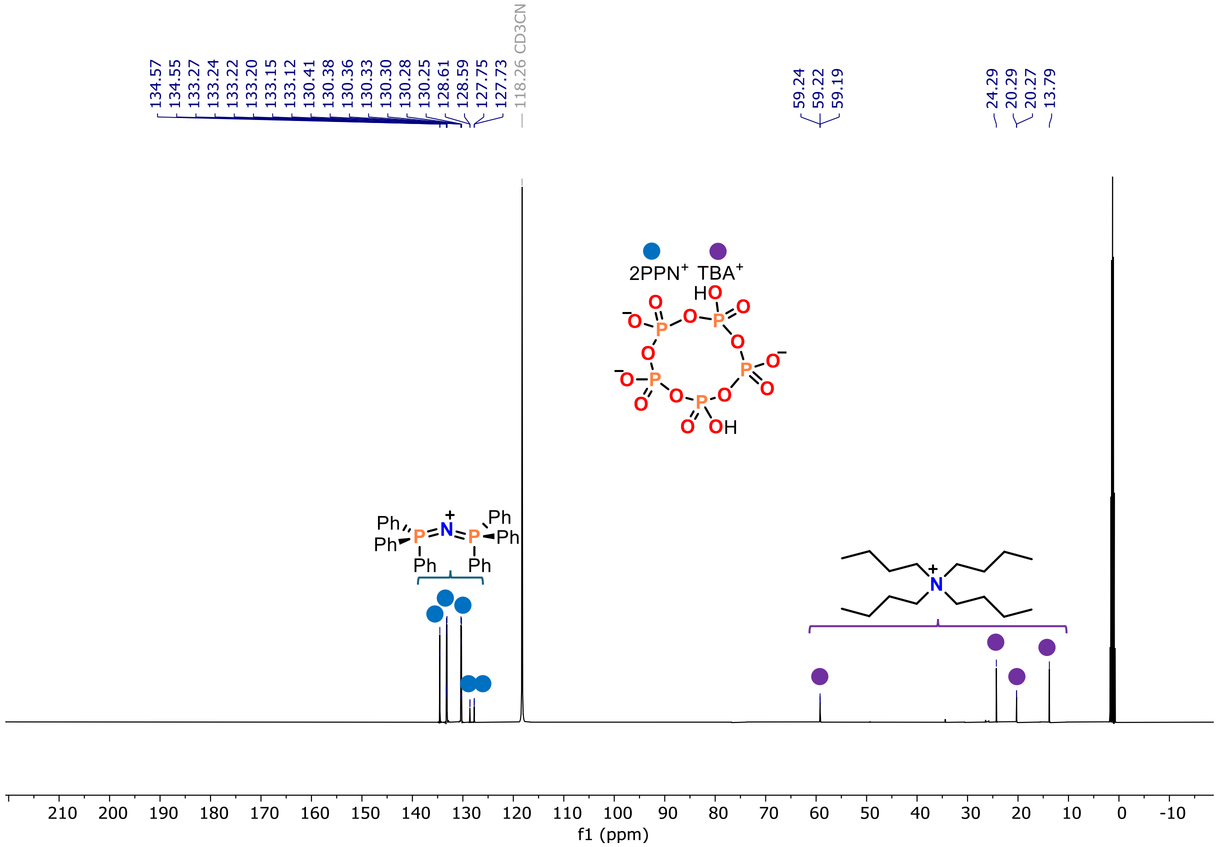

Figure S6: $^{13}C\{^{1}H\}$ NMR spectrum of $[PPN]_2[TBA]$**2** in CD_3CN.

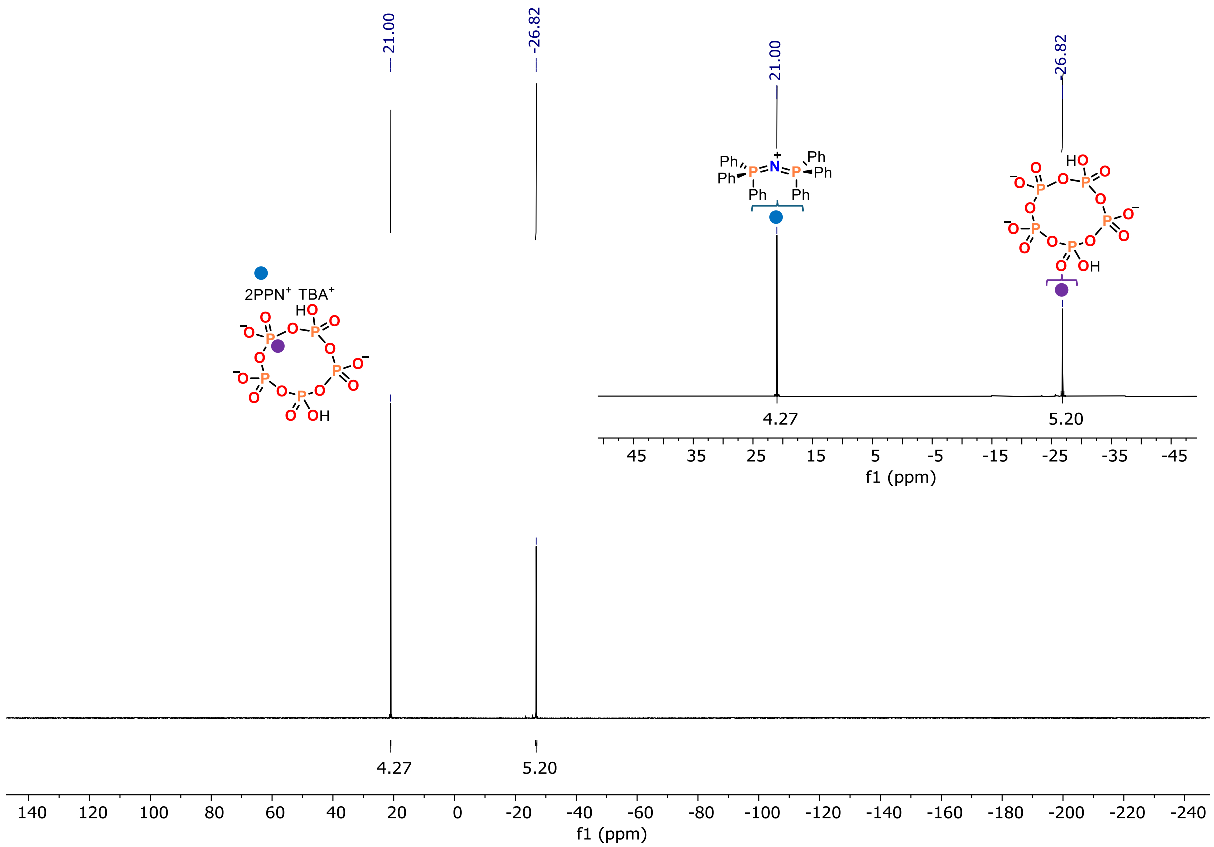

Figure S7: $^{31}P\{^{1}H\}$ NMR spectrum of $[PPN]_2[TBA]$**2** in CH_3CN.

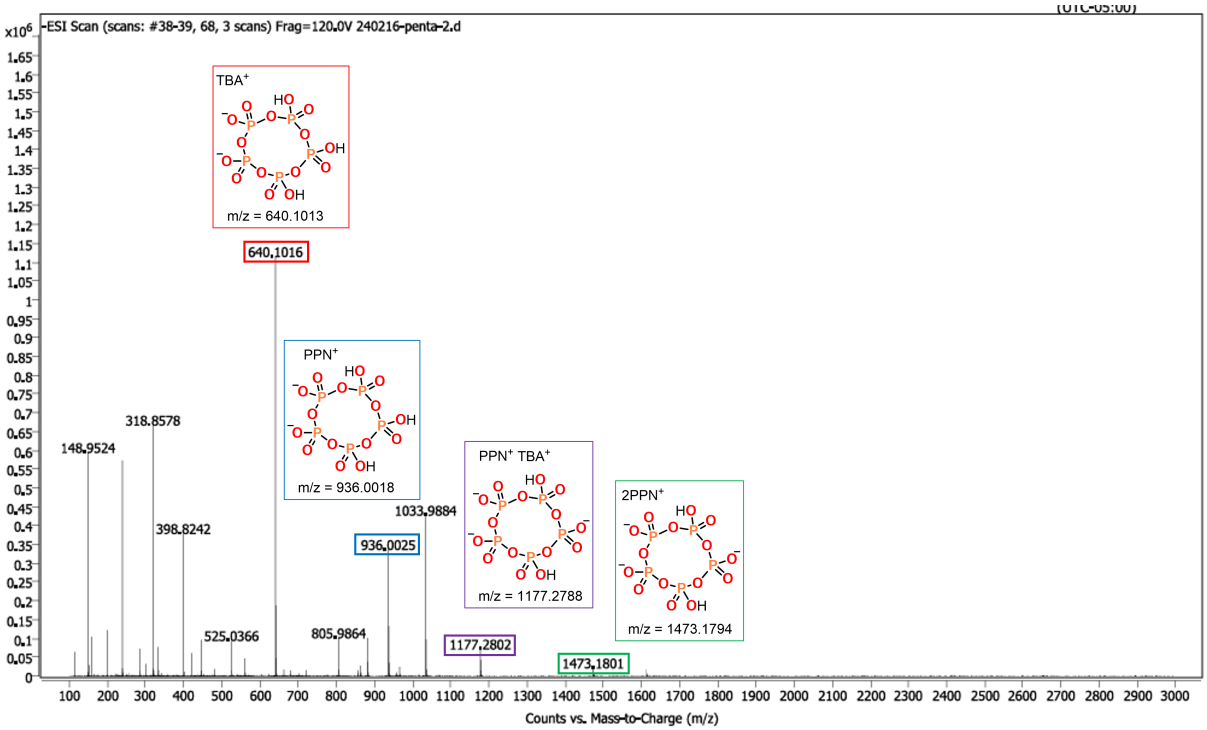

Figure S8: ESI-MS(−) of $[PPN]_2[TBA]\mathbf{2}$ (acetonitrile, 25 μg/mL) with assigned structures showing calculated m/z values.

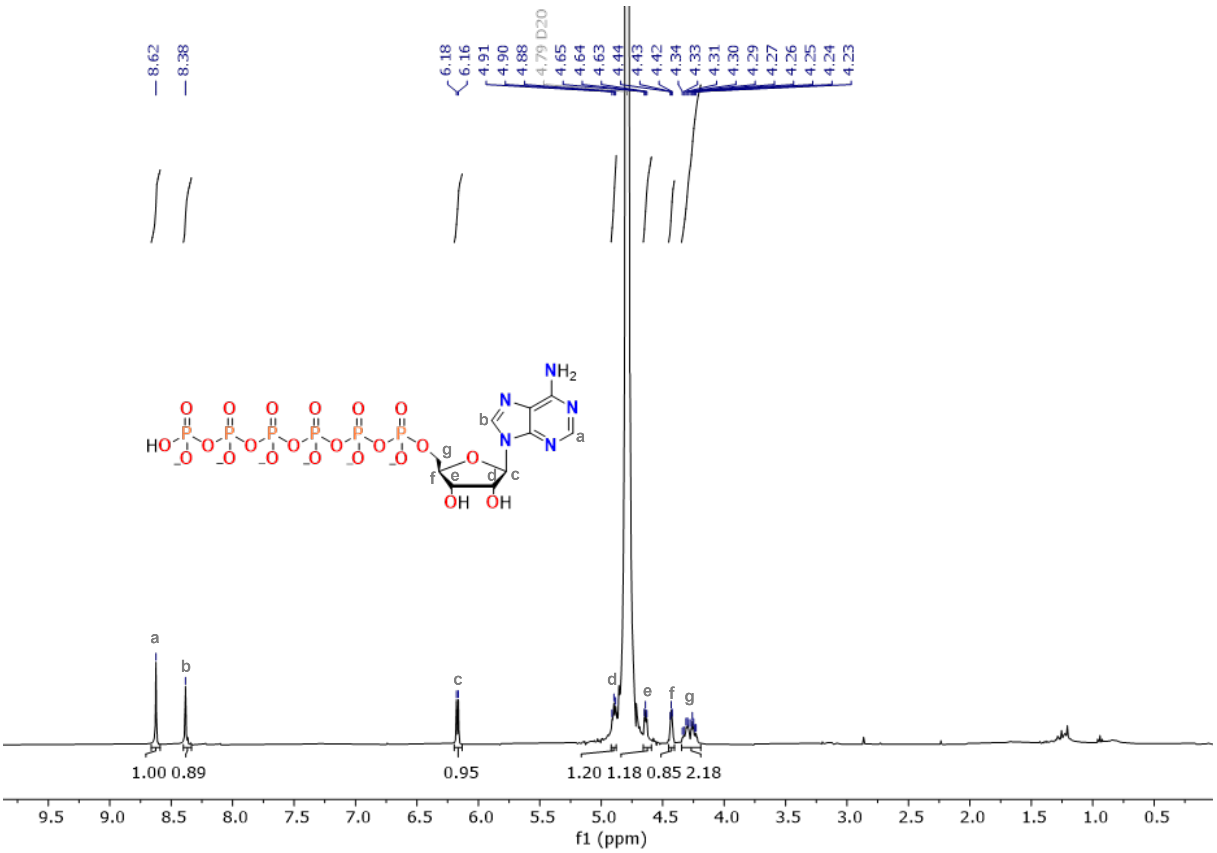

Figure S9: ^{1}H NMR spectrum of $[NH_4]_6[HP_6A]$ in D_2O.

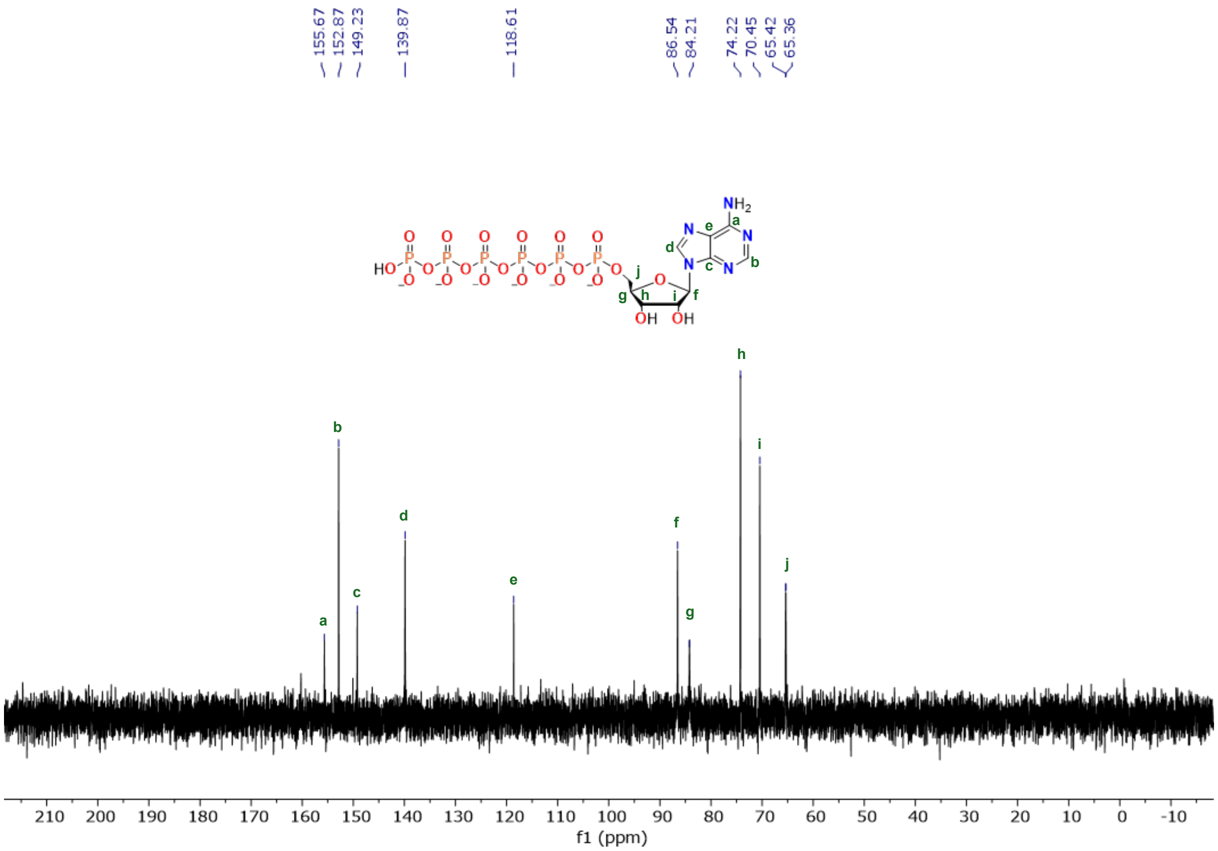

Figure S10: $^{13}C\{^{1}H\}$ NMR spectrum of $[NH_4]_6[HP_6A]$ in D_2O.

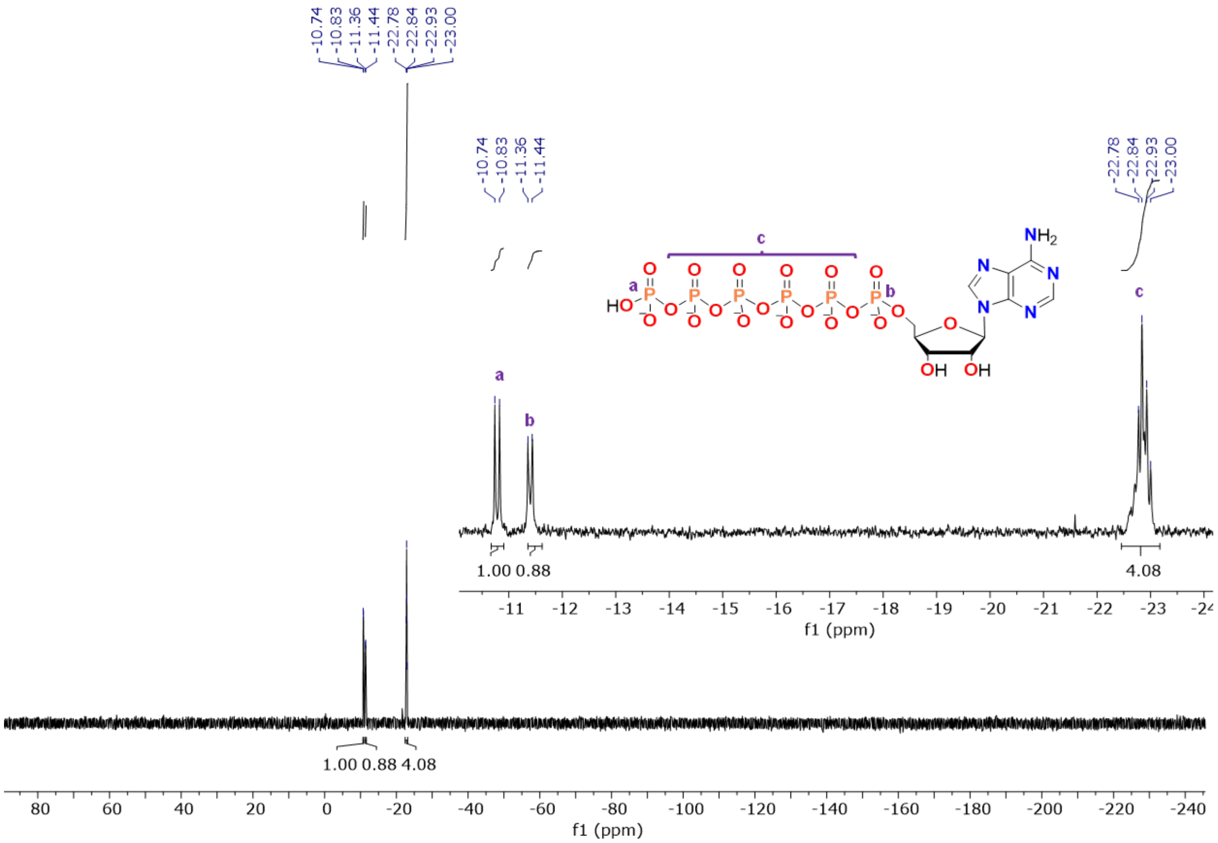

Figure S11: $^{31}P\{^1H\}$ NMR spectrum of $[NH_4]_6[HP_6A]$ in D_2O.

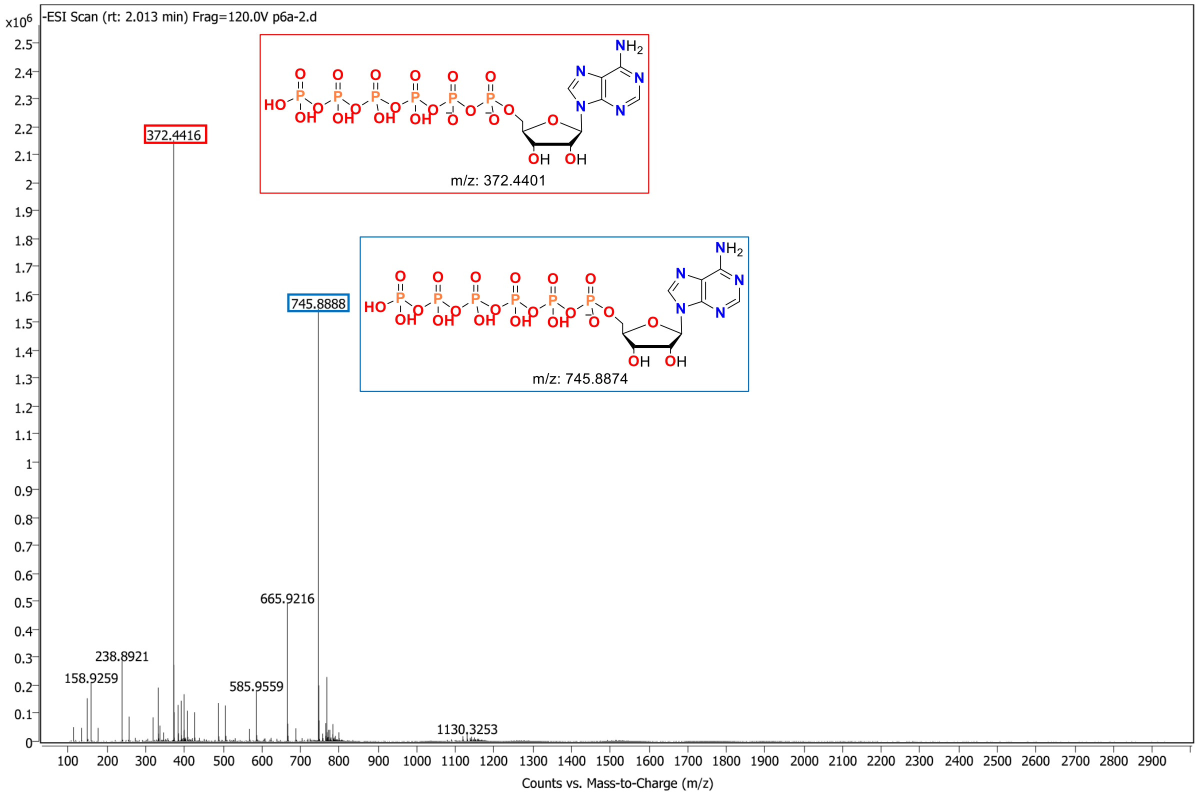

Figure S12: ESI–MS(–) of $[NH_4]_6[HP_6A]$ (water, 25 μg/mL) with assigned structures showing calculated m/z values.

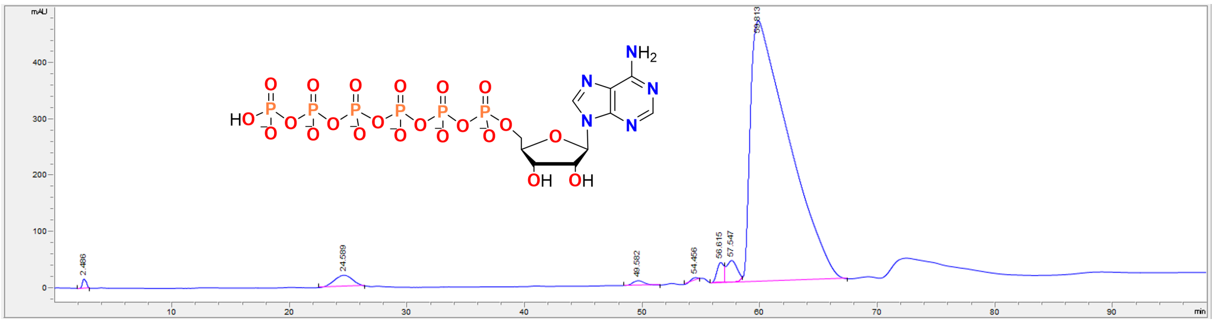

Figure S13: HPLC trace of purified $[NH_4]_6[HP_6A]$. The product peak eluting at 59.813 min integrates to 93.470%. Gradient conditions: 100% A, 0% B (t = 0 to 5 min); 100% A, 0% B to 50% A, 50% B (t = 5 to 65 min); 50% A, 50% B to 0% A, 100% B (t = 65 to 85 min); 0% A, 100% B (t = 85 to 125 min) with a 6 mL/min flow rate and monitored by absorbance at 254 nm. (A = 100% water; B = aqueous 1M ammonium bicarbonate).

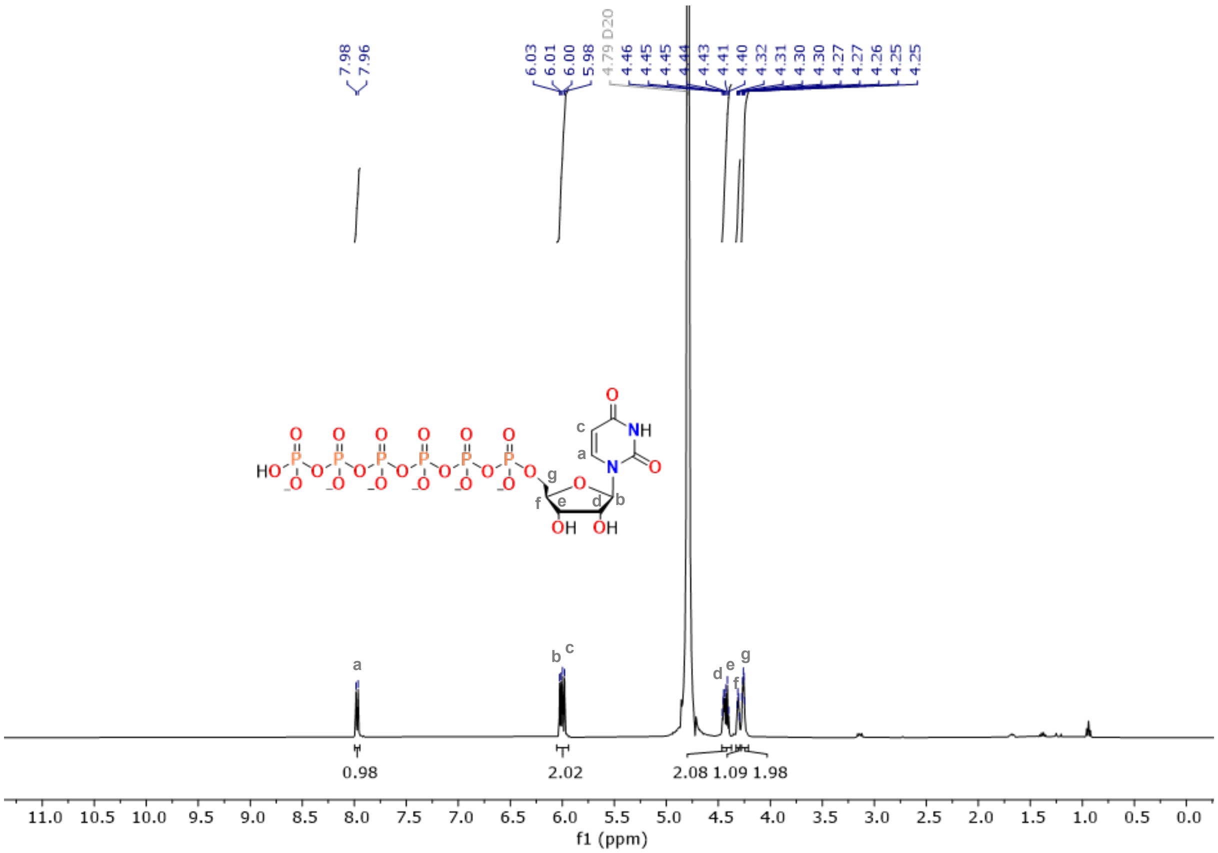

Figure S14: ^{1}H NMR spectrum of $[NH_4]_6[HP_6U]$ in D_2O.

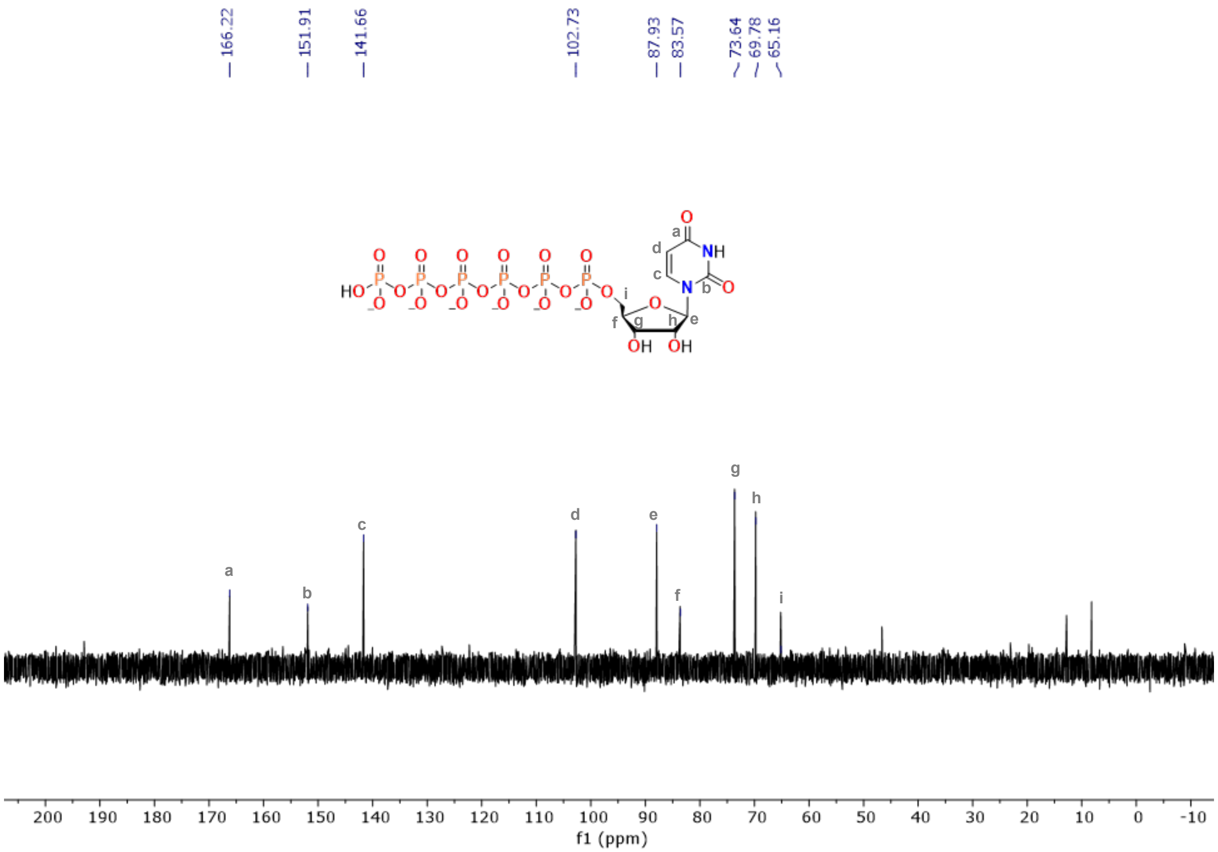

Figure S15: $^{13}C\{^1H\}$ NMR spectrum of $[NH_4]_6[HP_6U]$ in D_2O.

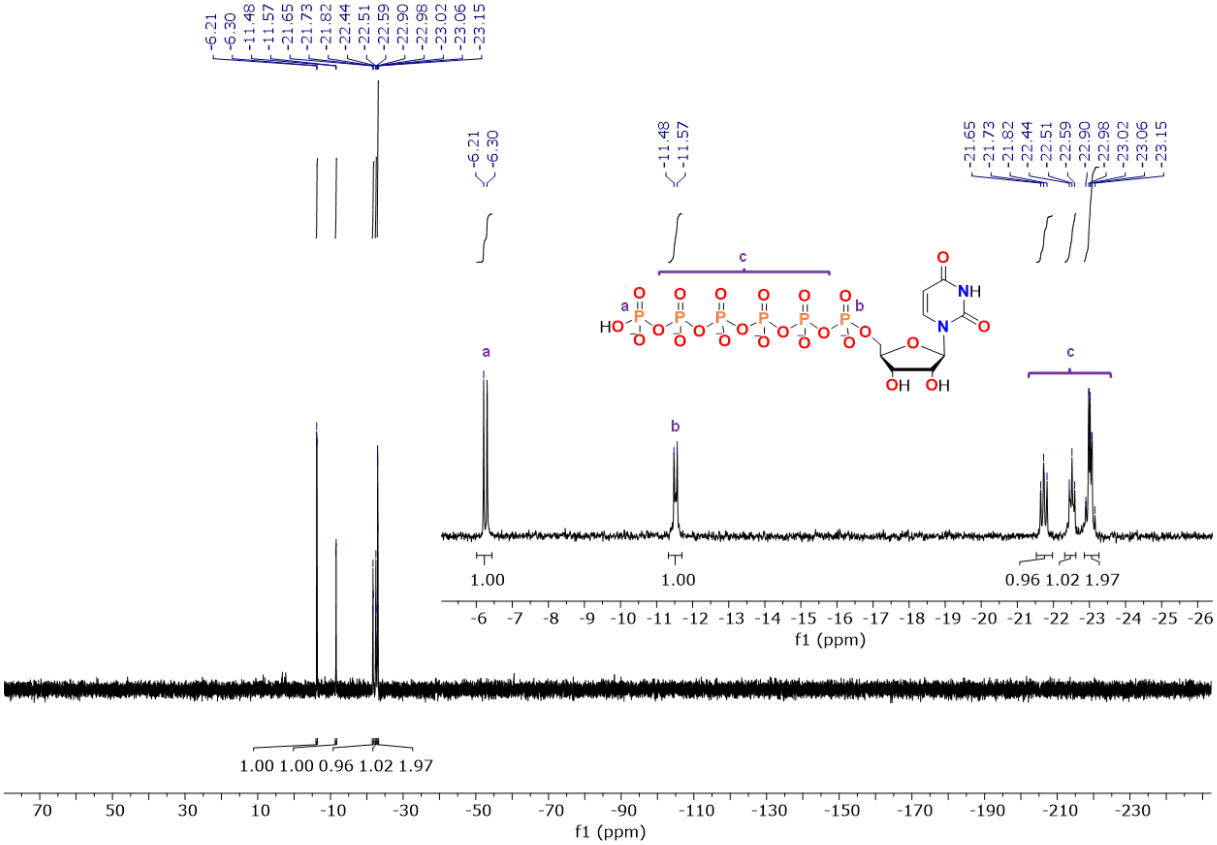

Figure S16: $^{31}P\{^{1}H\}$ NMR spectrum of $[NH_4]_6[HP_6U]$ in D_2O.

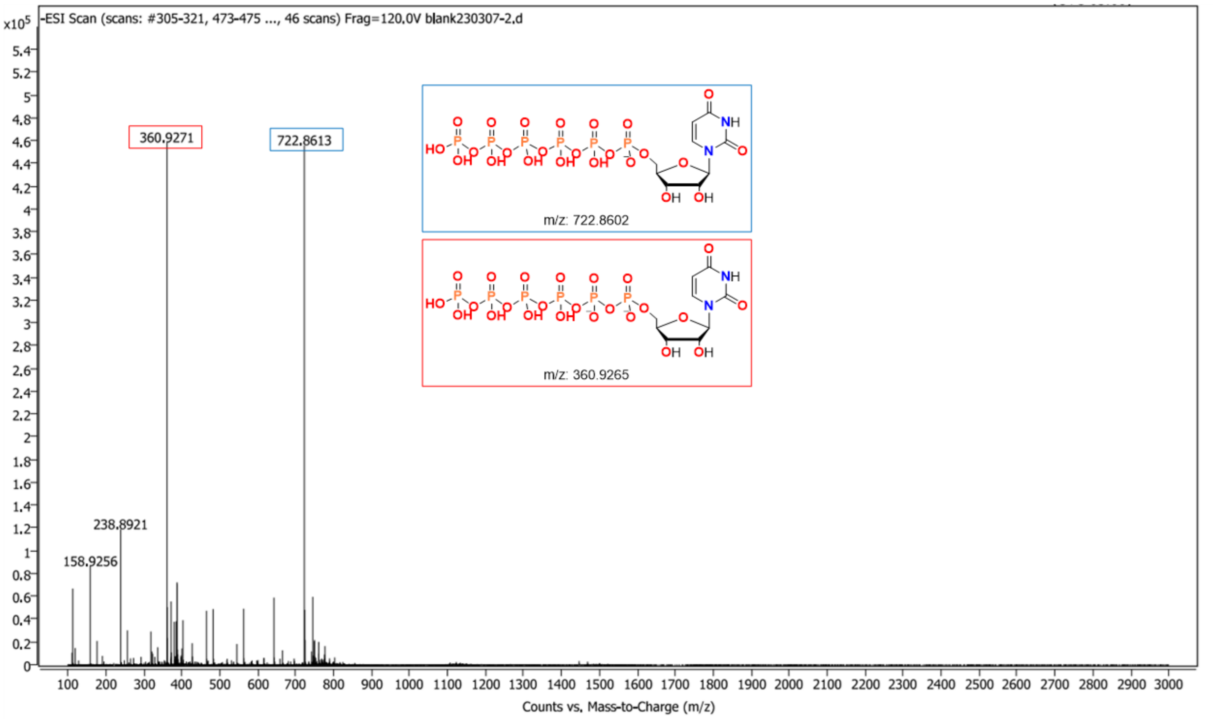

Figure S17: ESI-MS(–) of $[NH_4]_6[HP_6U]$ (water, 25 μg/mL) with assigned structures showing calculated m/z values.

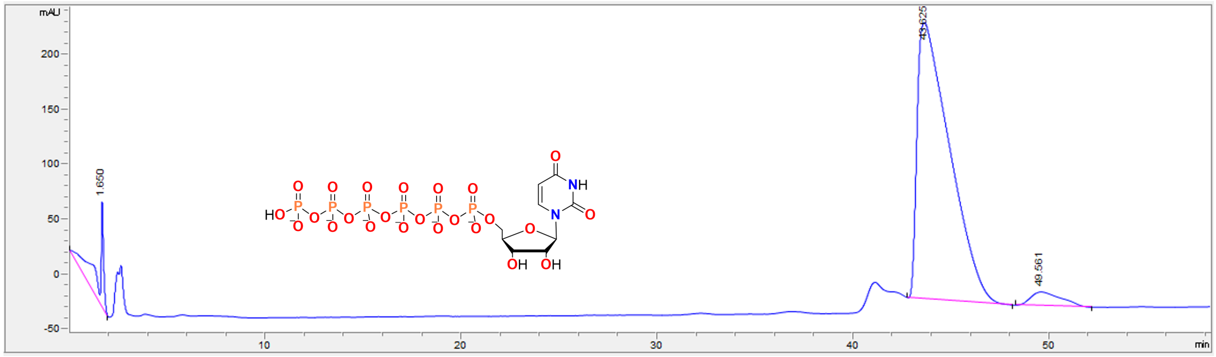

Figure S18: HPLC trace of purified $[NH_4]_6[HP_6U]$. The product peak eluting at 43.625 min integrates to 89.655%. Gradient conditions: 100% A, 0% B (t = 0 to 5 min); 100% A, 0% B to 50% A, 50% B (t = 5 to 65 min); 50% A, 50% B to 0% A, 100% B (t = 65 to 85 min); 0% A, 100% B (t = 85 to 125 min) with a 6 mL/min flow rate and monitored by absorbance at 254 nm. (A = 100% water; B = aqueous 1M ammonium bicarbonate).

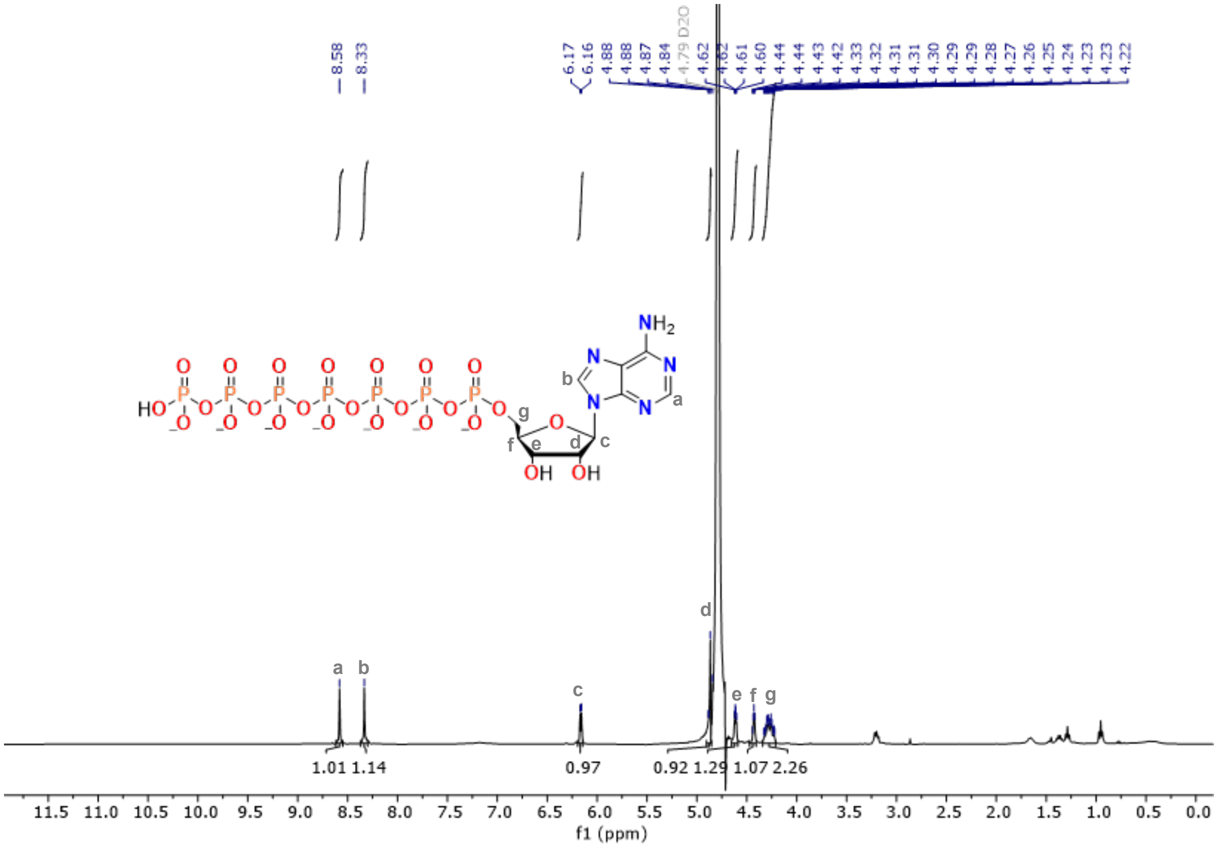

Figure S19: ^{1}H NMR spectrum of $[NH_4]_7[HP_7A]$ in D_2O.

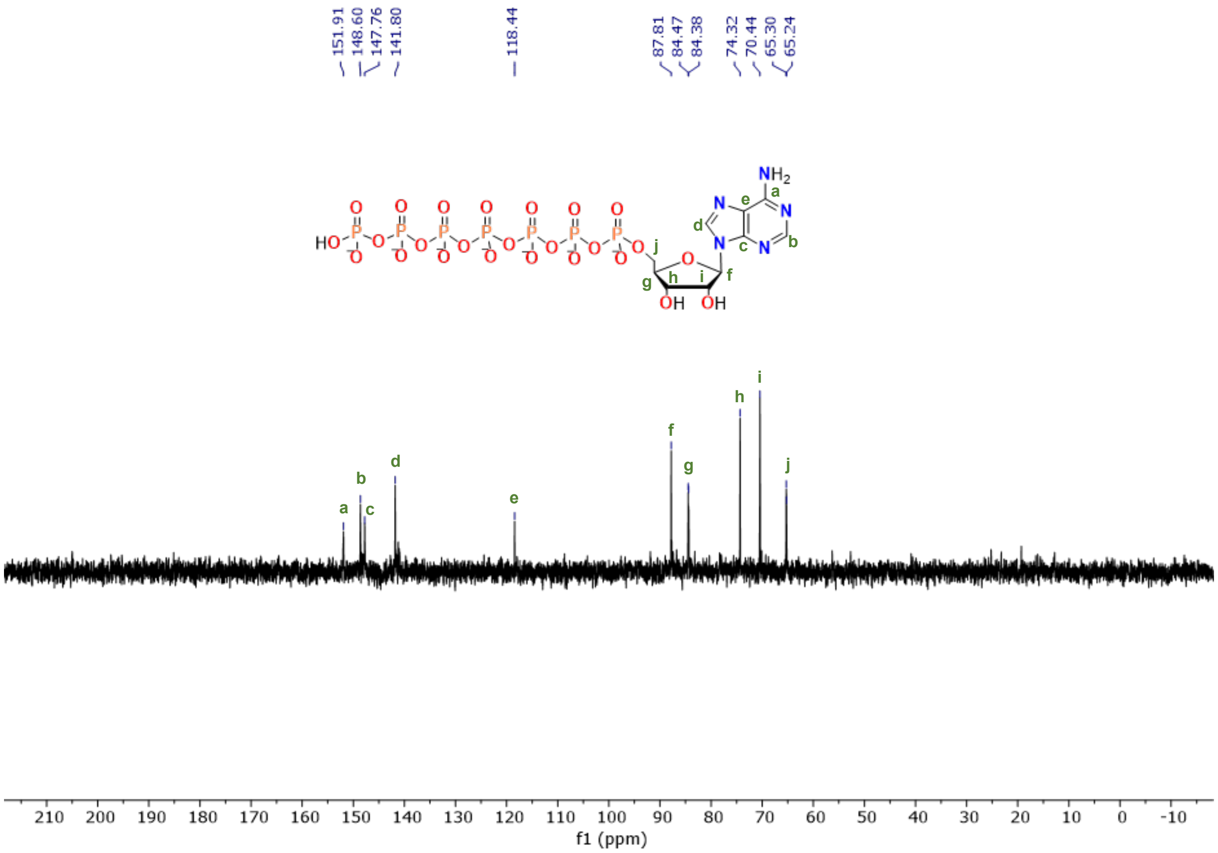

Figure S20: $^{13}C\{^{1}H\}$ NMR spectrum of $[NH_4]_7[HP_7A]$ in D_2O.

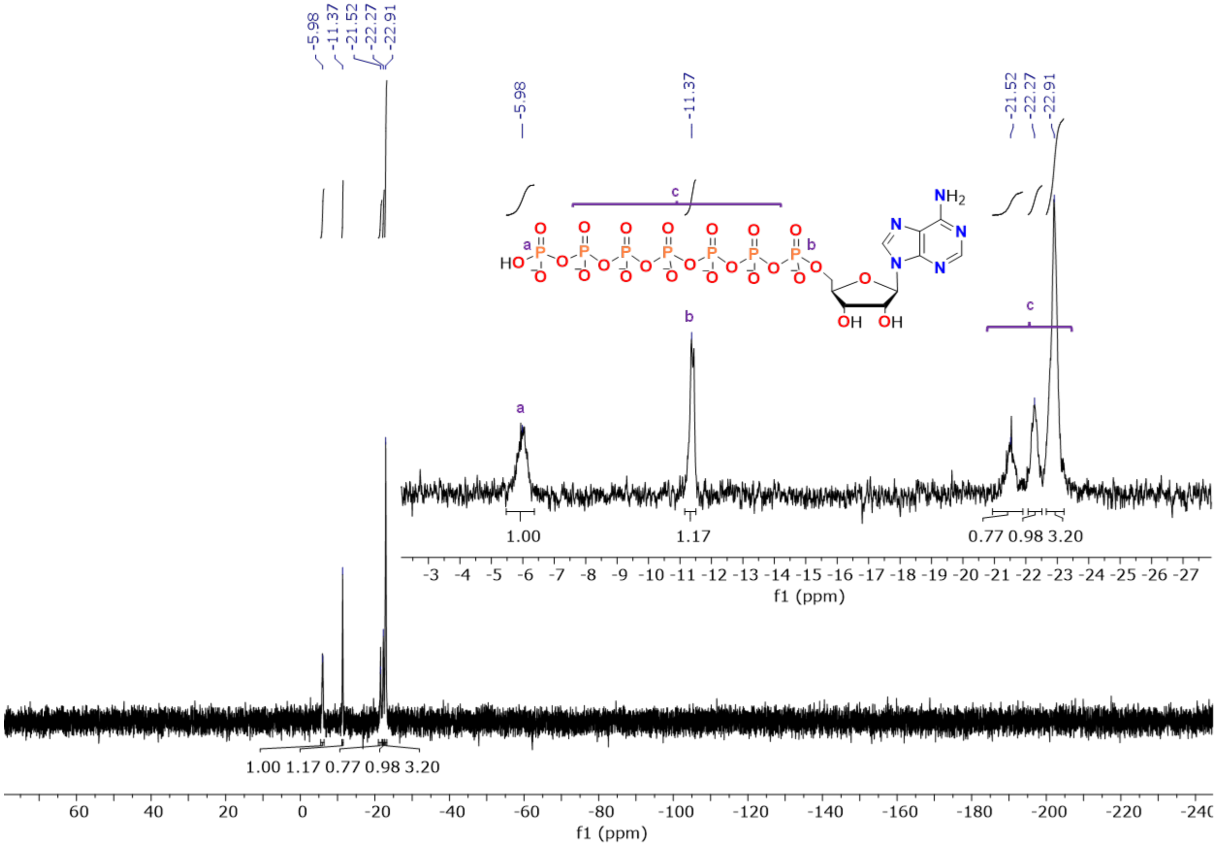

Figure S21: $^{31}P\{^1H\}$ NMR spectrum of $[NH_4]_7[HP_7A]$ in D_2O.

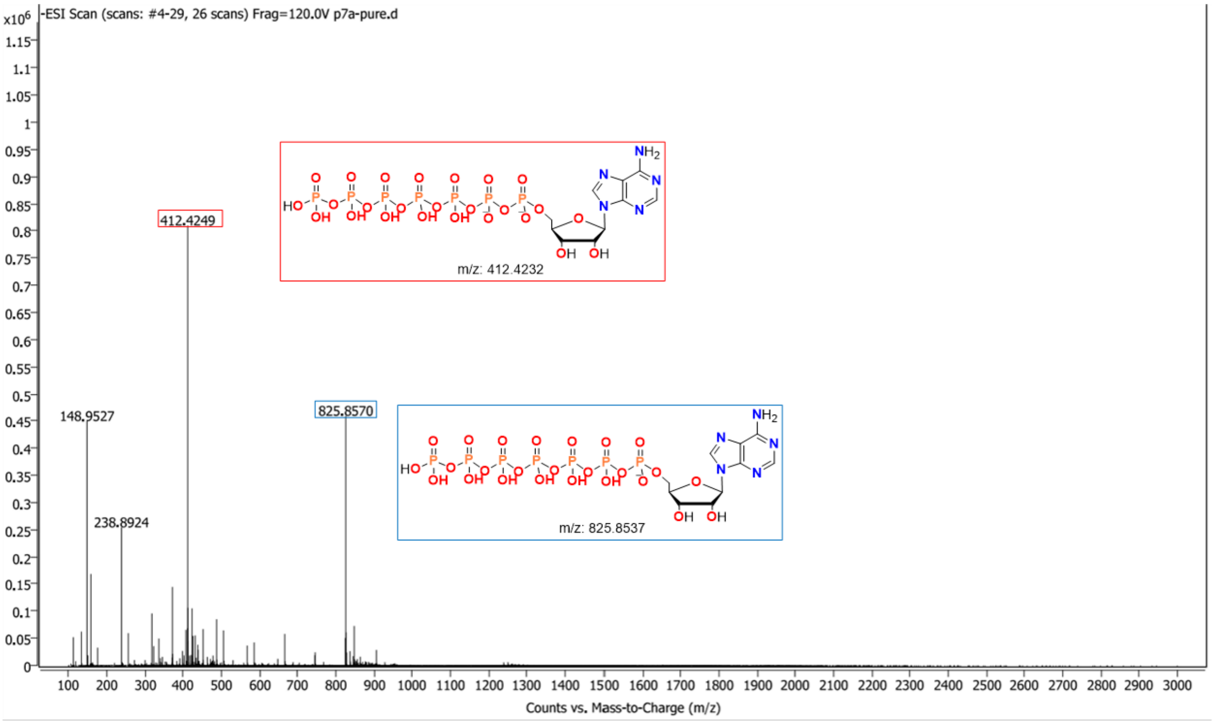

Figure S22: ESI-MS(–) of $[NH_4]_7[HP_7A]$ (water, 25 μg/mL) with assigned structures showing calculated m/z values.

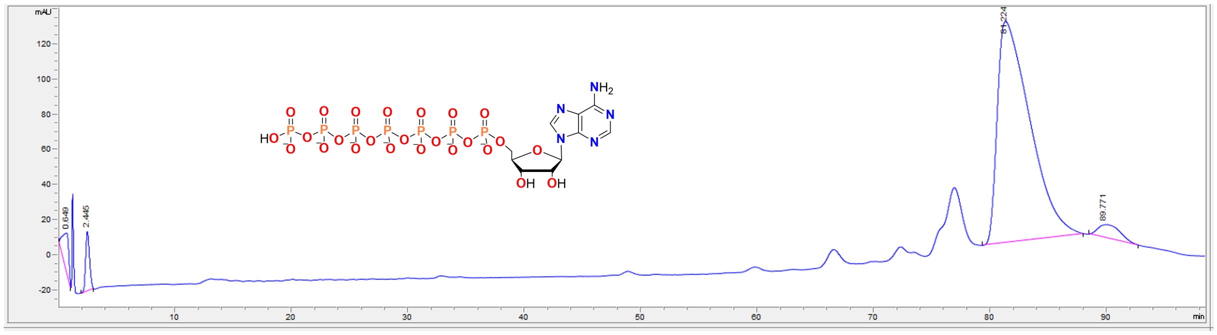

Figure S23: HPLC trace of purified $[NH_4]_7[HP_7A]$. The product peak eluting at 81.224 min integrates to 90.668%. Gradient conditions: 100% A, 0% B (t = 0 to 5 min); 100% A, 0% B to 0% A, 100% B (t = 5 to 170 min) with a 6 mL/min flow rate and monitored by absorbance at 254 nm. (A = 100% water; B = aqueous 1M ammonium bicarbonate).

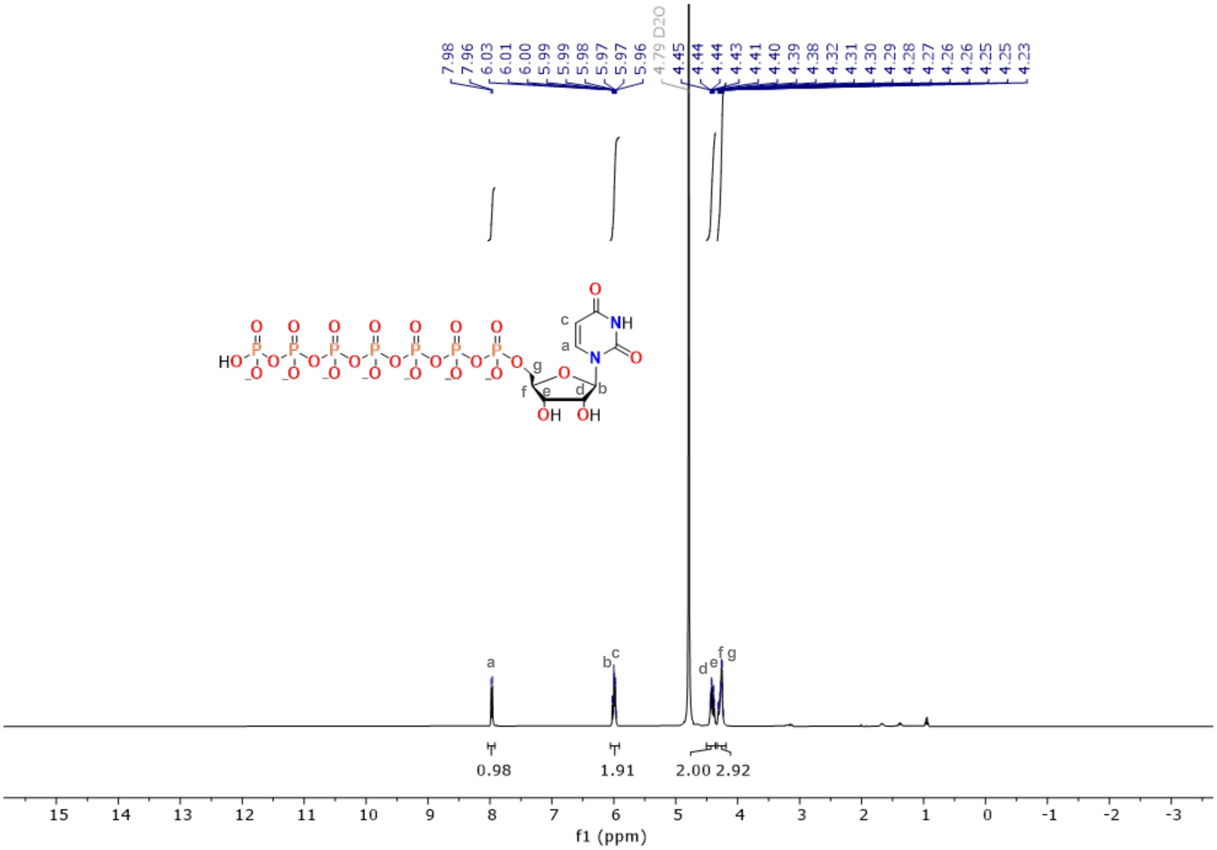

Figure S24: ^{1}H NMR spectrum of $[NH_4]_7[HP_7U]$ in D_2O.

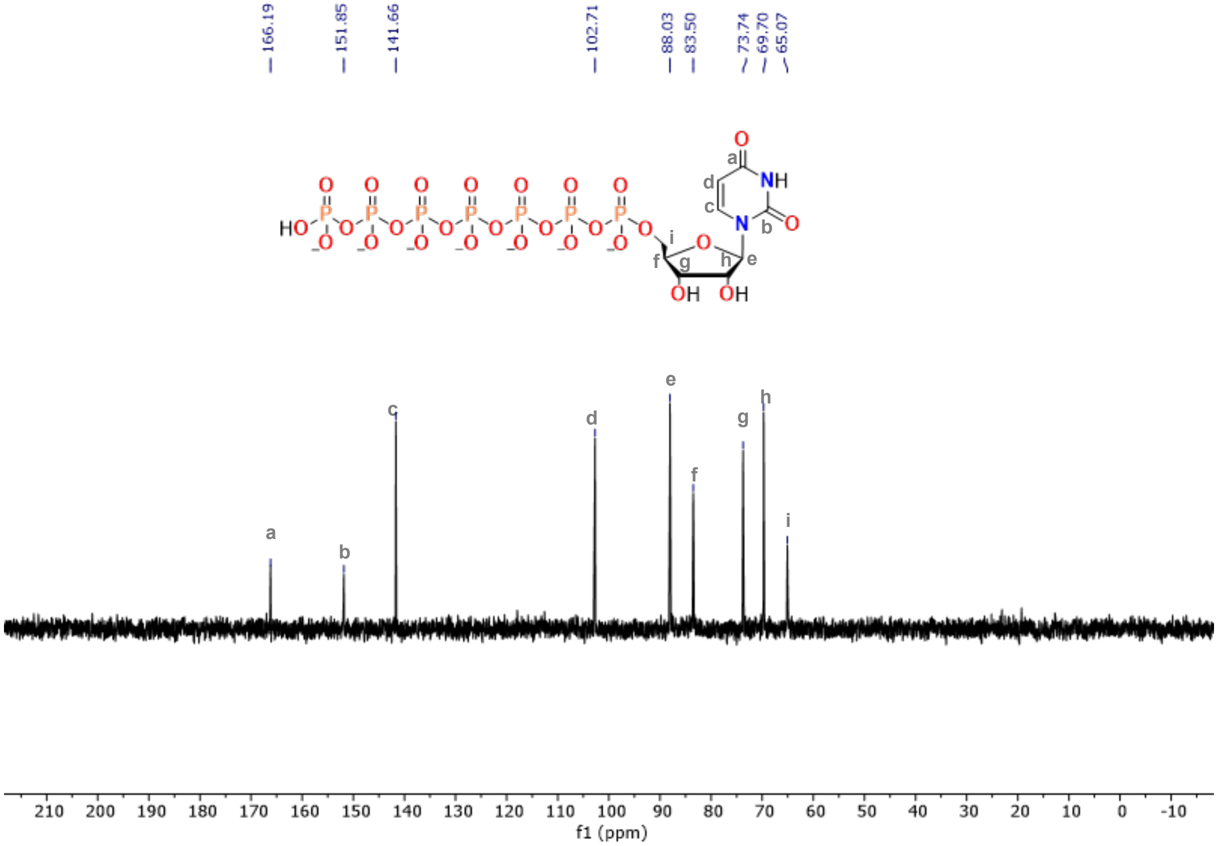

Figure S25: $^{13}C\{^{1}H\}$ NMR spectrum of $[NH_4]_7[HP_7U]$ in D_2O.

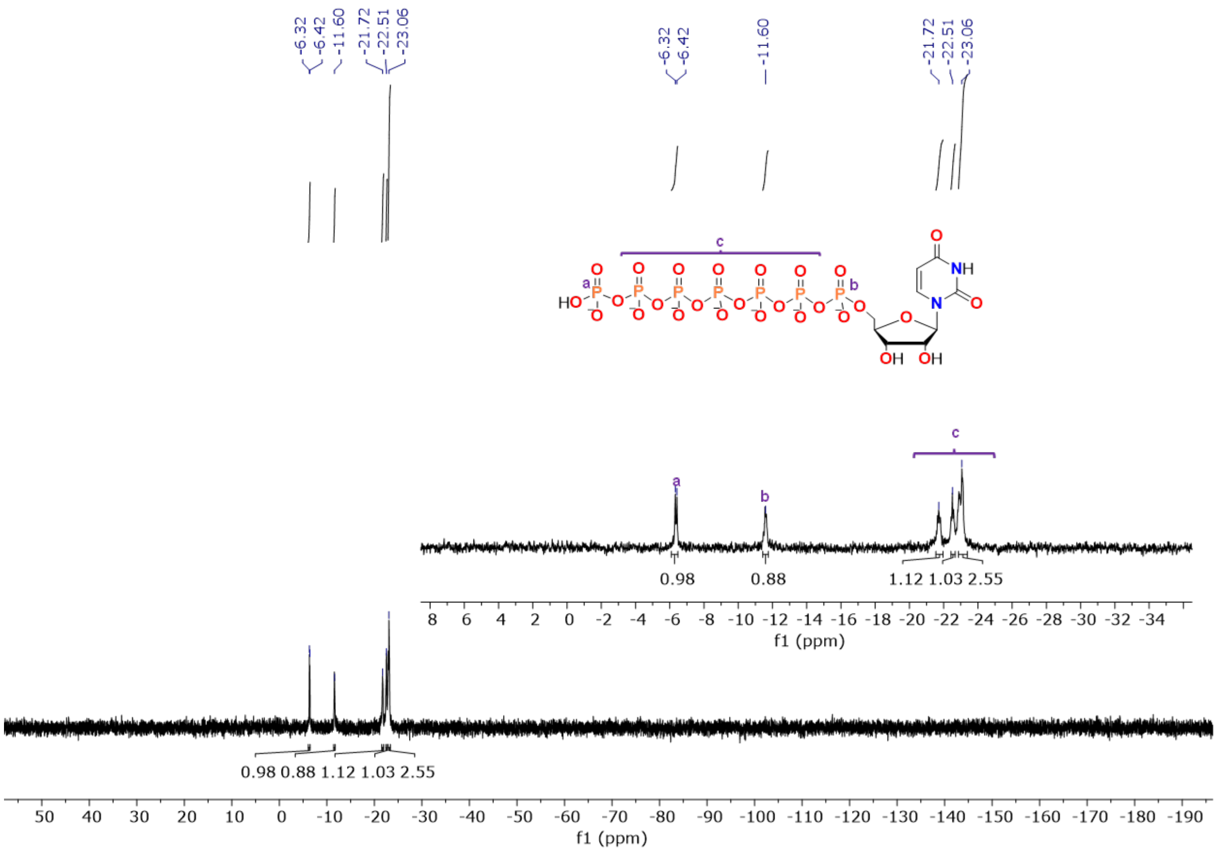

Figure S26: $^{31}P\{^{1}H\}$ NMR spectrum of $[NH_4]_7[HP_7U]$ in D_2O.

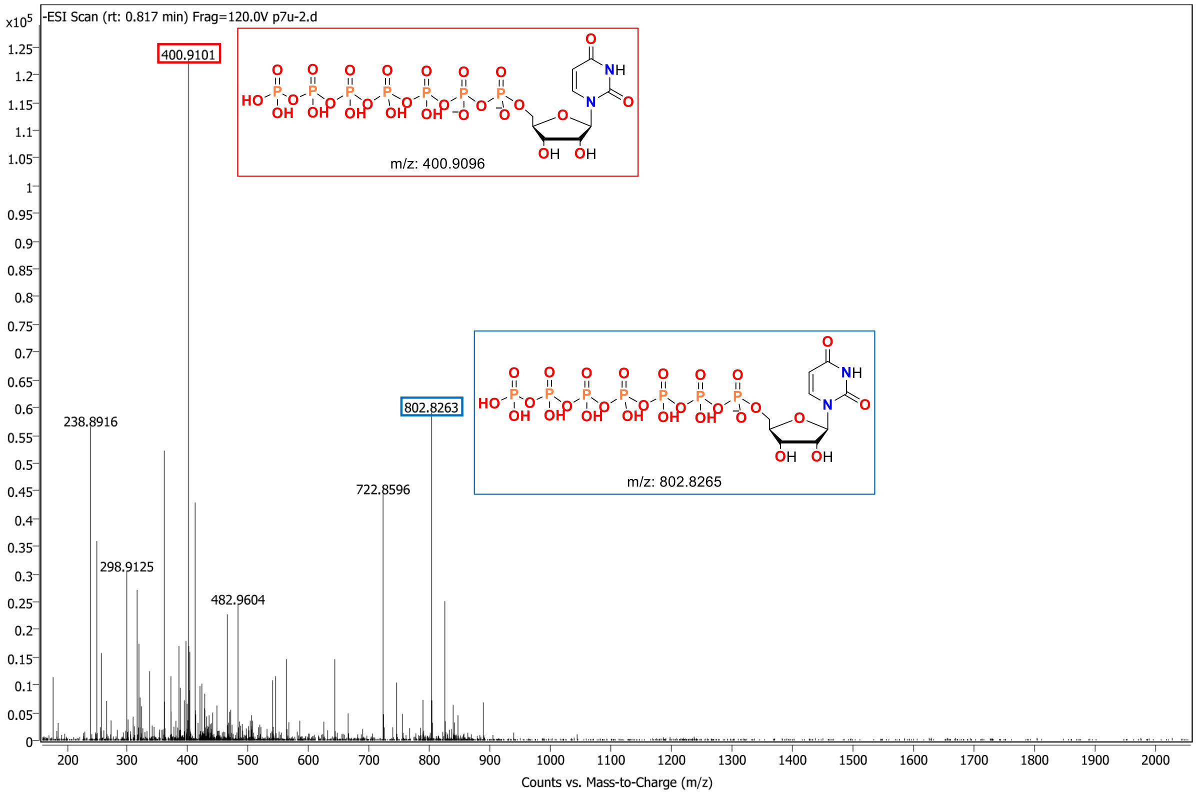

Figure S27: ESI–MS(–) of $[NH_4]_7[HP_7U]$ (water, 25 μg/mL) with assigned structures showing calculated m/z values.

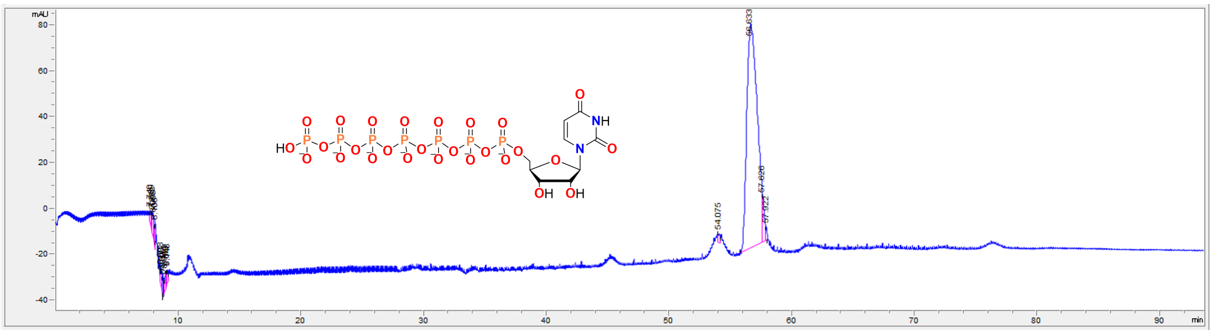

Figure S28: HPLC trace of purified $[NH_4]_7[HP_7U]$. The product peak eluting at 56.633 min integrates to 88.920%. Gradient conditions: 100% A, 0% B (t = 0 to 5 min); 100% A, 0% B to 0% A, 100% B (t = 5 to 170 min) with a 6 mL/min flow rate and monitored by absorbance at 254 nm. (A = 100% water; B = aqueous 1M ammonium bicarbonate).

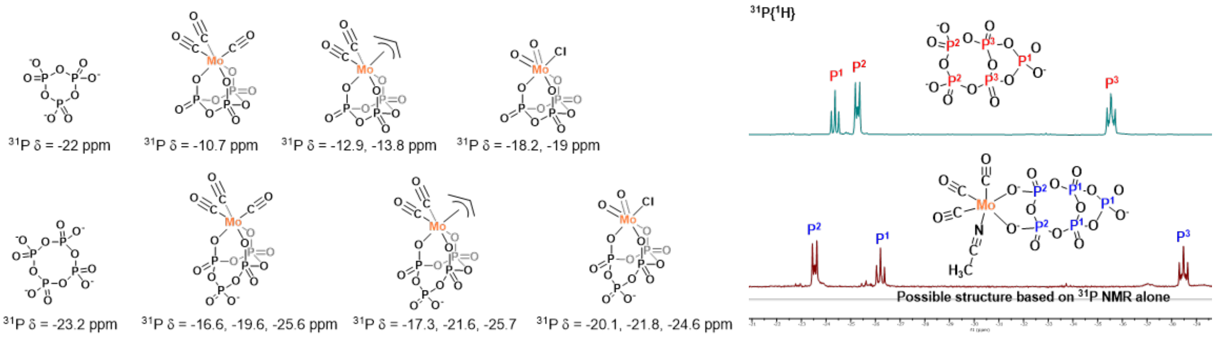

Figure S29: (Left) ^{31}P NMR chemical shifts for tri- and tetrametaphosphates unbound/bound to Mo complexes. (Right) ^{31}P{^{1}H} NMR spectra of **1** and + Mo(NCMe)$_3$(CO)$_3$ and the proposed structure. *Note: Since we don't have the crystal structure of **1** + Mo complex, we cannot determine whether the Mo complex coordinates to the O- of the cyclophosphate substructure of **1** or not. Also, although we cannot rule out the possibility of two ligands coordinated to one Mo, this activation occurred in the presence of more than 1 equiv. of the Mo complex. Therefore, we proposed that the likelihood of two ligands binding to one Mo is less possible.*

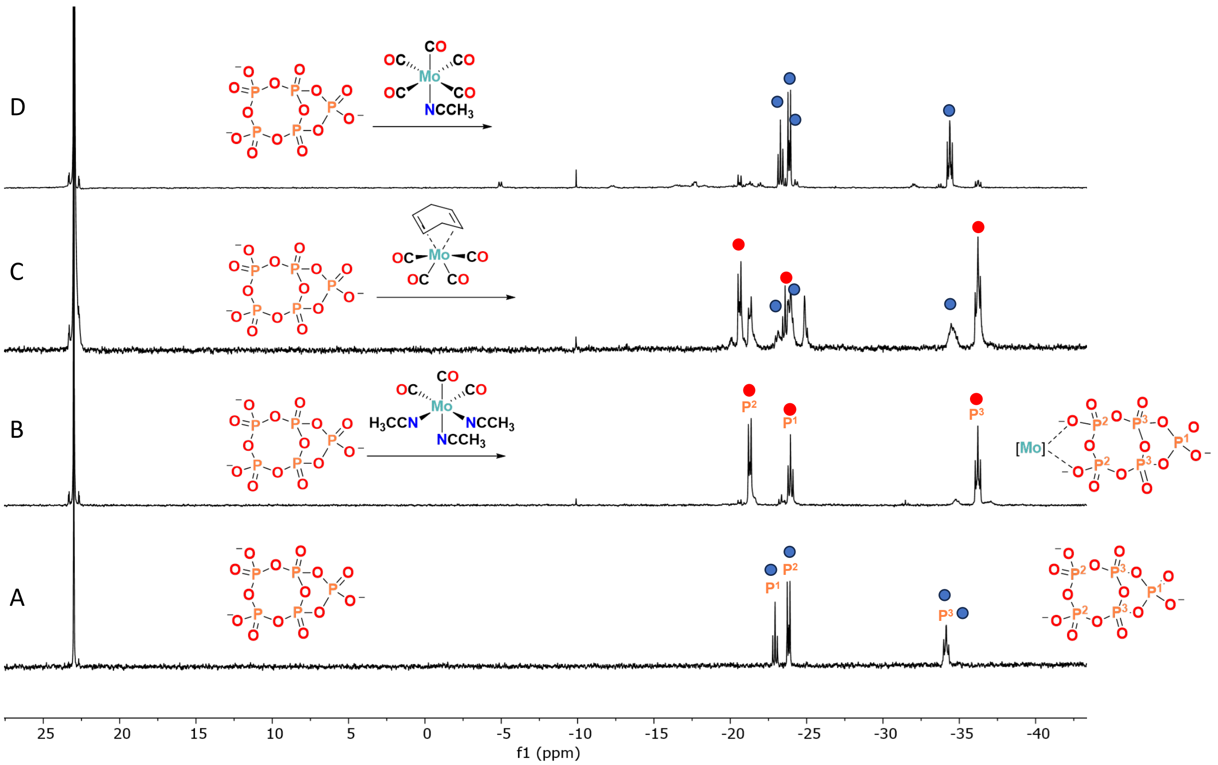

Figure S30: The reaction of **1** with (A) 0 equiv of Mo complex, (B) 1 equiv of $Mo(NCCH_3)_3(CO)_3$, (C) $Mo(NBD)(CO)_4$, and (D) $Mo(NCCH_3)_3(CO)_5$ monitored by $^{31}P\{^1H\}$ NMR spectroscopy. NBD refers to norbornadiene.

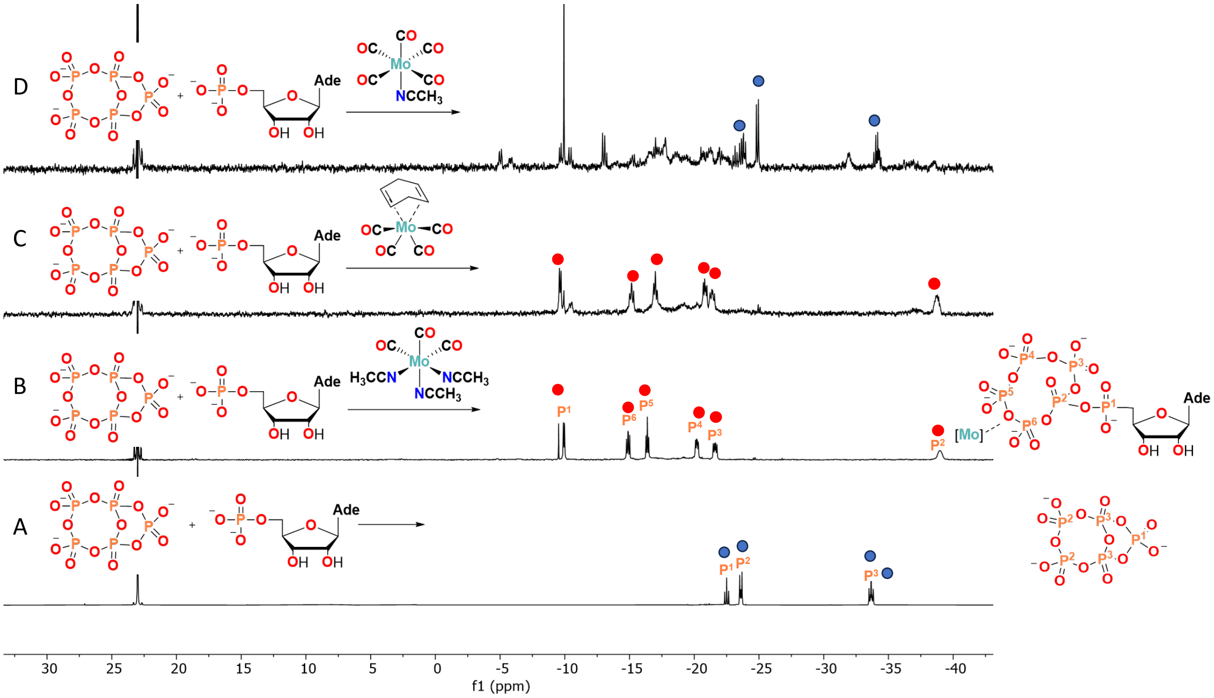

Figure S31: The reaction of **1** with adenosine monophosphate (A) in the absence of Mo complex and in the presence of (B) $Mo(NCCH_3)_3(CO)_3$, (C) $Mo(NBD)(CO)_4$, and (D) $Mo(NCCH_3)_3(CO)_5$ monitored by $^{31}P\{^1H\}$ NMR spectroscopy.

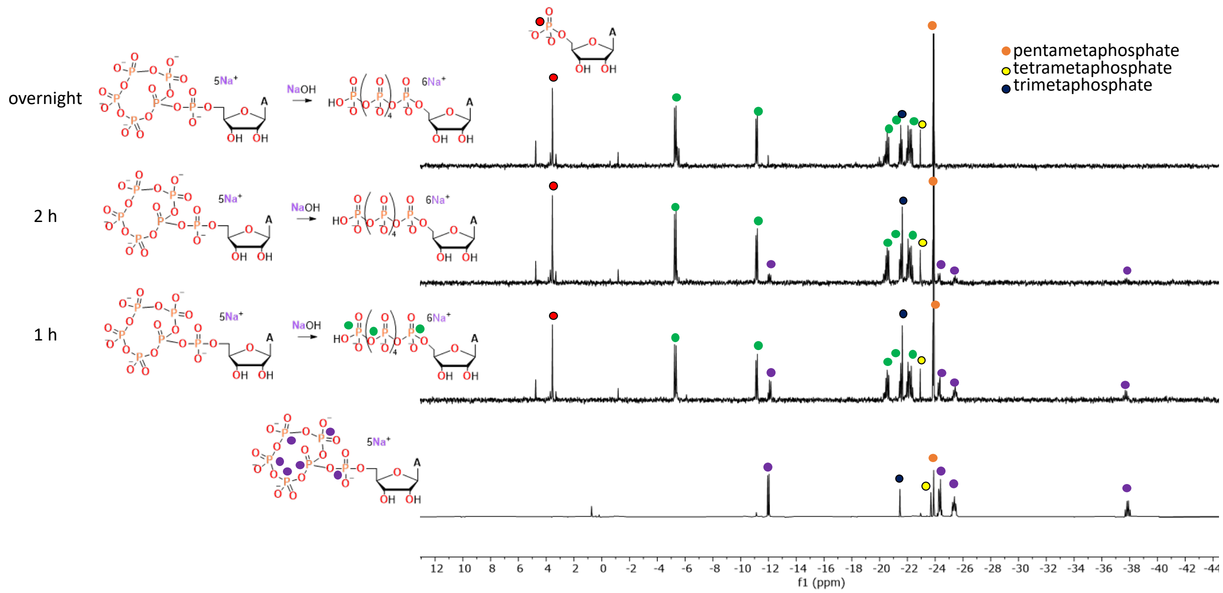

Figure S32: Monitoring of the reaction sequence for the treatment of the phosphatyl-pentametaphosphate adenosine with NaOH by $^{31}P\{^{1}H\}$ NMR spectroscopy with the assignment of the signals.

S3 RNase A Binding Experiments

S3.1 Direct titration method

RNase A binding experiments monitored by $^{31}P\{^{1}H\}$ NMR spectroscopy using $\mathbf{cP}_{4i}$ in 50 mM MES-NaOH buffer.

In an NMR tube, $\mathbf{cP}_{4i}$ (0.68 μmol) was dissolved in 0.6 mL of 50 mM MES-NaOH buffer (free of oligo(vinylsulfonate)), pH 6.0, containing 50 mM NaCl). A stock solution of RNase A (2.2 μmol) in the same buffer solution was added by small increments (0 – 0.5 mL) to the NMR tube. After mixing the solution by shaking it for 30 seconds, the $^{31}P\{^{1}H\}$ NMR spectrum was recorded. The changes in chemical shift $\Delta\delta$ of the phosphorus signal were fitted to a 1:1 binding isotherm to provide the RNase A binding constant using the equation below. The fitting was carried out by hand using Excel.

$$\text{Ligand} + \text{Protein} \xrightarrow{K_{LP}} \text{LP}$$

$$L - x \qquad P - x \qquad x$$

$$K_{LP} = \frac{[LP]}{[L]\cdot[P]} = \frac{x}{(L - x)\cdot(P - x)}$$

$$\Longrightarrow x^2 - x\cdot(L + P + 1/K_{LP}) + L\cdot P$$

$$\Longrightarrow x = \frac{(L + P + 1/K_{LP}) - \sqrt{(L + P + 1/K_{LP})^2 - 4\,L\cdot P}}{2} \qquad \cdots\cdots\cdots (1)$$

$$\delta_{Cal.} = \frac{(L\text{-}x)\delta_L + x\delta_{LP}}{L}$$

$$\delta_{Cal.}\text{-}\delta_L = \frac{x(\delta_{LP}\text{-}\delta_L)}{L}$$

$$\Downarrow$$

$$\Delta\delta = \frac{x(\Delta\delta_{max})}{L} = \Delta\delta_{max}\cdot\frac{(L + P + 1/K_{LP}) - \sqrt{(L + P + 1/K_{LP})^2 - 4\,L\cdot P}}{2L} \qquad \cdots\cdots\cdots (2)$$

With:

L = initial molar concentration of the ligand solution (mol/L)

P = initial molar concentration of the protein (mol/L)

x = molar concetration of the protein-ligand complex solution (mol/L)

K_{LP} = RNase A binding constant of the ligand (M^{-1})

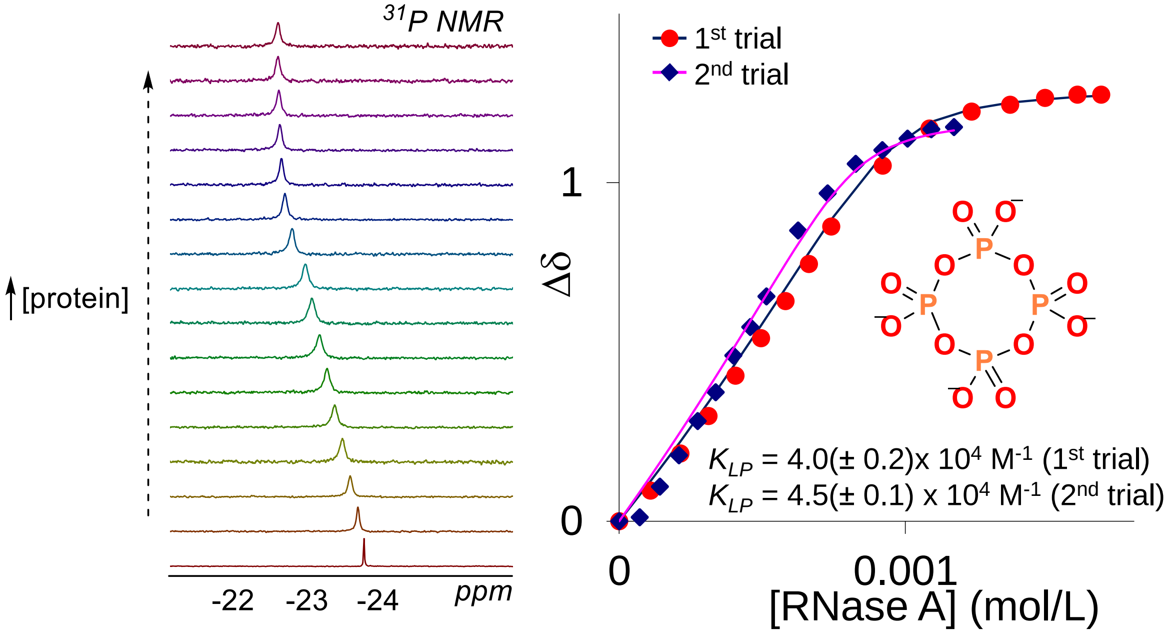

Figure S33: Left: changes observed in $^{31}P\{^1H\}$ NMR spectra upon incremental addition of RNase A (2.2 μmol in 0.5 mL of 50 mM MES-NaOH buffer, bottom to up in left) to a $\mathbf{cP}_{4i}$ (0.68 μmol) solution in 50 mM MES-NaOH buffer. (1st trial data) Right: the fitting of the chemical shift change data according to a 1:1 binding isotherm with K_{LP} = 4.0 x 10^4 M^{-1} (1st trial) and K_{LP} = 4.5 x 10^4 M^{-1} (2nd trial).

S3.2 Indirect titration method

RNase A binding experiments monitored by $^{31}P\{^1H\}$ NMR spectroscopy using $\mathbf{cP_{4i}}$ or $\mathbf{p_3A}$ in 50 mM MES-NaOH buffer.

In an NMR tube, $\mathbf{cP_{4i}}$ or $\mathbf{p_3A}$ (1.5 μmol) was dissolved in 0.6 mL of 50 mM MES-NaOH buffer (free of oligo(vinylsulfonate)), pH 6.0, containing 50 mM NaCl). RNase A (1.5 μmol) in 0.5 mL of the buffer solution was to the NMR tube, affording a 1:1 ligand-protein complex. A stock solution of $\mathbf{p_5U}$, $\mathbf{p_6U}$, and $\mathbf{p_7U}$ (4.5 μmol or 8 μmol or 16 μmol) in 0.1 mL of the buffer solution was added by small increments (0 - 100 μL) to the NMR tube. After mixing the solution by shaking it for 30 seconds, the $^{31}P\{^1H\}$ NMR spectrum was recorded. The changes in chemical shift $\Delta\delta$ of one of the phosphorus signals in the reference compound ($\mathbf{cP_{4i}}$ or $\mathbf{p_3A}$) were fitted to a 1:1 binding isotherm to provide the relative binding constant using the equation below. The fitting was carried out by hand using Excel.

$$\text{Ligand-Protein} + \text{Ligand2} \xrightarrow{K_{rel.}} \text{Ligand2-Protein} + \text{Ligand}$$
$$\quad LP - x \qquad\quad L2 - x \qquad\qquad\qquad x \qquad\qquad x$$

$$K_{rel.} = \frac{[L2P]\cdot[L]}{[LP]\cdot[L2]} = \frac{x^2}{(LP - x)\cdot(L2 - x)}$$

$$\Longrightarrow (1 - 1/K_{rel.})x^2 - (L2 + LP)x + L2\cdot LP = 0$$

$$\Longrightarrow \quad x = \frac{(L2 + LP) + \sqrt{(L2 + LP)^2 - 4(1 - 1/K_{rel.})\cdot L2\cdot LP}}{2\cdot(1 - 1/K_{rel.})} \qquad \cdots\cdots (1)$$

$$\delta_{cal.} = \frac{(LP\text{-}x)\cdot\delta_{LP} + x\cdot\delta_{L2P}}{LP}$$

$$\delta_{cal.} - \delta_{LP} = \frac{x\cdot(\delta_{LP} - \delta_{L2P})}{LP}$$

$$\Downarrow$$

$$\Delta\delta = \frac{x\cdot(\delta_{LP} - \delta_{L2P})}{LP} = \frac{x\cdot\Delta\delta_{max}}{LP}$$

$$= \Delta\delta_{max}\cdot\frac{(L2 + LP) + \sqrt{(L2 + LP)^2 - 4(1 - 1/K_{rel.})\cdot L2\cdot LP}}{2\cdot(1 - 1/K_{rel.})\cdot LP} \qquad \cdots\cdots (2)$$

With:

LP = initial molar concentration of the protein-ligand complex solution (mol/L)

L2 = initial molar concentration of another ligand (ligand2) solution (mol/L)

x = molar concetration of the protein-ligand2 complex solution or ligand solution (mol/L)

$K_{rel.}$ = relative binding constant of the ligands toward RNase A (no unit)

Once we got the $K_{rel.}$, we were able to calculate the binding constant for ligand2 using the equation ($K_{L2} = K_{rel.}$ x K_L) indirectly. With:

$$\text{Ligand-Protein} + \text{Ligand2} \xrightarrow{K_{rel.}} \text{L2-P} + \text{Ligand}$$

$$K_{rel.} = \frac{[\text{L2-P}] \cdot [\text{L}]}{[\text{L-P}] \cdot [\text{L2}]} \quad \cdots\cdots\cdots \ (1)$$

$$\text{Ligand} + \text{Protein} \xrightarrow{K_L} \text{LP}$$

$$K_L = \frac{[\text{LP}]}{[\text{L}] \cdot [\text{P}]} \quad \cdots\cdots\cdots \ (2)$$

$$\text{Ligand2} + \text{Protein} \xrightarrow{K_{L2}} \text{L}_2\text{-P}$$

$$K_{L2} = \frac{[\text{L2-P}]}{[\text{L2}] \cdot [\text{P}]} \quad \cdots\cdots\cdots \ (3)$$

$$\boxed{K_{L2} = K_{rel.} \text{ x } K_L}$$

K_L = RNase A binding relative constant of the ligand (M^{-1})

K_{L2} = RNase A binding relative constant of the ligand2 (M^{-1})

$K_{rel.}$ = relative binding constant of the ligands toward RNase A (no unit)

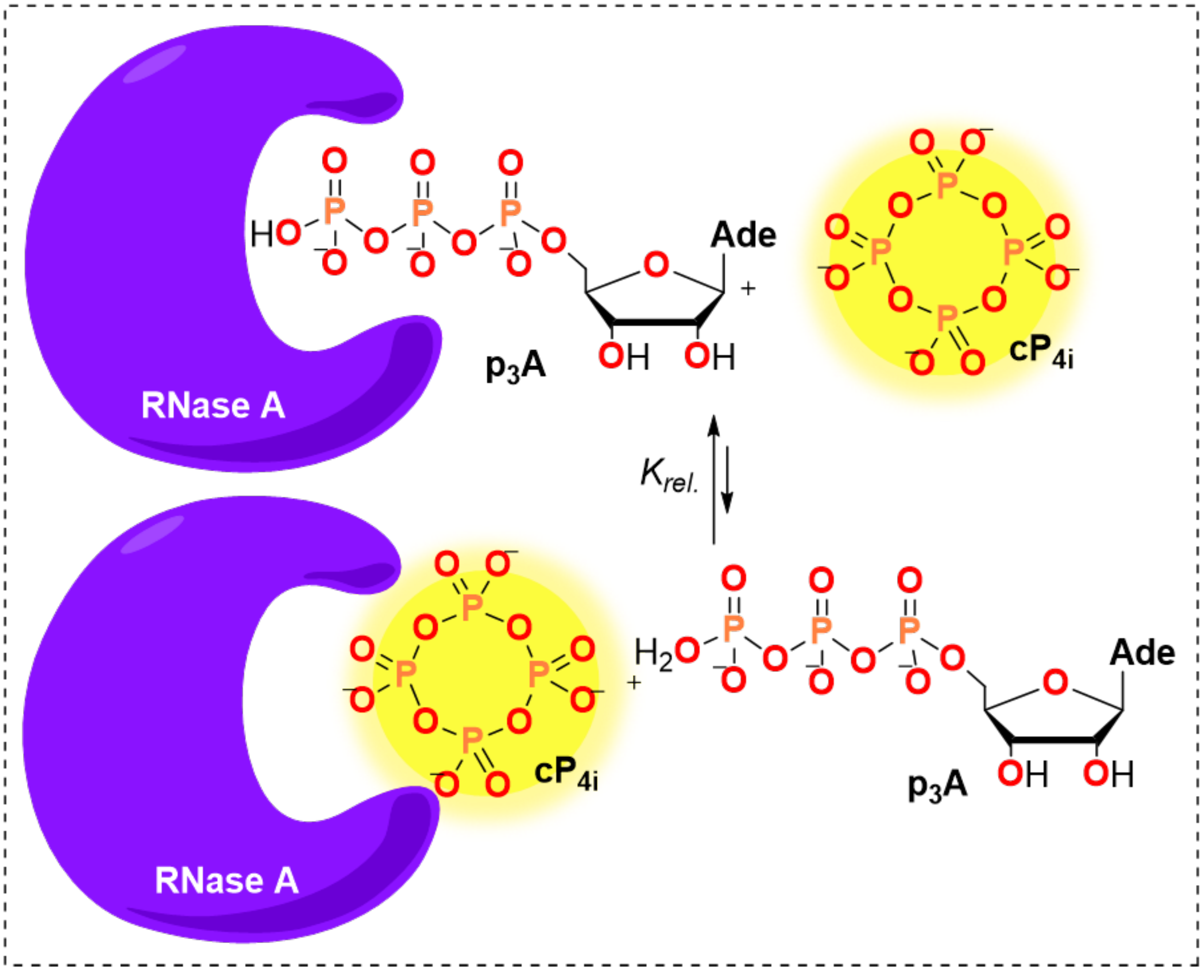

Figure S34: Graphical description of the competition titration of RNase A·$\mathbf{cP}_{4i}$ complex with $\mathbf{p}_3\mathbf{A}$. Ade refers to Adenine.

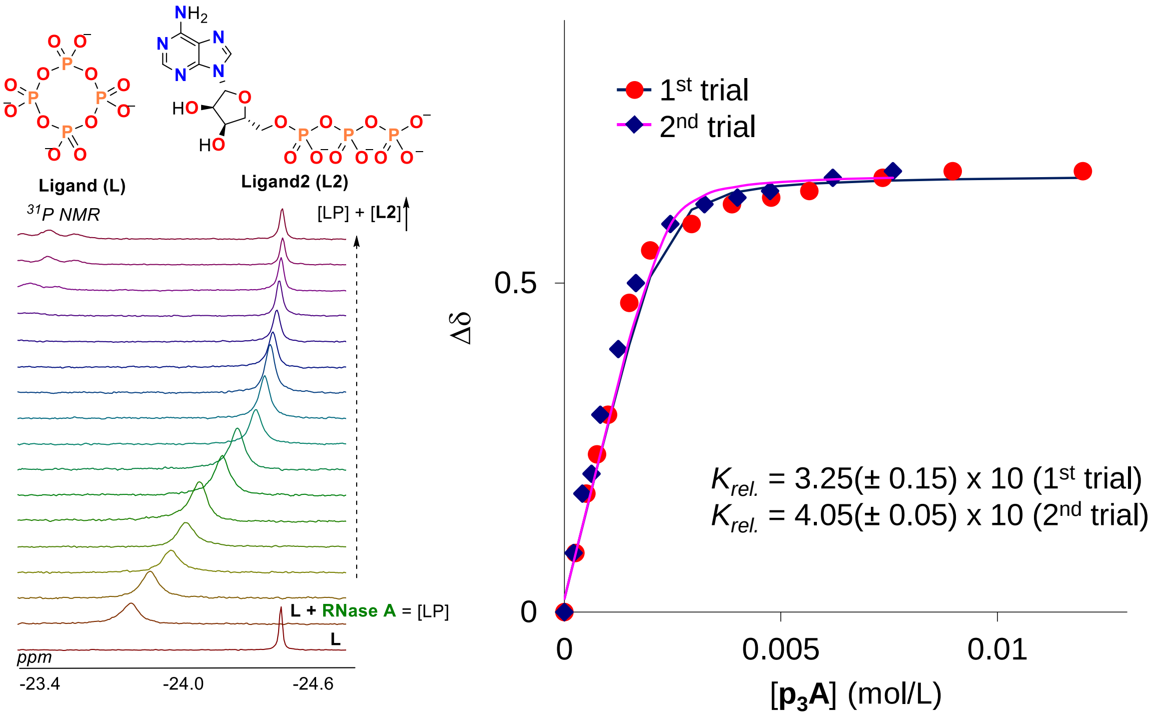

Figure S35: Left: changes observed in ^{31}P{^{1}H} NMR spectra upon incremental addition of $\mathbf{p_3A}$ (16 μmol in 0.1 mL of 50mM MES-NaOH buffer, bottom to up in left) to a RNase A·$\mathbf{cP}_{4i}$ complex solution (1.5 μmol) in 1.1 mL of 50 mM MES-NaOH buffer. (1st trial) Right: the fitting of the chemical shift change data according to a 1:1 binding isotherm with $K_{rel.} = 3.25$ (1st trial) and $K_{rel.} = 4.05$ (2nd trial), affording the association constant ($K_{p3A} = K_{rel.}$ x $K_{cP4i} = 1.55$ x 10^6 M^{-1}).

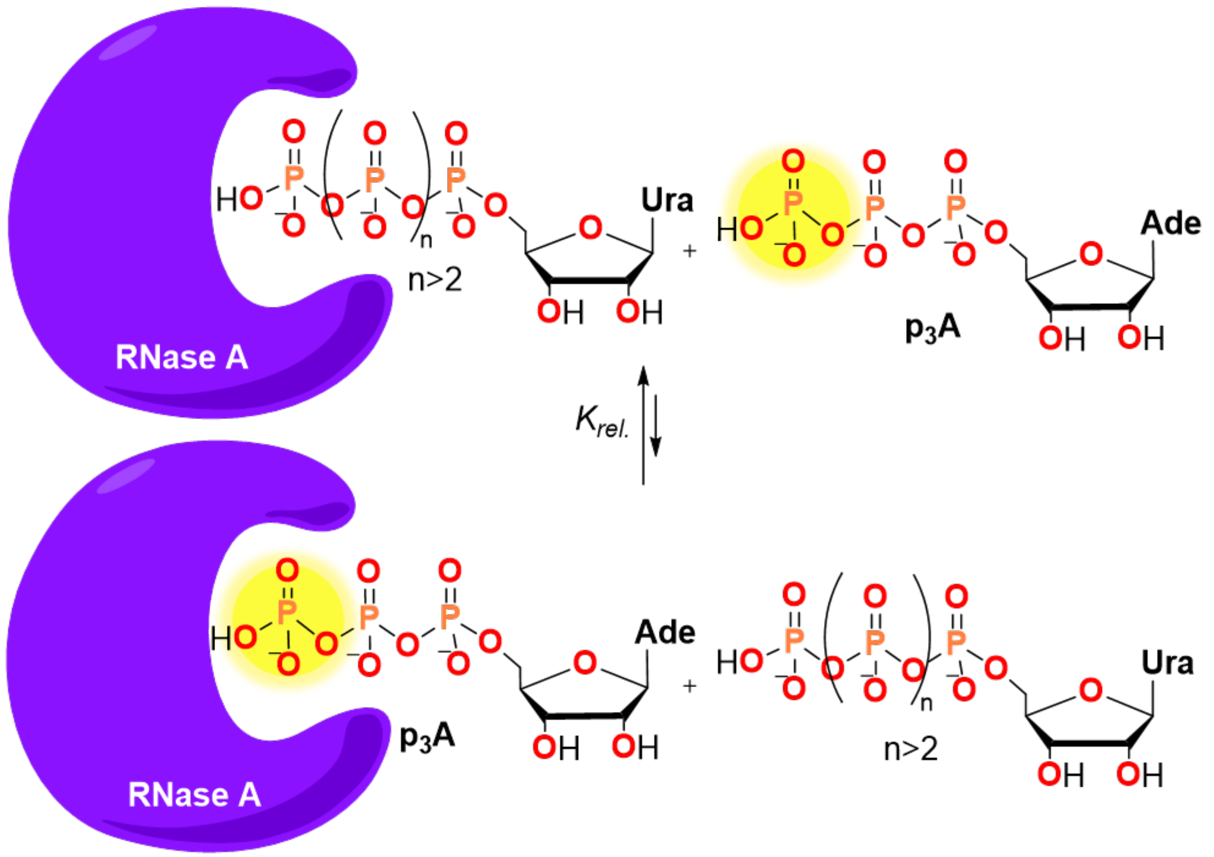

Figure S36: Graphical description of the competition titration of RNase A·$\mathbf{p}_3\mathbf{A}$ complex with $\mathbf{p}_n\mathbf{U}$ (n = 5, 6, or 7). Ura refers to uracil.

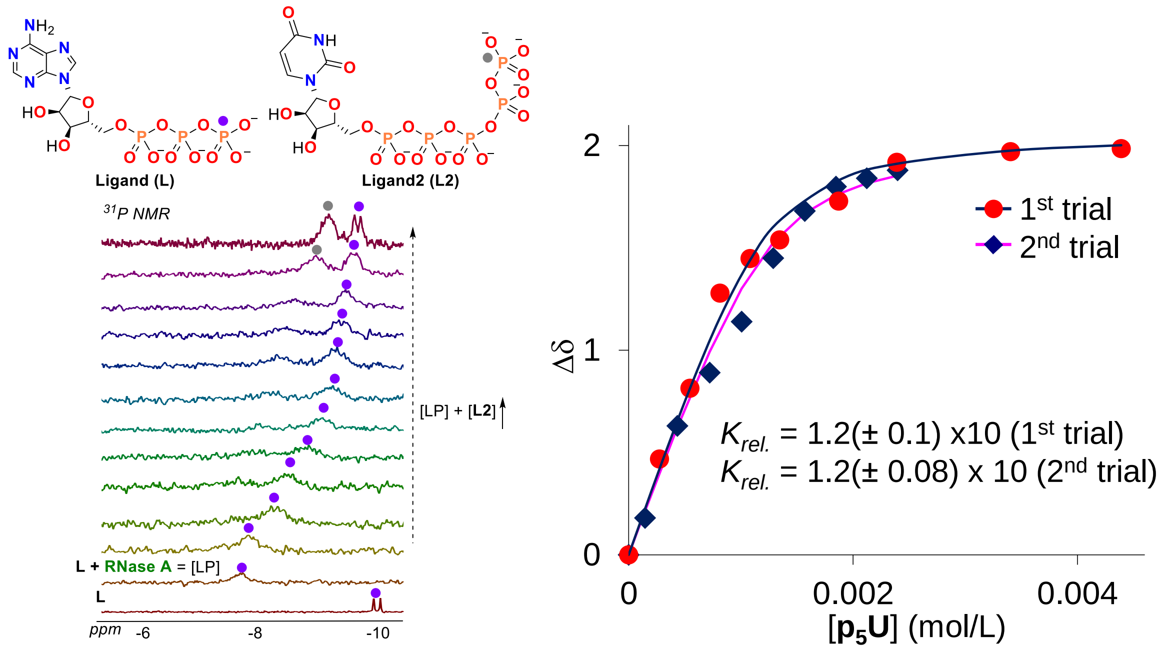

Figure S37: Left: changes observed in ^{31}P{^{1}H} NMR spectra upon incremental addition of $\mathbf{p_5U}$ (8 μmol in 0.1 mL of 50mM MES-NaOH buffer, bottom to up in left) to a RNase A·$\mathbf{p_3A}$ complex solution (1.5 μmol) in 1.1 mL of 50 mM MES-NaOH buffer. (1st trial) Right: the fitting of the chemical shift change data according to a 1:1 binding isotherm with $K_{rel.}$ = 1.2 x 10 (1st trial) and $K_{rel.}$ = 1.2 x 10 (2nd trial), affording the association constant ($K_{p5U} = K_{rel.}$ x K_{p3A} = 1.86 x 10^{7} M^{-1}).

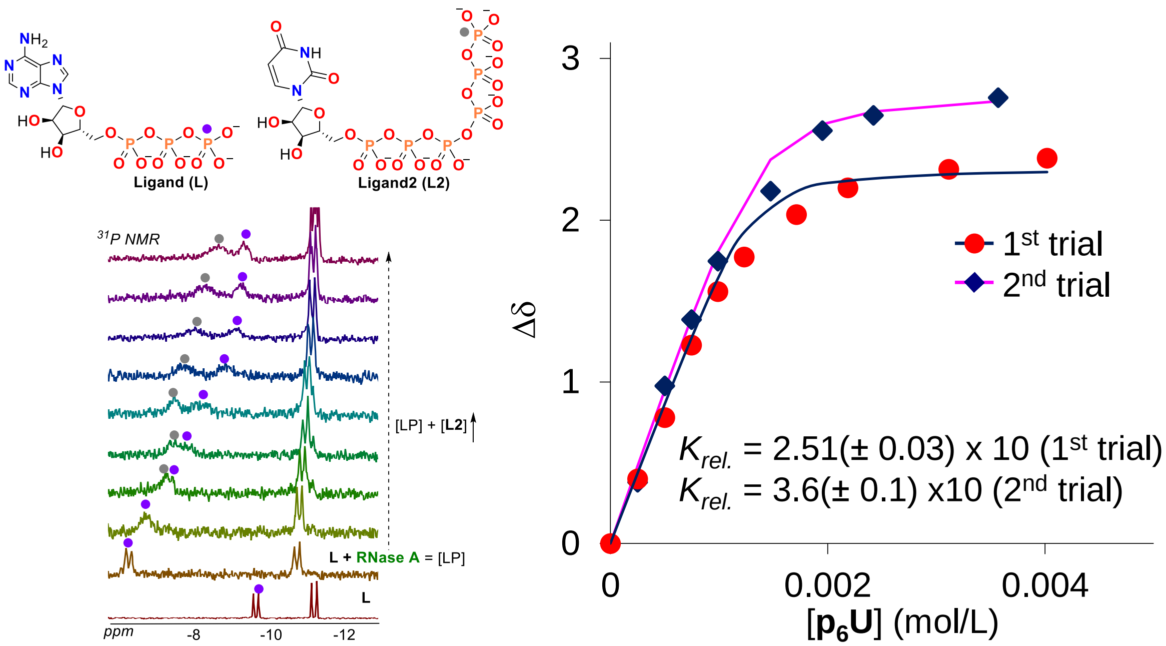

Figure S38: Left: changes observed in $^{31}P\{^1H\}$ NMR spectra upon incremental addition of $\mathbf{p_6U}$ (4.5 μmol in 0.1 mL of 50mM MES-NaOH buffer, bottom to up in left) to a RNase A$\cdot\mathbf{p_3A}$ complex solution (1.5 μmol) in 1.1 mL of 50 mM MES-NaOH buffer. (1st trial) Right: the fitting of the chemical shift change data according to a 1:1 binding isotherm with $K_{rel.} = 2.51$ x 10 (1st trial) and $K_{rel.} = 3.60$ x 10 (2nd trial), affording the association constant ($K_{p6U} = K_{rel.}$ x $K_{p3A} = 4.74$ x 10^7 M^{-1}).

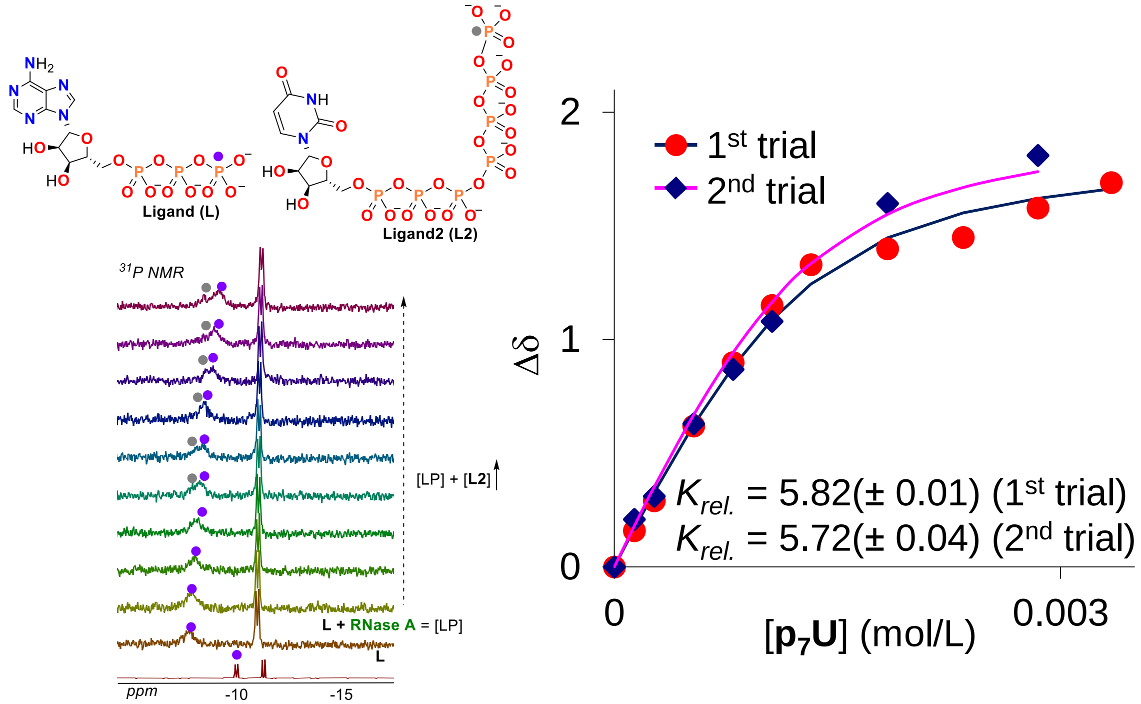

Figure S39: Left: changes observed in ^{31}P{^{1}H} NMR spectra upon incremental addition of $\mathbf{p_7U}$ (4.5 μmol in 0.1 mL of 50mM MES-NaOH buffer, bottom to up in left) to a RNase A·$\mathbf{p_3A}$ complex solution (1.5 μmol) in 1.1 mL of 50 mM MES-NaOH buffer. (1st trial) Right: the fitting of the chemical shift change data according to a 1:1 binding isotherm with $K_{rel.} = 5.82$ (1st trial) and $K_{rel.} = 5.72$ (2nd trial), affording the association constant ($K_{p7U} = K_{rel.}$ x $K_{p3A} = 8.94$ x 10^6 M^{-1}).

S4 RNase A Inhibition Kinetics

RNase A inhibition assays to determine K_i.

Wherever possible, certified RNase-free labware was used to prevent assay contamination with environmental RNases. Likewise, all solutions were previously DEPC-treated to inactivate contaminating RNases in solution. Experimental working areas were routinely wiped down with an RNase-inactivating spray.

Bovine ribonuclease A (Sigma, catalog no. R6513) was dissolved in RNase A activity assay buffer (50 mM MES-NaOH (free of oligo(vinylsulfonate)), 100 mM NaCl, pH 6.0, DEPC-treated before use) to afford an approximately 200 µM enzyme stock;[S5] the enzyme stock concentration was precisely quantified by UV absorption ($\varepsilon_{280\text{ nm}}$ = 9,440 M^{-1} s^{-1}, calculated using Prot pi), and single-use aliquots of the enzyme stock were flash-frozen in liquid nitrogen and stored at -70 °C. Immediately before assaying one inhibitor compound, a single aliquot of enzyme stock was quickly thawed and diluted to afford a 4× enzyme stock at 160 pM.

Each inhibitor compound was weighed and dissolved in RNase A activity assay buffer to yield approximately 4 mM compound. The precise concentration of each compound stock was quantified by UV absorption ($\varepsilon_{260\text{ nm}}$ = 9,660 M^{-1} s^{-1} for uridine phosphates, $\varepsilon_{260\text{ nm}}$ = 15,020 M^{-1} s^{-1} for adenosine phosphates).[S6] A 4× dilution series of each compound was prepared, ranging from 400 µM to 400 pM. Immediately after preparing each compound dilution series, equal volumes of 4× enzyme stock and 4× compound stock were mixed (to yield 2× = 80 pM enzyme stock and 2× compound dilutions, ranging from 200 µM to 200 pM) and allowed to incubate for 15 min at room temperature without agitation.

The FRET-tagged, chimeric tetranucleotide substrate 6-FAM–dA$\underline{\text{r}}$UdAdA–6-TAMRA was used as a substrate for the RNase A activity assay (see Figure S40). This oligonu-

cleotide (supplied by Microsynth) is routinely prepared as a 40 µM stock solution in DEPC-treated ultrapure water, and is kept at -20 °C with a seven-month solution shelf-life as suggested by Microsynth. Just before assay of RNase A activity, the oligonucleotide was thawed, diluted to 160 nM with RNase A activity assay buffer, and plated in a black, flat/opaque bottom, 96-well microplate (quadruplicate wells per experimental condition). To verify that the substrate was free of RNase contamination, the fluorescence of all wells was monitored over a 2 minute period at $\lambda_{\text{ex}} = 493$ nm (5 nm bandwidth), $\lambda_{\text{em}} = 515$ nm (5 nm bandwidth) using a Tecan Spark multimode plate reader. Wells were considered suitable if no increasing fluorescence was observed (which would indicate substrate cleavage and thus RNase contamination).

After confirming that the substrate was free of contamination, an equal volume of the 2× enzyme/2× compound mixture was added to afford a final reaction composition of 1× = 40 pM enzyme, 1× compound dilution (ranging from 100 µM to 100 pM), and 80 nM substrate. Reactions were incubated at 25 °C for 25 min with continuous monitoring and a 3 s gentle plate shake once per plate read (approximately once every 45 s). Following the end of the monitoring period, excess RNase A (approximately 5 µM) was added to immediately cleave all substrate and generate maximum fluorescence. These maximum fluorescence readings were used to verify that all reaction wells received an equivalent amount of substrate.

The enzymatic reaction remained linear during the entire monitoring period; thus, linear regression of each entire 25 min dataset was performed to determine initial reaction rates in units of fluorescence intensity (in RFU) per second. Using GraphPad Prism v6, the initial rates for one compound were directly fitted to a one-site binding equation to yield the K_i of each compound:

$$\left(\frac{\Delta F}{\Delta t}\right)_{\mathrm{I}} = \left(\frac{\Delta F}{\Delta t}\right)_{0} \left(1 - \frac{[\mathrm{I}]}{K_i + [\mathrm{I}]}\right) \tag{1}$$

where:

$\left(\frac{\Delta F}{\Delta t}\right)_{\mathrm{I}}$: the initial reaction rate of all samples in which inhibitor compound is present

$\left(\frac{\Delta F}{\Delta t}\right)_{0}$: the initial reaction rate of RNase A in the absence of inhibitor compound

[I]: the molar concentration of inhibitor compound

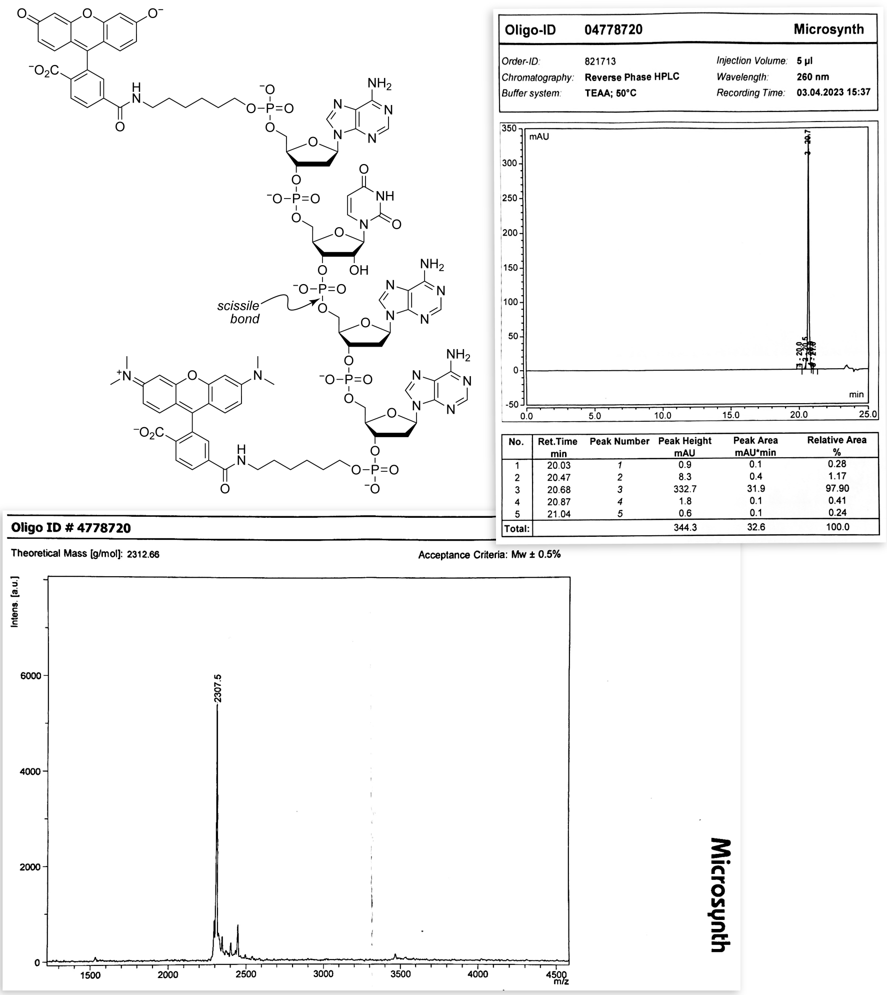

Figure S40: Structure of 6-FAM-dArU$(dA)_2$-6-TAMRA, which is the fluorogenic substrate for assays of ribonucleolytic activity and has only one scissile phosphodiester bond. The cleavage of that bond increases the fluorescence of the FAM moiety with $\lambda_{ex} = 493 \pm 5$ nm and $\lambda_{em} = 515 \pm 5$ nm.[S7] Insets: reversed-phase HPLC chromatogram (top) and MALDI-TOF mass spectrum (bottom) of synthetic 6-FAM-dArU$(dA)_2$-6-TAMRA.

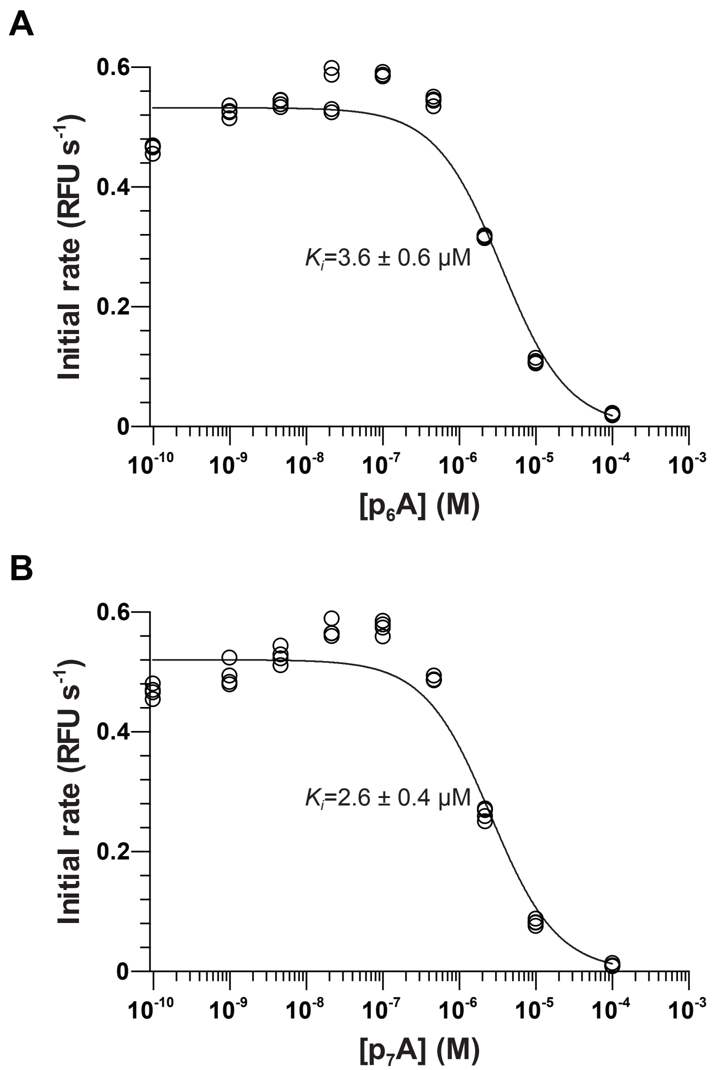

Figure S41: RNase A activity inhibition curves for (A) p_6A and (B) p_7A. These measurements were assayed in n=4 separate reaction wells.

The corresponding inhibition curves for p_6U and p_7U are shown in the main text.

S5 X-ray Protein Crystallography

Ribonuclease A was dissolved in nuclease-free water at a concentration of 20 mg/mL. Crystals were grown as previously described by 2-fold dilution of hanging drops (4 μL final) over a mother liquor of 20 mM sodium citrate buffer, pH 6.0, containing PEG 4000 (20% v/v).[S8] Crystals grew within a week to a size of roughly 0.6 × 0.6 × 0.1 mm. Stocks of ligands were prepared at 50 mM in mother liquor containing PEG 4000 (30% v/v) and glycerol (20% v/v). Crystals were soaked in with the ligand solution for 12-36 h. Crystals were cryo-protected by the mother liquor containing 20% glycerol. The crystallographic measurements were performed at 110 k using a Bruker D8 Venture Kappa Duo diffractometer equipped with a Bruker Photon2 CPAD detector (Cu-$K\alpha$ radiation, λ = 1.54178 Å). In each case, a specimen of suitable size and quality was selected and mounted onto a nylon loop. Integrated intensity information for each reflection was obtained by reduction of the data frames with SAINT[S9] implemented in the program APEX4 software. The semiempirical method SADABS was applied for the absorption correction.[S10] RNase A·ligand structures were solved by molecular replacement using the Phase program implemented in Phenix.[S11] The atomic coordinates of protein atoms from RNase A structure (1afu) were used as starting model.[S8] And the solved structures were refined with phenix.refine, and model building was conducted COOT.[S11,S12] Once a satisfactory apo structure was built, density consistent with ligands bound to the active site was found. Restraints for the ligands were prepared with eLBOW and LigandFit in Phenix. And then, the coordinates of the ligands were placed with the apo structure in COOT. The data has been deposited with the Protein Data Bank (PDB). 8ggg (RNase A·$\mathbf{p_6A}$) 8fhm (RNase A·$\mathbf{p_6U}$) 8s96 (RNase A·$\mathbf{p_7A}$) 8gc9 (RNase A·$\mathbf{p_7U}$) contain the supplementary crystallographic data for this paper.

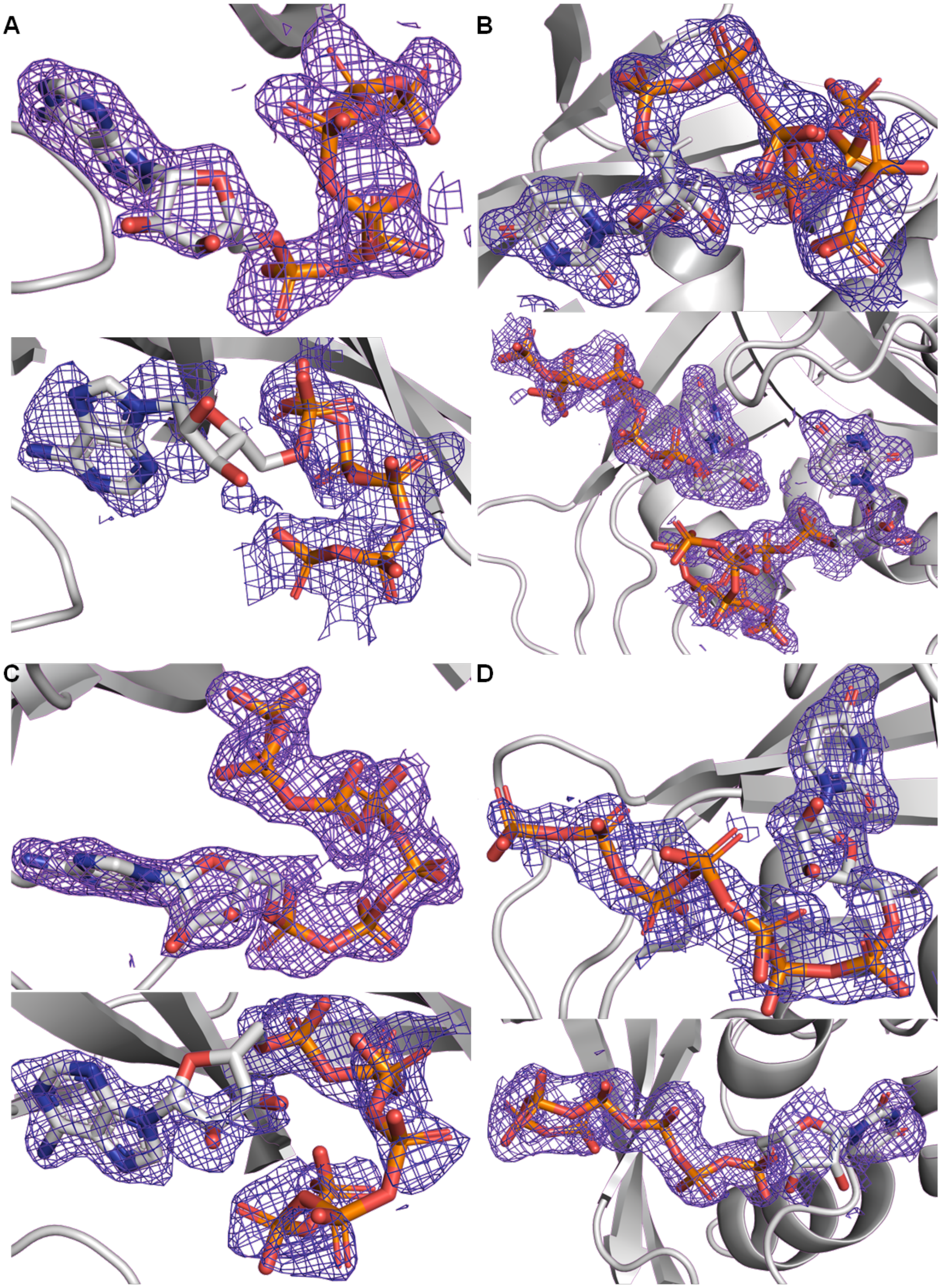

Figure S42: Densities of ligands bound to RNase A. Meshes are depicted of 2Fo – Fc maps contoured a 1σ or 0.7σ for (A) $\mathbf{p_6A}$, (B) $\mathbf{p_6U}$, (C) $\mathbf{p_7A}$, (D) $\mathbf{p_7U}$ of both chain A (top) and chain B (bottom).

S6 Computational Details

All calculations were carried out using a development version of the ORCA code based on version 5.0.[S13]

S6.1 HF-3c Binding Energy Calculations

To reduce the number of local coupled cluster calculations, we decided to perform a preliminary analysis at a more approximated level of theory to identify the preferential binding sites for each ligand. Accordingly, P-L binding energies were computed at the HF-3c[S14] level of theory using a supramolecular approach on all the experimentally detected ligand-active site combinations. For these calculations, the experimental crystal structures were used. The hydrogen positions were optimized using the GFN1-xTB method.[S15] Solvation effects were included implicitly at the C-PCM(water) level.[S16,S17] All the atoms of the ligands and of the protein (about 2000) were explicitly included in these calculations. In all cases, the ligands were found to bind preferentially to chain A. Specifically, $\mathbf{p_2U}$, $\mathbf{p_5U}$ and $\mathbf{p_6U}$ preferentially bind to chain A2, chain A2 and chain A1, respectively. All other ligands show a single site of interaction within chain A.

S6.2 ONIOM Binding Energy Calculations

For the sites showing the strongest P-L interaction at the HF-3c level, P-L binding energies were refined using a two-layer ONIOM method.[S18] In this approach, the full system is divided into: (i) the model subsystem, containing the ligand and the key residues in the binding site, and (ii) the environment, containing the rest of the protein and solvent. The model subsystem was treated at the DLPNO-CCSD(T)[S19] level of theory (denoted here-

after as the "high" level), while the environment was treated at the HF-3c level (denoted hereafter as the"low" level). Solvation effects were included at the C-PCM(water) level, as detailed below. The following residues were included in the model subsystem, which contains about 300 atoms: Lys7, Val43, Asn44, Thr45, Glu111, Glu11, His12, Arg10, Asn67, Lys66, His119, Phe120, Arg39, Gln69, Ser123, Lys41, Asn71. These are all the residues displaying short contacts with at least one ligand in the series. The link atom approach was used for the covalent bonds at the boundary between the model subsystem and the environment.

The DLPNO-CCSD(T) calculations on the model subsystem were carried out with NormalPNO settings using the def2-TZVP basis set in conjunction with matching def2/C auxiliary basis sets. The RIJCOSX approximation was used in the HF part (the def2/J basis set was used). A conservative integration grid was used (the default integration grid of ORCA 5.0).

The "subtractive" scheme was used in the ONIOM calculations:

$$E_{ONIOM} = E_{(CC,model)} + E_{(HF3c,full)} - E_{(HF3c,model)} \tag{2}$$

where $E_{CC,model}$ denotes the DLPNO-CCSD(T) energy of the model subsystem, while $E_{HF3c,full}$ and $E_{HF3c,model}$ are the HF-3c energies of the full system and of the model subsystem, respectively.

Within the ONIOM framework, the electrostatic interaction between the model system and the environment is typically modeled using embedding approaches. Among them, mechanical and electronic embedding are two popular strategies. In the former, the electrostatic interaction is only computed in the real system calculation. In contrast, in the electronic embedding, the electrostatic interaction between the model subsystem

and the environment is incorporated at some approximate level in the model subsystem calculation to account for polarization effects. A common strategy in this context is to replace the atoms in the environment with atomic charges in the model system calculation at the higher level of theory. These are obtained from a full system calculation at the lower level of theory using a population scheme.

In the current study an electronic embedding scheme was used. When such scheme is used in conjunction with C-PCM, the are various strategies for dealing with the coupling between the protein charge distribution and the continuum.[S20] In the present case, we opted for a straightforward yet accurate approach in which C-PCM charges are used to represent both the solvent and the protein environment in the subsystem calculation. The dielectric constant of water was used as a model. The accuracy of this strategy, which reduces significantly the complexity of the problem with respect to a standard electronic embedding strategy, was demonstrated by comparing the P-L binding energies obtained for the full system at the HF-3c/C-PCM(water) level ($\Delta E_{(HF3c,full)}$) with those obtained from the embedded HF-3c calculations on the model system $\Delta E_{(HF3c,model)}$. It was found that $\Delta E_{(HF3c,full)} - \Delta E_{(HF3c,model)}$ amounts to just 5-6% of $\Delta E_{(HF3c,full)}$, as shown in Table S1. These results indicate that ONIOM P-L binding energies are reasonably well-converged with respect to the size of the model subsystem.

Table S1: P-L biding energies computed for the full system $\Delta E_{(HF3c,full)}$ and for the embedded model subsystem $\Delta E_{(HF3c,model)}$ at the HF-3c level of theory. Our best estimate obtained using the ONIOM approach at the DLPNO-CCSD(T)/HF-3c level is also reported (in bold) for comparison. The latter also includes geometric relaxation effects. See text for details. All energies are in kcal/mol.

Ligand	$\Delta E_{(HF3c,full)}$	$\Delta E_{(HF3c,model)}$	$\Delta E_{(HF3c,full)} - \Delta E_{(HF3c,model)}$	$\mathbf{\Delta E_{(ONIOM)}}$
$\mathbf{p_2U}$	-60	-56	-5	$\mathbf{-47}$
$\mathbf{p_5U}$	-118	-111	-7	$\mathbf{-77}$
$\mathbf{p_6U}$	-111	-103	-7	$\mathbf{-69}$
$\mathbf{p_7U}$	-100	-96	-5	$\mathbf{-57}$

S6.3 Geometry Optimizations and Ligand Strain Energies

In addition to noncovalent interactions, the affinity of a ligand towards a protein is influenced by thermodynamic corrections and deformation free energy effects. The latter originate from structural rearrangements in both the ligand and protein that may occur during the binding process. While the precise modeling all of these complex effects exceeds the scope of the current study, the energy penalty associated with the geometric distortion of the ligand can be easily incorporated within a supramolecular approach. Specifically, the ligand strain energy (ΔE_{strain}) can be computed as the difference between the energy of the ligand in the structure it has in the protein minus the energy it has in its unbound geometric ground state. The latter was estimated by fully relaxed geometry optimizations at the HF-3c/C-PCM(water), while the calculation of the former requires a more sophisticated approach.

Due to the large size of the systems considered in this work, fully relaxed geometry optimizations for the protein-ligand adduct are not feasible at the ONIOM level. Hence, to incorporate the key geometric effects into our binding energy estimates, we devised an *ad hoc* strategy. Initially, the atomic positions in the embedded model subsystem were optimized at the HF-3c level in the presence of geometrical constraints. Our goal was to preserve the relative orientation of ligands and residues as determined experimentally while allowing at the same time for some geometry relaxation at the chosen level of theory. For this purpose, we selectively optimized only the position of the heteroatoms in the model system, thereby enabling the phosphate backbone to relax without altering the experimental pattern of noncovalent interactions (see Figure S43). The optimized structures were used to compute the ΔE_{strain} values, which are reported in Table S2. These figures were added to the ONIOM estimate of P-L binding energies to obtain the

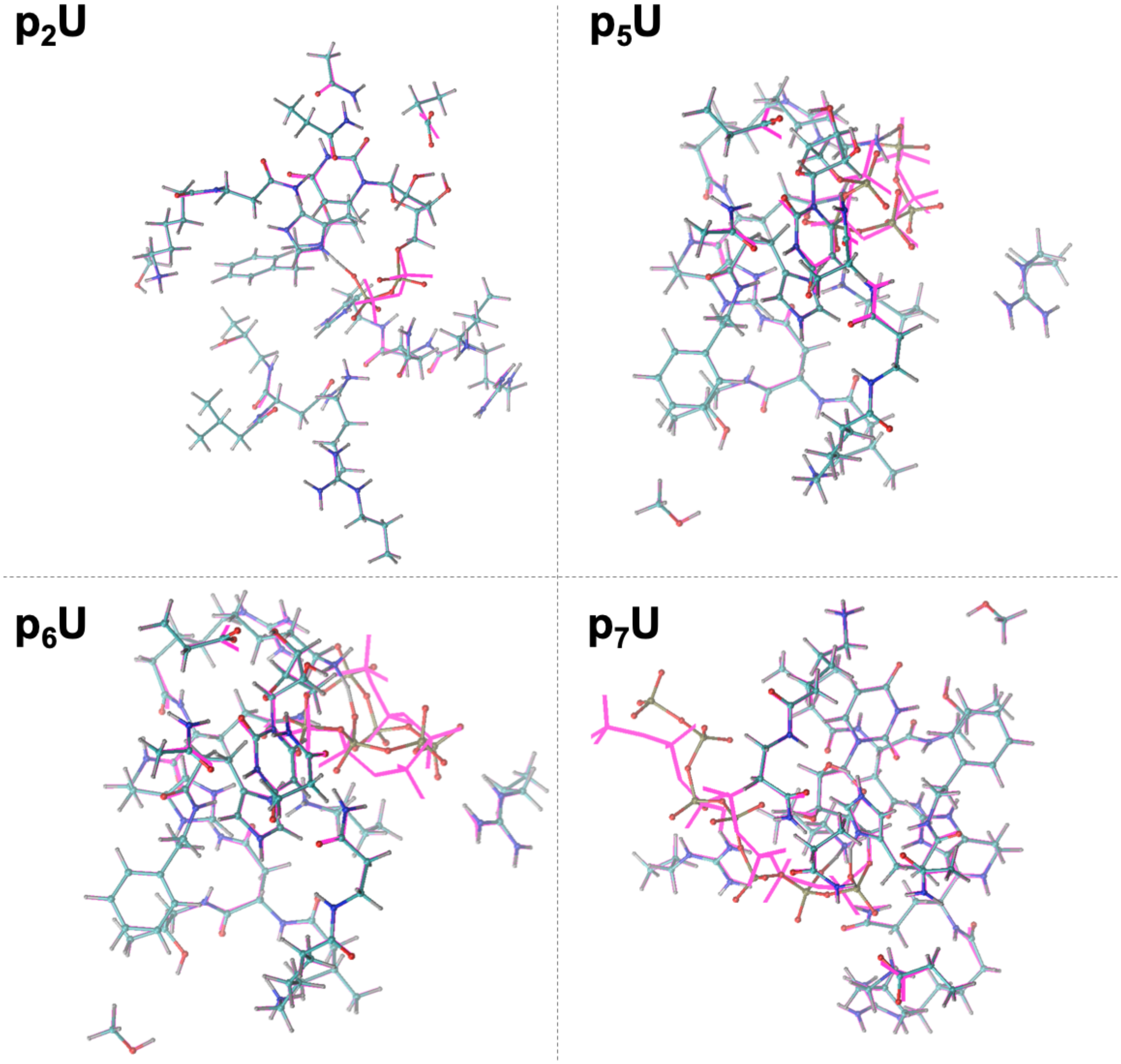

Figure S43: For $\mathbf{p_2U}$ $\mathbf{p_5U}$, $\mathbf{p_6U}$, and $\mathbf{p_7U}$, overlay of starting geometries, as derived from the experimental crystal structures (pink wireframe), and constrained geometry optimizations (embedded model subsystem only). In the optimized geometries, the ligands and residues are shown as ball and stick models, with cyan carbon atoms.

values reported in Table S1. Note that, in these calculations, $\Delta E_{(CC,model)}$ was computed using the optimized structures, while the $\Delta E_{(HF3c,full)} - \Delta E_{(HF3c,model)}$ correction was obtained from the experimental structures with optimized H positions, as discussed above.

Table S2: ΔE_{strain} energies for uridine-based ligands computed at the HF-3c/C-PCM(water) level. See text for details. All energies are in kcal/mol.

Ligand	ΔE_{strain}
$\mathbf{p_2U}$	13.2
$\mathbf{p_5U}$	14.0
$\mathbf{p_6U}$	29.1
$\mathbf{p_7U}$	23.5

S6.4 Local Energy Decomposition analysis and LED Maps

The LED analysis in its extension for multiple fragments[S21,S22] was used to shed light into the complex pattern of noncovalent interactions operating in these systems by allowing the decomposition of P-L binding energies at the ONIOM level (ΔE) into addictive contributions. The decomposed binding energy reads:

$$\Delta E = \Delta E^{L}_{tot} + \sum_{R} \Delta E^{R}_{tot} + \sum_{R} \Delta E^{(R,R)}_{tot} + \sum_{R} E^{(L,R)}_{tot} \tag{3}$$

Here, ΔE^{L}_{tot} and ΔE^{R}_{tot} represent the change in energy of the ligand and of the residues upon ligand-protein binding, respectively. In standard LED studies, these terms only account for electronic perturbation effects (the so called electronic preparation), while geometric perturbation effects are typically provided as separate terms (the so called geometric preparation). In this work, to obtain a simple pairwise-decomposition of the binding energy, we have incorporated into ΔE^{L}_{tot} both electronic and geometric (ΔE_{strain})

preparation effects. Finally, $\Delta E_{tot}^{(R,R)}$ represents the change in residue-residue interactions upon ligand binding, while $E_{tot}^{(L,R)}$ denotes the interaction of the ligand with the residues in the active site. A graphical representation of the LED terms in eq. 3 is provided by the LED interaction maps.[S21]

As mentioned above, solvent effects were modeled using the C-PCM approach. As expected from our previous work on DNA model systems,[S22] the contribution to the binding energy obtained from the C-PCM dielectric ΔE_{CPCM}, accounting for electrostatic interactions between the model subsystem and the environment, is large and positive. Its net effect is to counteract to a large extent the electrostatic interaction between the ligand and the other charged residues in the system. ΔE_{CPCM} cannot be easily decomposed within the LED scheme, and hence this term is typically provided separately in LED studies. However, since in the present case we are dealing with negatively charged ligands, whose interaction with the residues is to a large extent dominated by electrostatic forces, it makes physical sense to incorporate the contribution of the dielectric directly into the decomposition. This was achieved by scaling each element in a given LED map by the same factor, so that the sum of all the elements of each map gives the total binding energy incorporating the dielectric contribution. The ΔE_{strain} contribution to ΔE_{tot}^{L} was incorporated after the scaling.

S6.5 Computational Studies on Adenosine Derivatives

The methodology just described was also applied to study the binding of adenosine-based ligands to RNase A. Specifically, we considered $\mathbf{p_3A}$, $\mathbf{p_5A}$, $\mathbf{p_6A}$, and $\mathbf{p_7A}$ as ligands.

It was found found that all ligands preferentially bind to chain A, consistent with the uridine case. The optimized structures obtained from the constrained geometry optimizations for these systems are shown in figure S44, while the corresponding LED maps are

shown in figure S45.

Remarkably enough, all adenosine-based ligands show essentially the same pattern of noncovalent interactions with the residues in the active site. The only notable distinction was observed for **p$_7$A**, which exhibits a significantly stronger attractive interaction with Lys41 compared to the other ligands considered in this study. However, this interaction is partly counterbalanced by a considerable geometric preparation energy (see Table S3), which can be attributed to the convoluted conformation of **p$_7$A** in the active site.

Table S3: ΔE_{strain} energies for adenosine-based ligands computed at the HF-3c/C-PCM(water) level. See text for details. All energies are in kcal/mol.

Ligand	ΔE_{strain}
**p$_3$A**	22.7
**p$_5$A**	30.0
**p$_6$A**	23.8
**p$_7$A**	39.3

As a result, all adenosine-based ligands exhibit comparable binding energies, with a much smaller variance than uridine-based ligands. These results suggest that the trend in the experimental binding constants in this case is likely influenced by other thermostatically effects, such as conformational entropy. These effects likely favor the binding of the smaller ligands to RNase A.

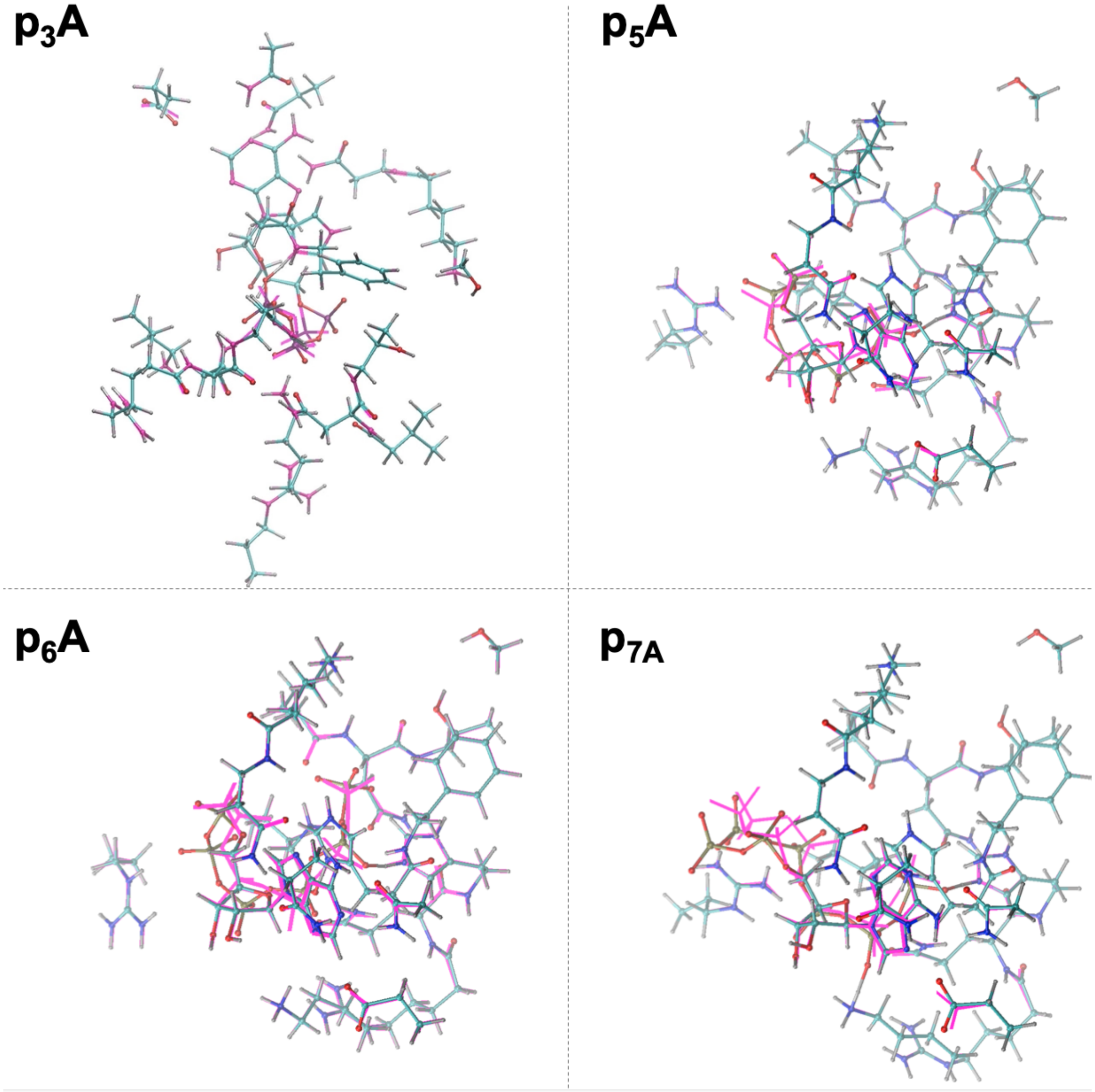

Figure S44: For $\mathbf{p}_3\mathbf{A}$ $\mathbf{p}_5\mathbf{A}$, $\mathbf{p}_6\mathbf{A}$, and $\mathbf{p}_7\mathbf{A}$, overlay of starting geometries, as derived from the experimental crystal structures (pink wireframe), and constrained geometry optimizations (embedded model subsystem only). In the optimized geometries, the ligands and residues are shown as ball and stick models, with cyan carbon atoms.

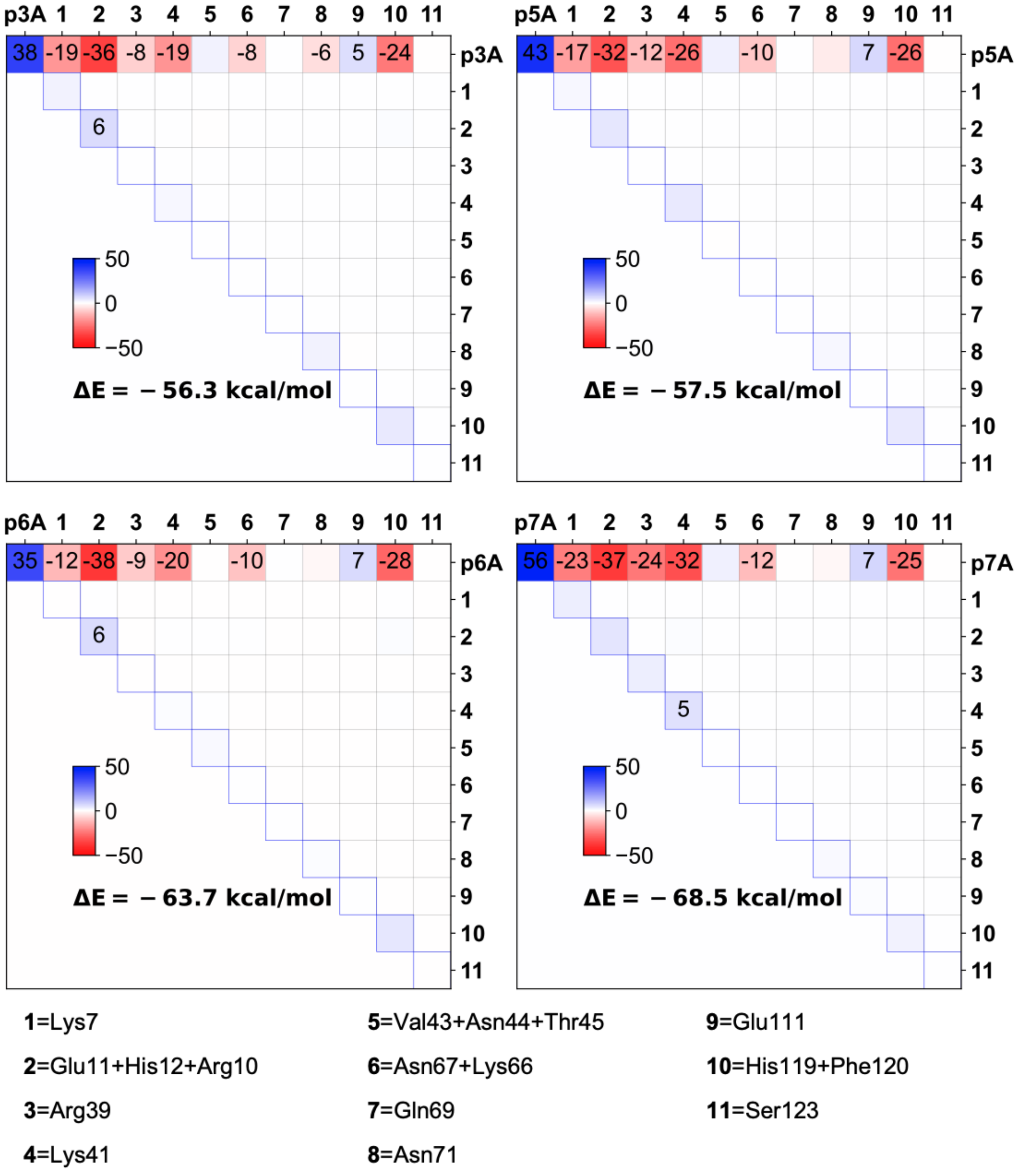

Figure S45: LED interaction maps for fragment-pairwise ligand-residue and residue-residue interactions associated with the binding of $\mathbf{p_3A}$, $\mathbf{p_5A}$, $\mathbf{p_6A}$, and $\mathbf{p_7A}$ to RNase A. Only the figures with absolute value larger than 5 kcal/mol are shown.

S7 References

References

(S1) Mohamady, S.; Taylor, S. D. Synthesis of Nucleoside Tetraphosphates and Dinucleoside Pentaphosphates via Activation of Cyclic Trimetaphosphate. *Org. Lett.* **2013**, *15*, 2612–2615.

(S2) Pangborn, A. B.; Giardello, M. A.; Grubbs, R. H.; Rosen, R. K.; Timmers, F. J. Safe and Convenient Procedure for Solvent Purification. *Organometallics* **1996**, *15*, 1518–1520.

(S3) Jiang, Y.; Chakarawet, K.; Kohout, A. L.; Nava, M.; Marino, N.; Cummins, C. C. Dihydrogen tetrametaphosphate, [P4O12H2]2-: synthesis, solubilization in organic media, preparation of its anhydride [P4O11]2- and acidic methyl ester, and conversion to tetrametaphosphate metal complexes via protonolysis. *J. Am. Chem. Soc.* **2014**, *136*, 11894–7.

(S4) Shepard, S. M.; Windsor, I. W.; Raines, R. T.; Cummins, C. C. Nucleoside Tetra- and Pentaphosphates Prepared Using a Tetraphosphorylation Reagent Are Potent Inhibitors of Ribonuclease A. *J. Am. Chem. Soc.* **2019**, *141*, 18400–18404.

(S5) Smith, B. D.; Soellner, M. B.; Raines, R. T. Potent Inhibition of Ribonuclease A by Oligo(vinylsulfonic Acid). *J. Biol. Chem.* **2003**, *278*, 20934–20938.

(S6) Cavaluzzi, M. J.; Borer, P. N. Revised UV extinction coefficients for nucleoside-5-monophosphates and unpaired DNA and RNA. *Nucleic Acids Res.* **2004**, *32*, e13.

(S7) Kelemen, B. R.; Klink, T. A.; Behike, M. A.; Eubanks, S. R.; Leland, P. A.; Raines, R. T. Hypersensitive substrate for ribonucleases. *Nucleic Acids Res.* **1999**, *27*, 3696–3701.

(S8) Leonidas, D. D.; Shapiro, R.; Irons, L. I.; Russo, N.; Acharya, K. R. Crystal Structures of Ribonuclease A Complexes with 5‘-Diphosphoadenosine 3‘-Phosphate and 5‘-Diphosphoadenosine 2‘-Phosphate at 1.7 Å Resolution. *Biochem.* **1997**, *36*, 5578–5588.

(S9) Sheldrick, G. SAINT (Version 6.02), SADABS (Version 2.03). *Bruker AXS Inc. Madison, WI* **2002**,

(S10) Sheldrick, G. SADABS version 2014/5. *Bruker AXC Inc., Madison, WI* **2014**,

(S11) Adams, P. D.; Afonine, P. V.; Bunkóczi, G.; Chen, V. B.; Davis, I. W.; Echols, N.; Headd, J. J.; Hung, L.-W.; Kapral, G. J.; Grosse-Kunstleve, R. W.; McCoy, A. J.; Moriarty, N. W.; Oeffner, R.; Read, R. J.; Richardson, D. C.; Richardson, J. S.; Terwilliger, T. C.; Zwart, P. H. *PHENIX*: a comprehensive Python-based system for macromolecular structure solution. *Acta Crystallogr. D Biol. Crystallogr.* **2010**, *66*, 213–221.

(S12) Emsley, P.; Lohkamp, B.; Scott, W. G.; Cowtan, K. Features and development of *Coot*. *Acta Crystallogr. D Biol. Crystallogr.* **2010**, *66*, 486–501.

(S13) Neese, F. Software update: The ORCA program system—Version 5.0. *Wiley Interdiscip. Rev. Comput. Mol. Sci.* **2022**, *12*, e1606.

(S14) Sure, R.; Grimme, S. Corrected small basis set Hartree-Fock method for large systems. *J. Comput. Chem* **2013**, *34*, 1672–1685.

(S15) Grimme, S.; Bannwarth, C.; Shushkov, P. A robust and accurate tight-binding quantum chemical method for structures, vibrational frequencies, and noncovalent interactions of large molecular systems parametrized for all spd-block elements (Z= 1–86). *J. Chem. Theory Comput.* **2017**, *13*, 1989–2009.

(S16) Barone, V.; Cossi, M. Quantum calculation of molecular energies and energy gradients in solution by a conductor solvent model. *J. Phys. Chem. A* **1998**, *102*, 1995–2001.

(S17) Garcia-Ratés, M.; Neese, F. Effect of the Solute Cavity on the Solvation Energy and its Derivatives within the Framework of the Gaussian Charge Scheme. *J. Comput. Chem.* **2020**, *41*, 922–939.

(S18) Chung, L. W.; Sameera, W.; Ramozzi, R.; Page, A. J.; Hatanaka, M.; Petrova, G. P.; Harris, T. V.; Li, X.; Ke, Z.; Liu, F.; others The ONIOM method and its applications. *Chem. Rev.* **2015**, *115*, 5678–5796.

(S19) Riplinger, C.; Pinski, P.; Becker, U.; Valeev, E. F.; Neese, F. Sparse maps—A systematic infrastructure for reduced-scaling electronic structure methods. II. Linear scaling domain based pair natural orbital coupled cluster theory. *J. Chem. Phys.* **2016**, *144*.

(S20) Vreven, T.; Mennucci, B.; da Silva, C. O.; Morokuma, K.; Tomasi, J. The ONIOM-PCM method: Combining the hybrid molecular orbital method and the polarizable continuum model for solvation. Application to the geometry and properties of a merocyanine in solution. *J. Chem. Phys.* **2001**, *115*, 62–72.

(S21) Beck, M. E.; Riplinger, C.; Neese, F.; Bistoni, G. Unraveling individual host–guest interactions in molecular recognition from first principles quantum mechanics: In-

sights into the nature of nicotinic acetylcholine receptor agonist binding. *J. Comput. Chem.* **2021**, *42*, 293–302.

(S22) Altun, A.; Garcia-Ratés, M.; Neese, F.; Bistoni, G. Unveiling the complex pattern of intermolecular interactions responsible for the stability of the DNA duplex. *Chem. Sci.* **2021**, *12*, 12785–12793.
